# Temporal uncoupling of radial glia lineage progression in cortical organoids

**DOI:** 10.1038/s41586-026-10916-7

**Published:** 2026-08-12

**Authors:** Melissa Stouffer, Osvaldo A. Miranda, Florian M. Pauler, Fabrizia Pipicelli, Carmen Streicher, Giselle Cheung, Simon Hippenmeyer

**Affiliations:** 1 Institute of Science and Technology Austria (ISTA), Klosterneuburg, Austria; 2 Present Address: a:head bio AG, Vienna, Austria; 3 Present Address: Department of Clinical and Biomedical Sciences, University of Exeter, Exeter, UK

**Keywords:** Neural stem cells, Developmental neurogenesis

## Abstract

Radial glial progenitors (RGPs) produce all excitatory neurons in the developing cerebral cortex. Mosaic analysis with double markers (MADM)-based lineage tracing in vivo has revealed a quantitative framework of RGP lineage progression^1^. Here we established MADM technology^2,3^ in mouse embryonic stem cells to probe RGP lineage progression in a self-organizing cortical organoid system. We found that RGPs exhibit a high level of plasticity in proliferative potential in organoids rather than strict temporally stereotyped lineage progression as observed in vivo. RGPs in organoids showed increased lineage restriction, diminishing cell-type diversity in clones of cortical projection neurons, despite uniform single-cell transcriptional signatures of RGPs and a unitary lineage trajectory. Thus, critical non-cell-autonomous cues that are absent in self-organizing systems and/or the genuine stem cell niche are essential for faithful temporal control of RGP lineage progression and the generation of clonal cortical cell-type diversity.

## Abstract (web summary)

RGP lineage tracing in stem-cell-derived organoids shows highly plastic proliferation and reduced clonal neuronal diversity, indicating that missing non-cell-autonomous niche cues are essential for faithful temporal control of cortical lineage progression.

## Main

The cerebral cortex is the seat of cognitive brain function and is composed of an enormous number and diversity of neurons and glial cells. Neural stem cells (NSCs), including RGP cells^4,5^ and their cognate cell lineages, generate and comprise all major neocortical projection neuron classes and macroglia^6–8^. Systematic clonal analysis at single-cell level in situ using MADM has provided an inaugural quantitative and temporally stereotyped framework of RGP lineage progression in mice in vivo^1,9^. Nascent RGPs initially undergo symmetric proliferative divisions, expanding their pool. Subsequent to a predictable number of proliferative divisions, and at a defined developmental stage, RGPs switch to asymmetric neurogenic division. RGPs in mouse generate projection neurons in units of 8–9 neurons, whereby birth order defines cell fate and laminar position. Following neurogenesis, a fraction of RGPs adopt gliogenic potential to produce astrocytes and oligodendrocytes^1^. RGP lineage progression proceeds in a strictly linear manner with sequential, largely non-overlapping and consecutive developmental timeframes. Faithful RGP proliferation behaviour and lineage progression are essential for the generation of a cerebral cortex of correct size and cell-type diversity^10–13^. The fundamental principles that instruct the transitions along RGP lineage and their chronological neurogenic or gliogenic potential are still unknown, although cell-intrinsic epigenetic and genetic cues seem to be critical. Indeed, cortical progenitors^14–16^ and mouse embryonic stem cells (mESCs) programmed to cortical lineage^17^ recapitulate limited RGP lineage motifs in isolated cell culture, and thus in the absence of an endogenous stem cell niche. Furthermore, recent advances in recreating major stages of embryonic development in vitro have revealed that self-organizing principles are sufficient for pluripotent embryonic stem cell lineage progression within all three germ layers^18–20^. Here, we focus specifically on cortical organoid systems in which self-organization appears to be the major driver for the concerted three-dimensional structural assembly of cortical structures, with all major cell types, from a set of embryonic NSCs, closely recapitulating developmental processes and cortical tissue-specific features^21–24^. However, whether and how self-organization can instruct committed multipotent progenitor cells and orchestrate temporally stereotyped NSC lineage progression in order to produce faithful quantitative and qualitative postmitotic cell fates and cell-type diversity is not known. Here we utilized lineage tracing with true single-cell resolution in situ and in silico to decipher the neurogenic RGP proliferation behaviour in an in vitro organoid system. We found that in self-organizing cortical organoids, RGP lineage progression contrasts in specific aspects with the in vivo concept of linear, temporally stereotyped progression of RGP cell lineage. The genuine stem cell niche therefore seems to be essential for faithful temporal control of RGP lineage progression and the generation of cortical cell-type diversity at the clonal level.

## Organoids model in vivo development

**Fig. 1:**
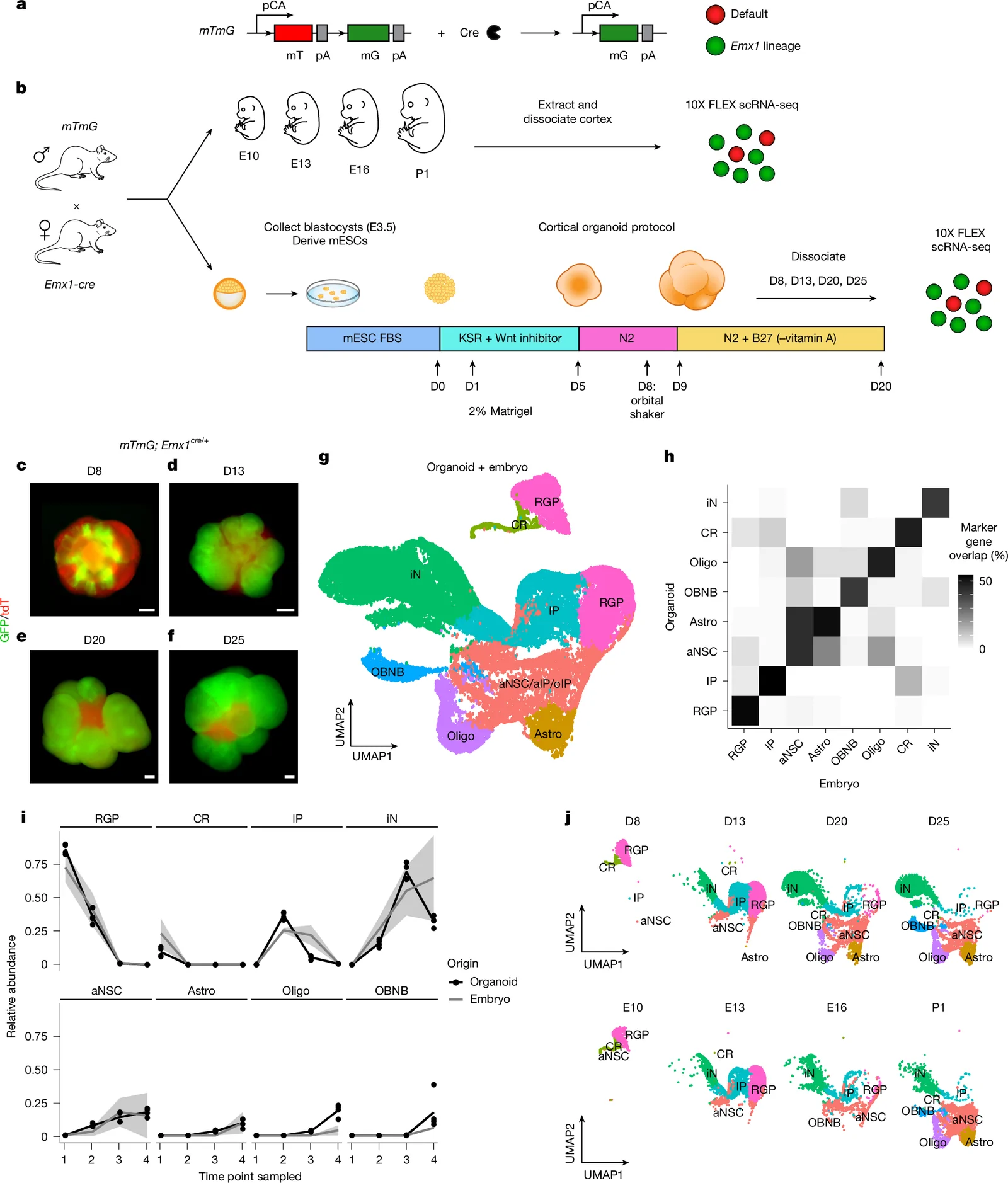
A mouse cortical organoid system recapitulates major aspects of in vivo corticogenesis at transcriptional single-cell level. **a**, Genetic strategy to permanently label cortical *Emx1* lineage with GFP using a *mTmG* reporter and *Emx1-cre* driver. **b**, Schematic illustrating the experimental scRNA-seq approach to trace RGP lineage progression at the transcriptional level. Mice with *mTmG* reporter and *Emx1-cre* transgenes were crossed to generate *mTmG*^*+/−*^;*Emx1*^*cre/+*^ embryos (top) and blastocysts (bottom) for downstream processing (Methods). B27, B27 supplement; FBS, fetal bovine serum; KSR, knockout serum replacement; N2, N2 supplement. **c**–**f**, Wide-field images of *mTmG*^*+/−*^;*Emx1*^*cre/+*^ organoids at D8 (**c**), D13 (**d**), D20 (**e**) and D25 (**f**). Note, GFP^+^ cells reflect derivatives of *Emx1*^*+*^ progenitor cell lineages whereas tdT^+^ cells were derived from *Emx1*^*−*^ progenitors. Organoids of this genotype were generated in at least three independent batches. Scale bars, 200 µm. **g**, UMAP and cell-type annotation of integrated organoid and in vivo mouse cells within the *Emx1*^*+*^ neuronal lineage: RGPs; Cajal–Retzius cells (CR); intermediate progenitors (IP); immature neurons (iNs); adult NSCs (aNSC), astrocyte intermediate progenitors (aIP) and oligodendrocyte intermediate progenitors (oIP); astrocytes (astro); oligodendrocytes (oligo); and olfactory bulb neuroblasts (OBNB). **h**, Overlap analysis of top 100 marker genes identified for each indicated cell type in organoid and in vivo mouse embryo. **i**, Relative abundance of cell types as described in **g** at distinct organoid differentiation and mouse in vivo time points. Note that aNSC also includes aIPs and oIPs. Data are represented as mean and individual data points of four biological replicates (organoid) or mean ± s.d. for three embryo datasets (this study and refs. ^25,26^). Numbers on the *x* axis indicate developmental age (1, E10–E11 or D8; 2, E13 or D13; 3, E16 or D20; 4, E18–P1 or D25). **j**, UMAP as depicted in **g** but separated by developmental time point for organoids (top) and embryos (bottom).

To establish an in vitro system that allows the study of *Emx1*^*+*^ RGPs and their entire prospectively labelled lineage-related progeny, we conceived a genetic strategy to target the *Emx1*^*+*^ lineage (which generates most, if not all, cortical excitatory neurons and macroglia in vivo) in differentiating cortical organoids (Fig. 1a,b). We first crossed the *Emx1-cre* driver to the *mTmG* reporter system to label all cells derived from *Emx1*^*+*^ lineages with GFP in a tdTomato (tdT) background (that is, all *Emx1*^*−*^ cells), isolated *mTmG*^*+/−*^;*Emx1*^*cre/+*^ blastocysts at E3.5, and derived mESCs. Immunohistochemistry using pluripotency markers (OCT3/4 and NANOG) and G-band karyotyping qualitatively validated the newly established mESC lines (Supplementary Fig. 1). Next, we generated *mTmG*^*+/−*^;*Emx1*^*cre/+*^ cortical organoids, isolated samples in a time course at differentiation day 8 (D8), D13, D20 and D25 (Fig. 1b–f), and subjected the samples to 10X FLEX single-cell RNA sequencing (scRNA-seq) (two independent cell lines and two batches each; Methods).

**Extended Data Fig. 1:**
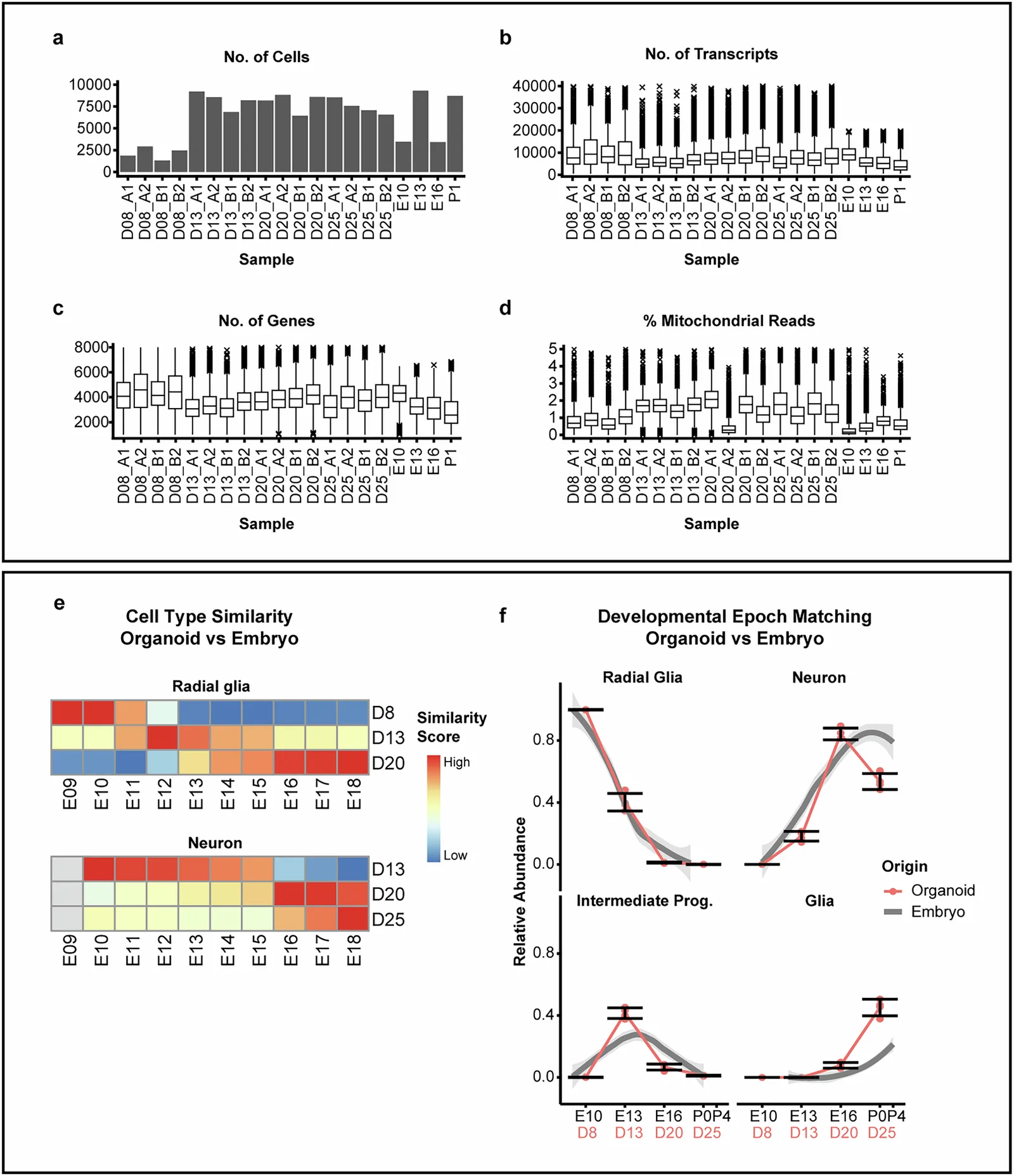
Quality control and developmental age matching in organoid and in vivo mouse scRNA-Seq data sets. **a-d**, Quality control parameters after filtering for *Gfp* expressing cell clusters. Number of high quality cells in each replicate (**a**), number of transcripts (**b**), number of genes (**c**), and number of mitochondrial reads per cell (**d**), across all replicates and samples. ‘n’ corresponds to number of cells shown in panel (**a**). (**b-d**) The centre line indicates the median, the box limits show the 25–75% IQR, whiskers extend to include the furthest data points within 1.5x the IQR from box edges, and data points outside this range are represented as dots. **e**, Heatmaps show transcriptome similarities between the ref. ^25^ (E9-E18) and organoid time points for RGPs (top) and neurons (bottom) (see ‘*Matching* in vivo *mouse developmental age to organoid differentiation stage*’ in Methods). **f**, Overlay of relative cell type abundance in organoid derived data from this study and two reference data sets^25,26^. Red lines with error bars depict the mean and standard deviation of 4 biological replicates for each developmental time point in organoid data. Grey line with shaded area shows a smoothed line (Local Polynomial Regression Fitting) with 90% confidence interval from 10/11 data points (E10-E18, P1, P4/E9-E18) from reference data sets^25,26^, respectively. Note that the x-axis only shows the best matching reference data points.

**Extended Data Fig. 2:**
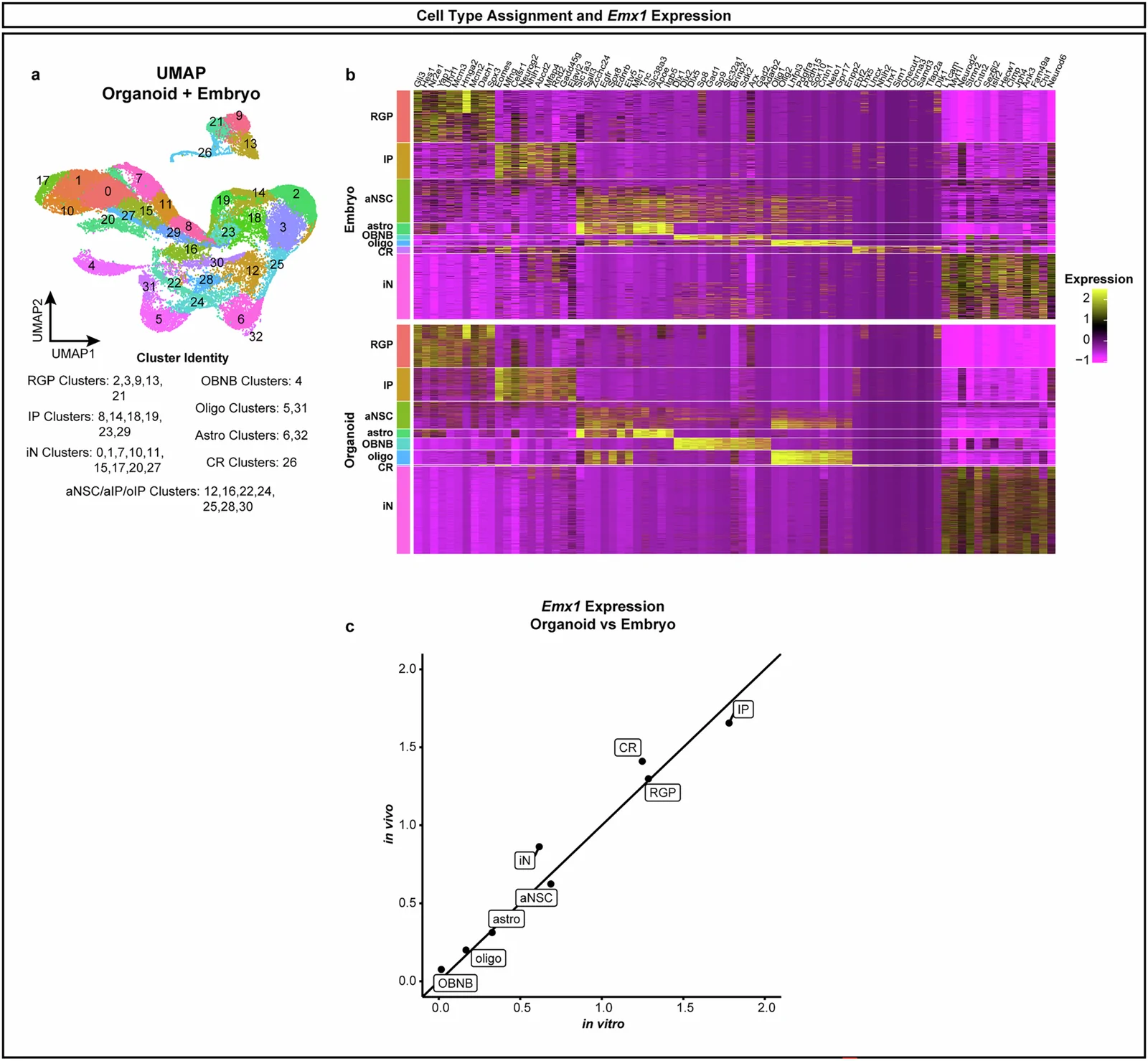
scRNA-seq indicates transcriptional cell type similarity of *Emx1*^+^ lineage across development in cortical organoids and mouse in vivo. **a**, Integrated UMAP derived from scRNAseq in *mTmG*^*+/−*^;*Emx1*^*Cre/+*^ organoids and in vivo mouse cortex. Colors and numbers indicate unbiased Seurat clustering. Association of Seurat clusters with cell types is shown below the UMAP. **b**, Expression of top common marker genes for each cell type in vivo mouse cortex (top) and in organoid (bottom). All available data sets for organoid/embryo were combined for the analysis. Cell type abbreviations: Radial glia progenitor (RGP), Intermediate progenitors (IPs), adult neural stem cells (aNSC), astrocytes (astro), olfactory bulb neuroblasts (OBNB), oligodendrocytes (oligo), Cajal-Retzius cells (CR), and immature neurons (iN). Note that aNSC also include astrocyte intermediate progenitors (aIPs) and oligodendrocyte intermediate progenitors (oIPs). **c**, Pseudobulk expression of *Emx1* in each cell type of organoid and in vivo mouse tissue, shown as a dot plot.

After initial quality control of the scRNA-seq dataset, we identified *Gfp*^*+*^ cells from the *Emx1*^*+*^ lineage using dimensionality reduction and visualization using uniform manifold approximation projection (UMAP) (Supplementary Figs. 2 and 3). To identify the closest matching in vivo brain region for organoid cells, we used a spatially resolved in vivo scRNA-seq dataset^25^, which confirmed that only cells with a forebrain identity expressed *Gfp*. After extracting *Gfp*^*+*^ cells and removing cells with a transcriptional stress signature, we retained 1,302 to 9,220 high-quality cells per individual sample (Extended Data Fig. 1a–d). Next, we systematically compared in vitro organoid differentiation to corresponding in vivo embryo development. To align developmental age, we first made use of two previously published scRNA-seq datasets of mouse cortex development^25,26^, focusing on four key cell types: RGPs (declining abundance during development), intermediate progenitors (transient increase during development), neurons (increasing abundance during early development) and glia (increase towards end of embryonic development). This analysis revealed that: (1) D8, D13, D20 and D25 of organoid differentiation matched with embryonic day 10 (E10), E13, E16 and postnatal day 1 (P1) of mouse development, respectively; and (2) the qualitative trajectory (shape of the curve) of in vivo and in vitro development was remarkably similar, indicating comparable developmental dynamics (Extended Data Fig. 1e,f). On the basis of these findings, we generated 10X FLEX scRNA-seq datasets for *mTmG*^*+/−*^;*Emx1*^*cre/+*^ embryos at E10, E13, E16 and P1 (Fig. 1b) and produced UMAPs for clustering; *Gfp* detection confirmed specific *Emx1* expression in forebrain tissue (Supplementary Figs. 4 and 5), and we retained 3,422 to 9,314 high-quality cells per time point (Extended Data Fig. 1a–d). Next, we directly compared both in vitro and in vivo datasets through data integration (Extended Data Fig. 2a). On the basis of marker gene expression, we identified all reported major cell types originating from *Emx1*-expressing RGPs in the developing cerebral cortex (Fig. 1g), and confirmed highly similar marker gene expression in comparable cell types in cortical organoids and embryos (Fig. 1h and Extended Data Fig. 2b,c). Finally, we assessed cell-type abundance and found high reproducibility between organoid batches (Fig. 1i) and remarkable overlap between matching organoid and embryo developmental time points (Fig. 1i,j). In conclusion, we show that cortical organoid and *Emx1*^*+*^ lineage differentiation at single-cell resolution closely resembles in vivo corticogenesis.

## MADM in the cortical organoid system

**Fig. 2:**
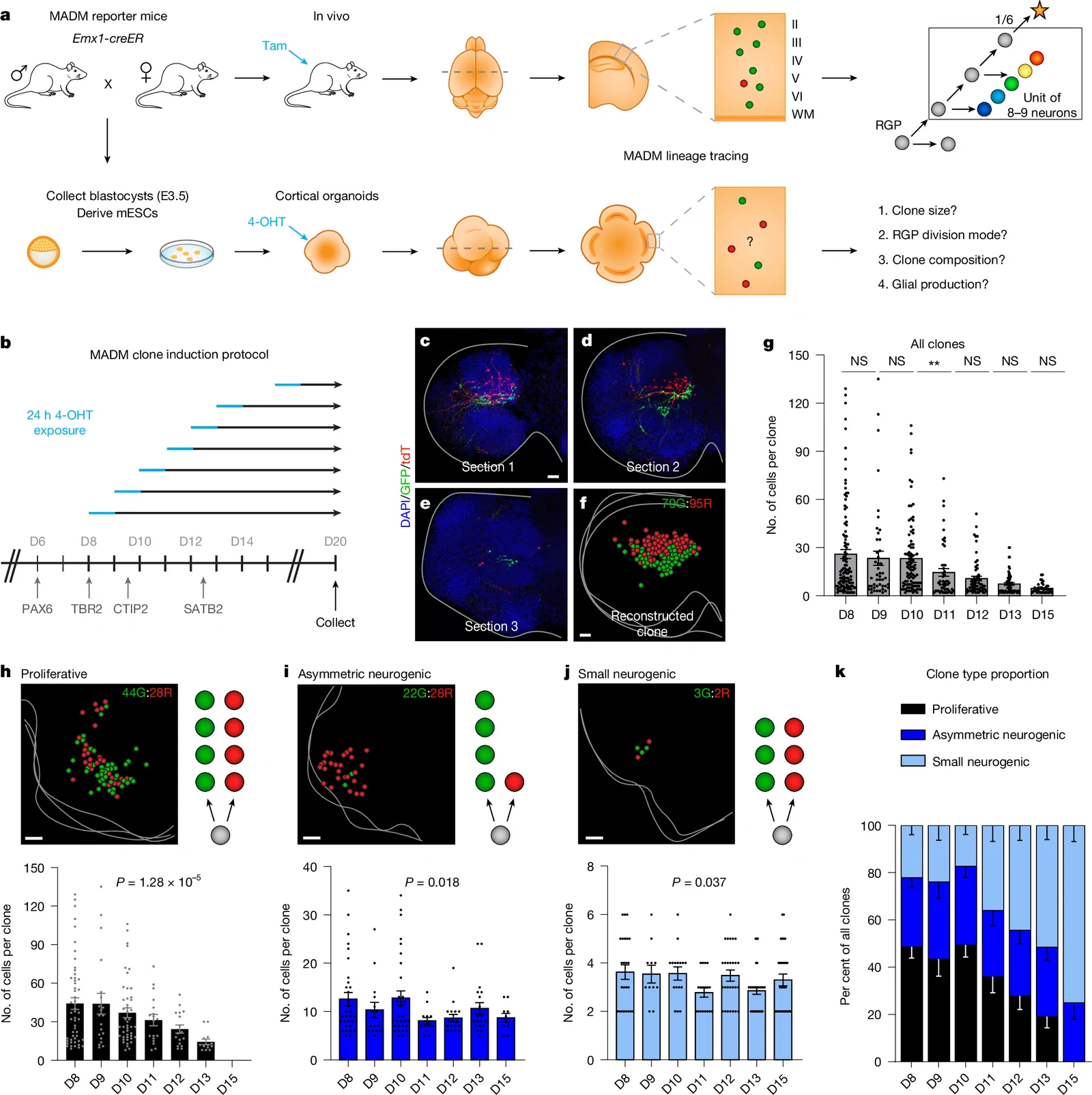
RGP cell lineage progression in cortical organoid system differs from the in vivo context. **a**, Illustration of the MADM system for neocortical stem cell lineage tracing in vivo (top, from refs. ^1,56^) and in vitro (bottom, this study) (Methods, ‘Induction of MADM clones in organoids’). Top right, one in six progenitors produce glia (star). Tam, tamoxifen. **b**, MADM clone induction protocol with 4-OHT administration at different developmental time points and collection at D20 for analysis. Onset of markers defining RGPs (PAX6), intermediate progenitors (TBR2) and the major cortical projection neuron classes are indicated (see Extended Data Fig. 4). **c**–**f**, Representative MADM clone. MADM-labelled cells across three sections (**c**–**e**) were reconstructed as maximum *z*-projections (**f**) with a total of 79 GFP^+^ and 95 tdT^+^ cells. Grey outlines indicate the organoid periphery. Scale bars, 25 µm. **g**, Average cell number per clone (D8, *n* = 109; D9, *n* = 46; D10, *n* = 93; D11, *n* = 50; D12, *n* = 61; D13, *n* = 68; D15, *n* = 40). Clone size decreased significantly over time (Kruskal–Wallis test, *P* < 0.0001). Pairwise comparisons on consecutive days showed no significance, except between D10 and D11 (multiple comparisons test, *P* = 0.0092). **h**–**j**, Illustrations (top; representative clones induced at D10 and analysed at D20 (left) and schematics (right)) of clone architectures and inferred division modes of RGPs, and quantification of cells (bottom) within the three MADM clone types (see Methods, ‘Image acquisition and analysis of MADM clones’). Average number of cells in proliferative (**h**), asymmetric neurogenic (**i**) and small neurogenic (**j**) clones induced on a single day between D8–D15 and analysed at D20. Clone size was significantly different across the period of induction for proliferative clones (**h**; D8, *n* = 53; D9, *n* = 20; D10, *n* = 46; D11, *n* = 18; D12, *n* = 17; D13, *n* = 13; generalized linear mixed model (GLMM) likelihood ratio test (LRT), *P* = 1.28 × 10^−5^), for asymmetric neurogenic clones (**i**; D8, *n* = 32; D9, *n* = 15; D10, *n* = 31; D11, *n* = 14; D12, *n* = 17; D13, *n* = 20; D15, *n* = 10; GLMM LRT, *P* = 0.018) and for small neurogenic clones (**j**; D8, *n* = 24; D9, *n* = 11; D10, *n* = 16; D11, *n* = 18; D12, *n* = 27; D13, *n* = 35; D15, *n* = 30; GLMM LRT, *P* = 0.037). **g**–**j**, Data are mean ± s.e.m. **k**, Stacked bar plot indicating the relative abundance of MADM clone architectures for each induction time point. Error bars indicate standard error of the proportion. D8, *n* = 109; D9, *n* = 46; D10, *n* = 93; D11, *n* = 50; D12, *n* = 61; D13, *n* = 68; D15, *n* = 40. *n* denotes the number of individual MADM clones (**g**–**k**). Scale bars, 50 µm. *P < 0.05, **P < 0.01, ***P < 0.001, ****P < 0.0001; NS, not significant (*P* ≥ 0.05). Source data

**Extended Data Fig. 3:**
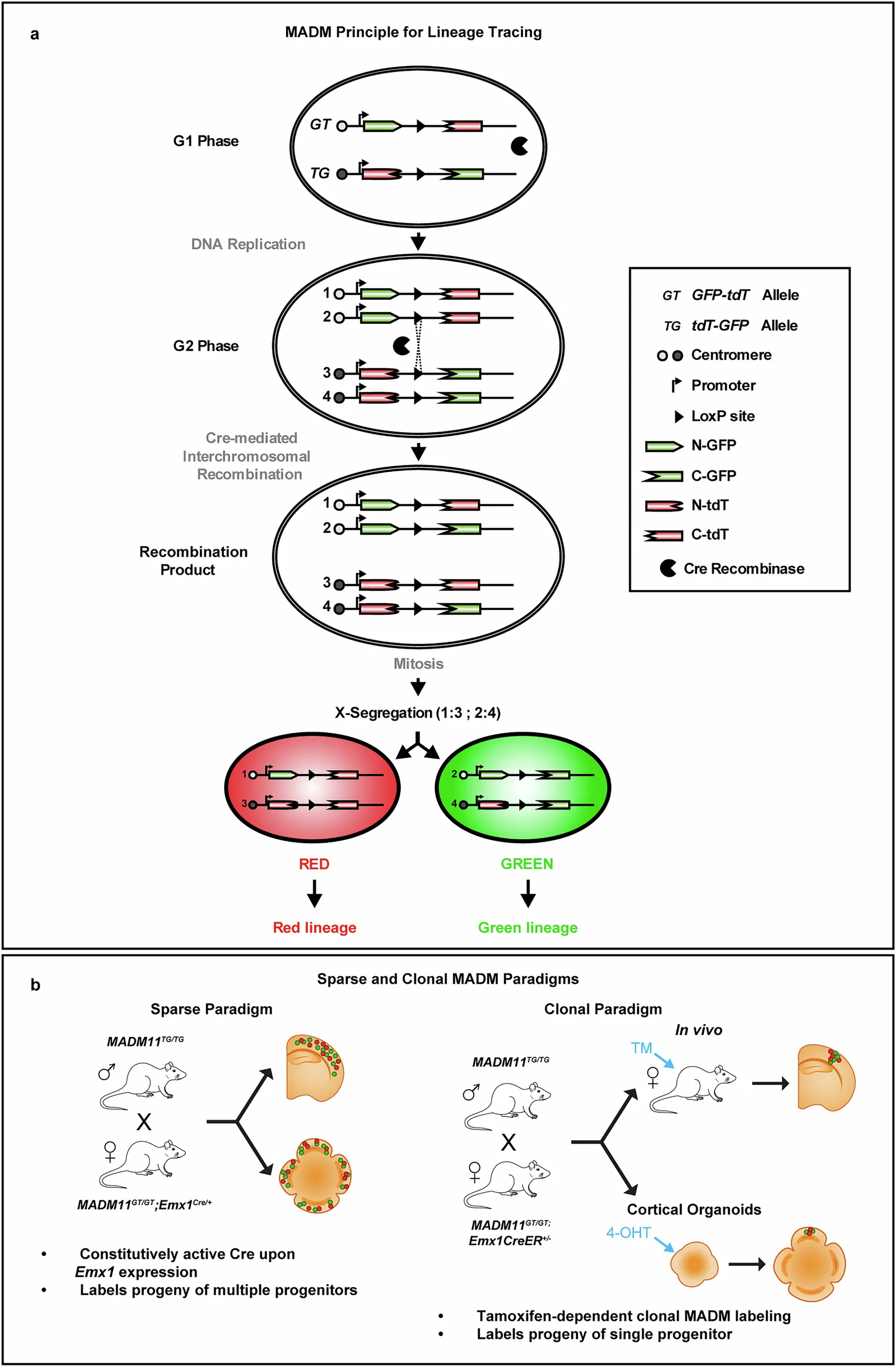
MADM Principle for Lineage Tracing and Clonal Analysis. **a**, Scheme illustrating the components and operating principle of the MADM system. MADM enables clonal analysis with single cell resolution in situ and provides an unequivocal optical readout of progenitor cell division patterns. The MADM system is composed of two split marker genes (*GT*: *GFP-tdT* and *TG*: *tdT-GFP*) containing a *loxP* site in a small intron, and inserted at identical locations on homologous chromosomes. Upon CRE-mediated interchromosomal recombination, the two marker genes (*GFP* and *tdT*) are reconstituted. If recombination occurs in the G2 phase of the cell cycle, followed by X-segregation, the two daughter cells emerging from mitosis each express one of the markers. Thus, the daughter lineages can be traced individually in situ based on the distinctive marker expression. Scheme reused and adapted with permission from^58^. **b**, Use of MADM in combination with constitutively active *Cre*-drivers results in sparse population labeling. For inducing sparse single MADM clones, TM/4-OHT-inducible *CreER*-driver can be utilized. The scheme illustrates the key advantages and use-cases for the sparse and clonal MADM paradigms, in this case with a focus on the *Emx1* lineage. (**Left**) Sparse paradigm, in vivo as well as in organoids, results in sparse labeling of the progeny derived from *Emx1*^*+*^ progenitors. *Emx1*-driven expression of constitutively active Cre recombinase catalyzes Cre-mediated interchromosomal recombination resulting in progeny of the original *Emx1*^+^ progenitor with GFP or tdT MADM labeling. (**Right**): the MADM clonal system relies on driver-dependent CreER expression. CreER requires the presence of an estrogen receptor modulator (tamoxifen [TM] or 4-hydroxy-tamoxifen[4-OHT]). In this case, the experimenter retains full temporal control of clonal labeling events. The tamoxifen, or 4-OHT, can be titrated to low levels enabling the labeling of progeny from a single progenitor in a given region or tissue of interest.

To unequivocally probe NSC lineage progression in a self-organizing cortical organoid system in situ, we established MADM technology in mESCs (Fig. 2a). MADM enables cell lineage tracing at single-cell resolution, and provides unparalleled qualitative and quantitative information about birth order of clonally related cells, progenitor cell proliferation behaviour and potential^2,3^. The MADM system is based on Cre recombinase-mediated reconstitution of two split marker genes (*Gfp* and *tdT*) in genetically defined progenitor cells undergoing mitosis^2,3^ (Extended Data Fig. 3a,b). With the use of a temporally controlled tamoxifen-inducible CreER driver, MADM events permanently mark nascent daughter cells generated by a dividing progenitor cell and their respective lineages in two distinct fluorescent colours^2,3^ (Extended Data Fig. 3a,b). MADM can thus provide an optical readout of individual progenitor division patterns with precise temporal accuracy.

**Extended Data Fig. 4:**
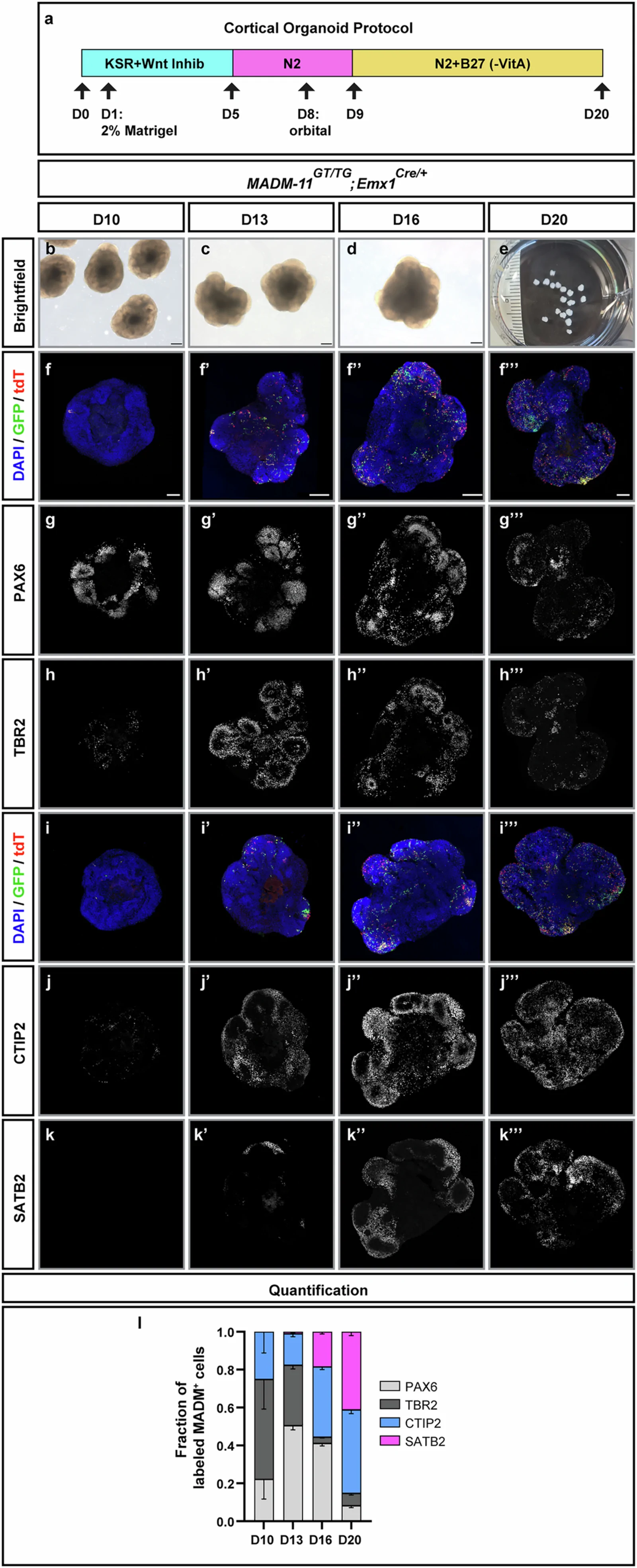
Generation of *MADM-11*^*GT/TG*^;*Emx1*^Cre/+^ cortical organoids from mESCs and cortical marker expression. **a**, Scheme depicting an overview of the protocol for cortical organoid generation. (Blue) mESCs were plated at day 0 (D0) in the presence of knockout serum (KSR) and a Wnt inhibitor to generate embryoid bodies, then transferred at D1 to the same medium with addition of 2% growth-factor-reduced Matrigel. (Pink) From D5-D9, organoids were maintained in a medium containing N2 supplement, and placed on an orbital shaker from D8. From D9, the culture medium contained both N2 and B27 (without vitamin A) supplements. Organoids were collected at D20. **b-e**, Bright field images of whole cortical organoids at D10 (**b**), D13 (**c**), D16 (**d**), and D20 (**e**). **f-k**, Representative sections of organoids derived from *MADM-11*^*GT/TG*^;*Emx1*^*Cre/+*^ mESCs with population MADM labeling in *Emx1*^*+*^ cortical lineage at D10 (**f-k**), D13 (**f’-k’**), D16 (**f”-k”**), and D20 (**f”’-k”’**). Sections with MADM labeling were immunostained for GFP (green), tdT (red) and DAPI (blue, to visualize nuclei) (**f and i**); and selected cell markers (white) including PAX6 (**g**, RGPs), TBR2 (**h**, IPs), CTIP2 (**j**, SCPNs), and SATB2 (**k**, CPNs). **l**, Quantification of the fraction of MADM^+^ cells that were double-positive for selected markers PAX6 (grey), TBR2 (dark grey), CTIP2 (blue), and SATB2 (magenta) across 4 time points (D10, D13, D16, and D20). Bars show mean and 95% confidence interval. D10: n = 5 organoids, D13: n = 5 organoids, D16: n = 5 organoids, D20: n = 5 organoids. Fisher’s exact test (two-tailed with Bonferroni multiple test correction): D10 vs D13 *p* = 0.0199 **, D13 vs D16 *p* ≤ 0.0001 ****, D16 vs D25 *p* ≤ 0.0001 ****. For statistical analysis by cell marker, see Source Data. Nuclei were stained using DAPI (blue). Scale bar: 200 µm (**b-d**), 100 µm (D10-D16 sections), 200 µm (D20 sections). Source data

**Extended Data Fig. 5:**
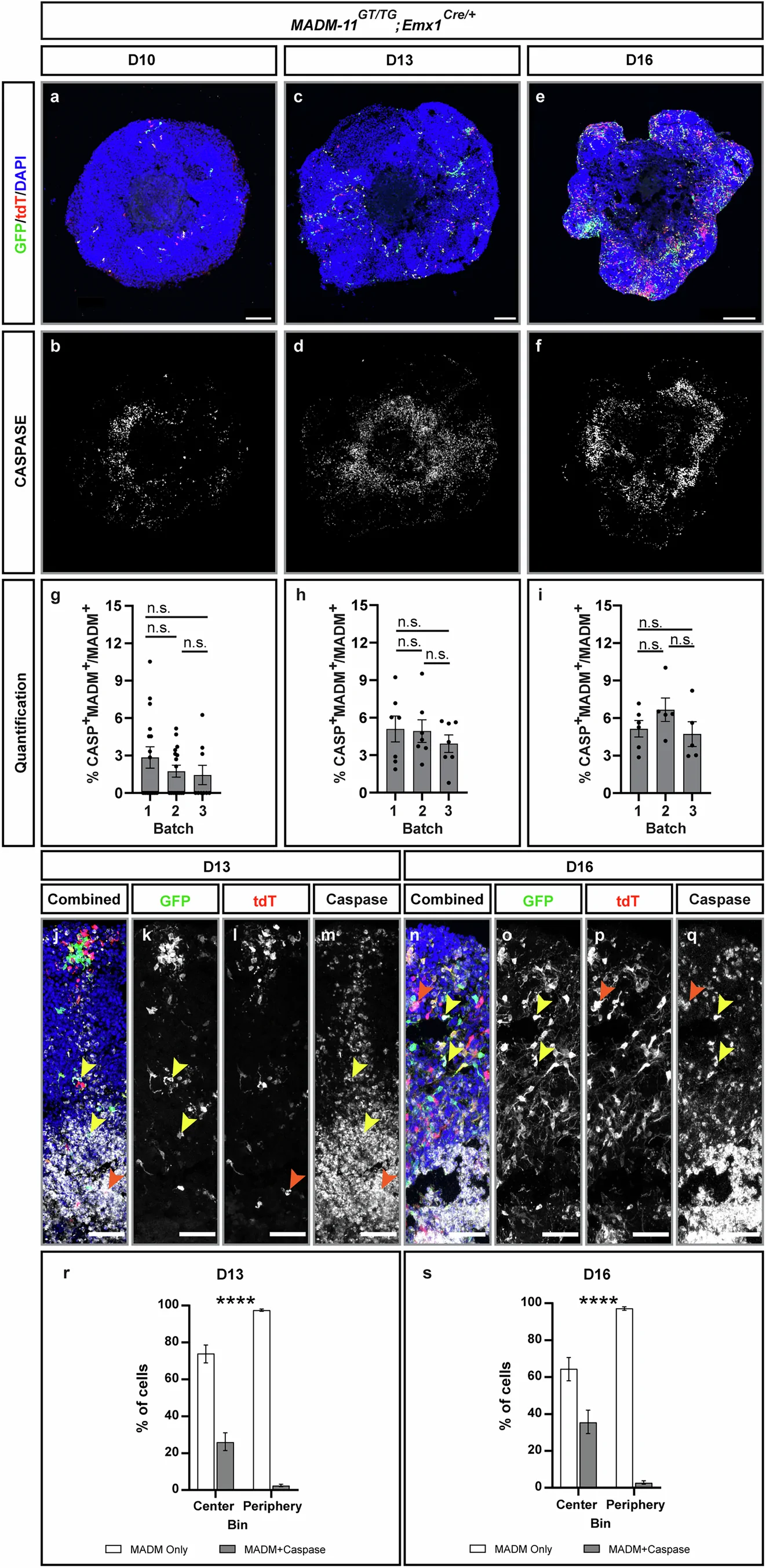
Analysis of cell death in cortical organoids. **a-f**, Representative images of *MADM-11*^*GT/TG*^;*Emx1*^*Cre/+*^ organoid sections, immunostained for MADM labeling (**a**, **c**, **e**,; GFP^+^, green and tdT^+^, red), and CASPASE-3 (**b**, **d**, **f**,; white) at D10 (**a** and **b**), D13 (**c** and **d**), and D16 (**e** and **f**). **g-i**, Quantification of MADM^+^/CASPASE-3^+^ double positive cells for three batches at D10 (**g**, Batches 1 and 2 n = 16 organoids per batch, Batch 3 n = 9), D13 (**h**, n = 7 organoids per batch), and D16 (**i**, Batch 1 n = 6, Batches 2 and 3 n = 5 organoids per batch). Data represent mean ± S.E.M. Note that % of MADM^+^/CASPASE-3^+^ double positive cells was similar to the range of cell death observed in vivo^1^. **j-m** and **n-q**, Representative images at higher magnification indicating *MADM-11*^*GT/TG*^;*Emx1*^*Cre/+*^ organoid sections, immunostained for MADM labeling (**j-l** and **n-p**; GFP^+^, green and tdT^+^, red) and CASPASE-3 (**j**, **m**, **n**, **q**, white) at D13 (**j-m**), D16 (**n-q**). **r-s**, Quantification of MADM^+^ (white bars) and MADM^+^/CASPASE-3^+^ double positive (grey bars) cells in either the periphery or center of each organoid section at D13 (**r**) and D16 (**s**), respectively. The periphery was defined as the outer 250-300 µm of tissue, depending on the overall size of the organoid, and the center encompassed the tissue beyond the periphery. Error bars indicate 95% confidence interval. D13 n = 7 sections, D16 n = 5 sections. Fisher’s exact test (two-tailed): D13 Center vs Periphery *p* ≤ 0.0001 ****, D16 Center vs Periphery *p* ≤ 0.0001 ****. Nuclei were stained using DAPI (blue). Scale bars: 100 µm (**a**-**d**), 200 µm (**e** and **f**), 25 µm (**j-q**). Source data

To exploit the MADM paradigm in the cortical organoid system, we generated novel mESC lines (Supplementary Fig. 6) and first analysed MADM labelling at the population level (*MADM-11*^*GT/TG*^;*Emx1*^*cre/+*^; Extended Data Fig. 3b) in combination with specific markers. We observed an overall decrease in progenitors (PAX6^+^ RGPs and TBR2^+^ intermediate progenitors) as organoids developed, with a concomitant increase in postmitotic neurons (CTIP2^+^ and SATB2^+^) (Extended Data Fig. 4). To assess cell death, we performed caspase-3 immunostaining and observed that the overall proportion of MADM^+^caspase-3^+^ cells as a percentage of MADM^+^ cells was below 6% (on average across batches and differentiation time points), which was in a similar range to naturally occurring cell death rates in vivo^1,27^ (Extended Data Fig. 5a–i). We also showed that cell death was largely restricted to the core of the organoids (Extended Data Fig. 5j–s), in agreement with previous findings in human organoids^28^.

To conduct MADM-based lineage tracing at the individual RGP level (Fig. 2a), we combined MADM reporter cassettes with the temporally inducible *Emx1-creER* driver (*MADM-11*^*GT/TG*^;*Emx1-creER*^*+/−*^). Previous efforts using such a genetic paradigm and utilizing progressive MADM clonal interval sampling in vivo have led to an inaugural quantitative framework of RGP lineage progression^1^, which will serve as a blueprint for subsequent analysis herein (Fig. 2a, top right). We therefore crossed the same MADM reporter-CreER driver mice as previously used for in vivo analysis, but instead of growing embryos to term, we isolated blastocysts and derived *MADM-11*^*GT/TG*^;*Emx1-creER*^*+/−*^ mESC lines (Supplementary Fig. 7) for subsequent cortical organoid generation.

## Clonal analysis in cortical organoids

Given that MADM mESCs have the identical genotype as in our previous in vivo experiments, we next pursued MADM-based clonal analysis with the use of 4-hydroxytamoxifen (4-OHT) and assessed RGP lineage progression in a self-organizing cortical organoid system. We conceived a systematic MADM clone induction paradigm (Fig. 2b) with the goal of obtaining around one cluster of red and green MADM-labelled cells per organoid. Individual MADM clones were digitally reconstructed for quantification^29^ (Fig. 2c–f). We induced MADM clones starting at D8 and up to D15 with analysis at D20. The average MADM clone size appeared smaller than in vivo (up to twofold even at the earliest differentiation stage) but decreased significantly over time, consistent with diminishing progenitor potential. However, pairwise comparisons of average clone size between consecutive clone induction time points were rarely significant in cortical organoids (Fig. 2g), unlike in vivo, where clonal output decreased exponentially and significantly between corresponding developmental time points^1^.

**Extended Data Fig. 6:**
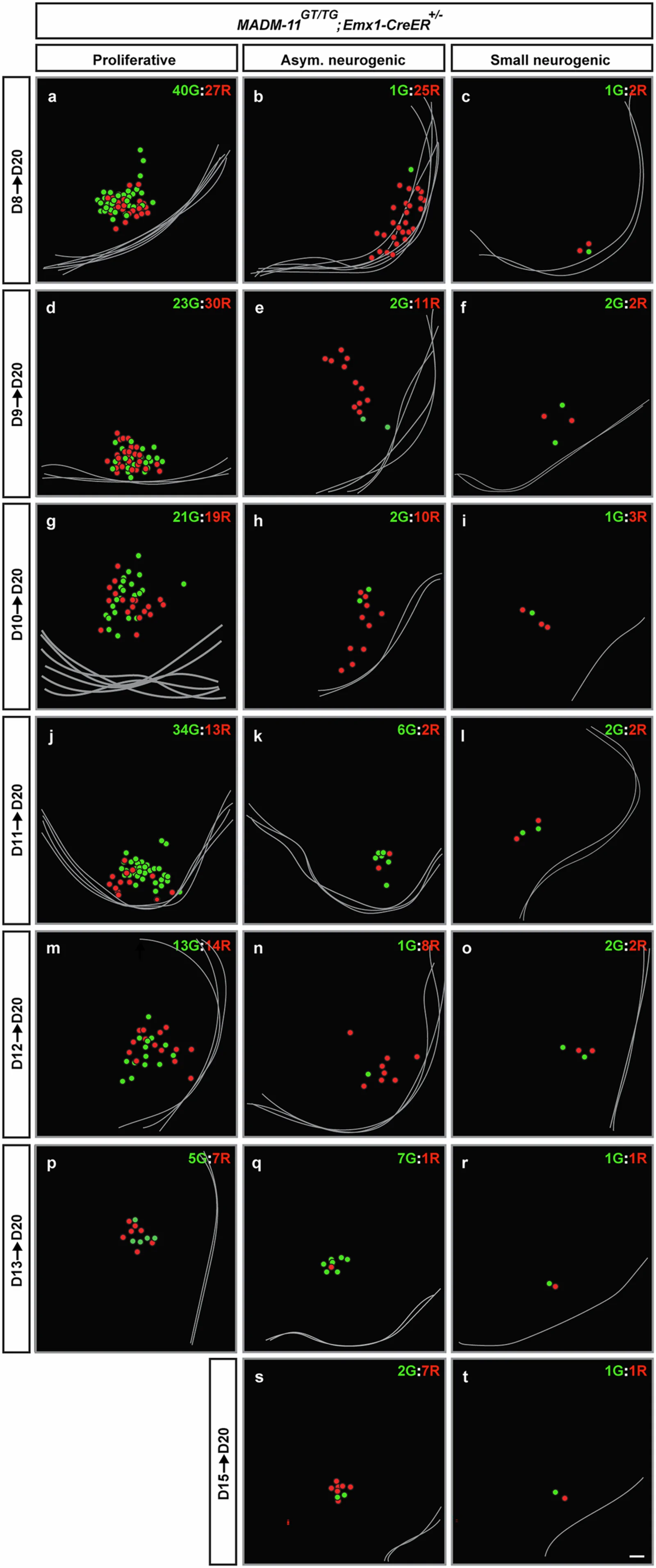
Reconstructions of MADM clone architectures in organoids. **a-t**, Reconstructions of representative proliferative (**a**, **d**, **g**, **j**, **m**, and **p**), asymmetric neurogenic (**b**, **e**, **h**, **k**, **n**, **q** and **s**), and small neurogenic (**c**, **f**, **i**, **l**, **o**, **r** and **t**) MADM clones in organoids derived from *MADM-11*^*GT/TG*^;*Emx1-CreER*^*+/−*^ mESCs; induced at D8 (**a**-**c**), D9 (**d**-**f**), D10 (**g**-**i**), D11 (**j**-**l**), D12 (**m**-**o**), D13 (**p**-**r**) and D15 (**s** and **t**) with analysis at D20. Each clone is presented as a maximum z-projected cluster of red (tdT) and green (GFP) cells. Gray outlines indicate the organoid periphery. For illustration purposes, all clones were oriented in the same direction. Scale bar: 50 µm.

Previous work has demonstrated that RGP proliferation behaviour correlates with lineage progression, whereby at early developmental stages, RGPs exclusively divide symmetrically to amplify their pool (proliferative division), followed by a self-renewing asymmetric neurogenic division mode^1^. Therefore, we next analysed MADM clones to infer progenitor division mode (Fig. 2h–j and Extended Data Fig. 6) in cortical organoids by applying the same MADM-defined clone classes, which remain applicable despite incomplete layering, as in previous in vivo experiments^1^. Whereas we observed both proliferative and asymmetric neurogenic division modes, we also noted a sizeable fraction of clones that did not fall into any of the above categories, which we termed ‘small neurogenic’.

**Extended Data Fig. 7:**
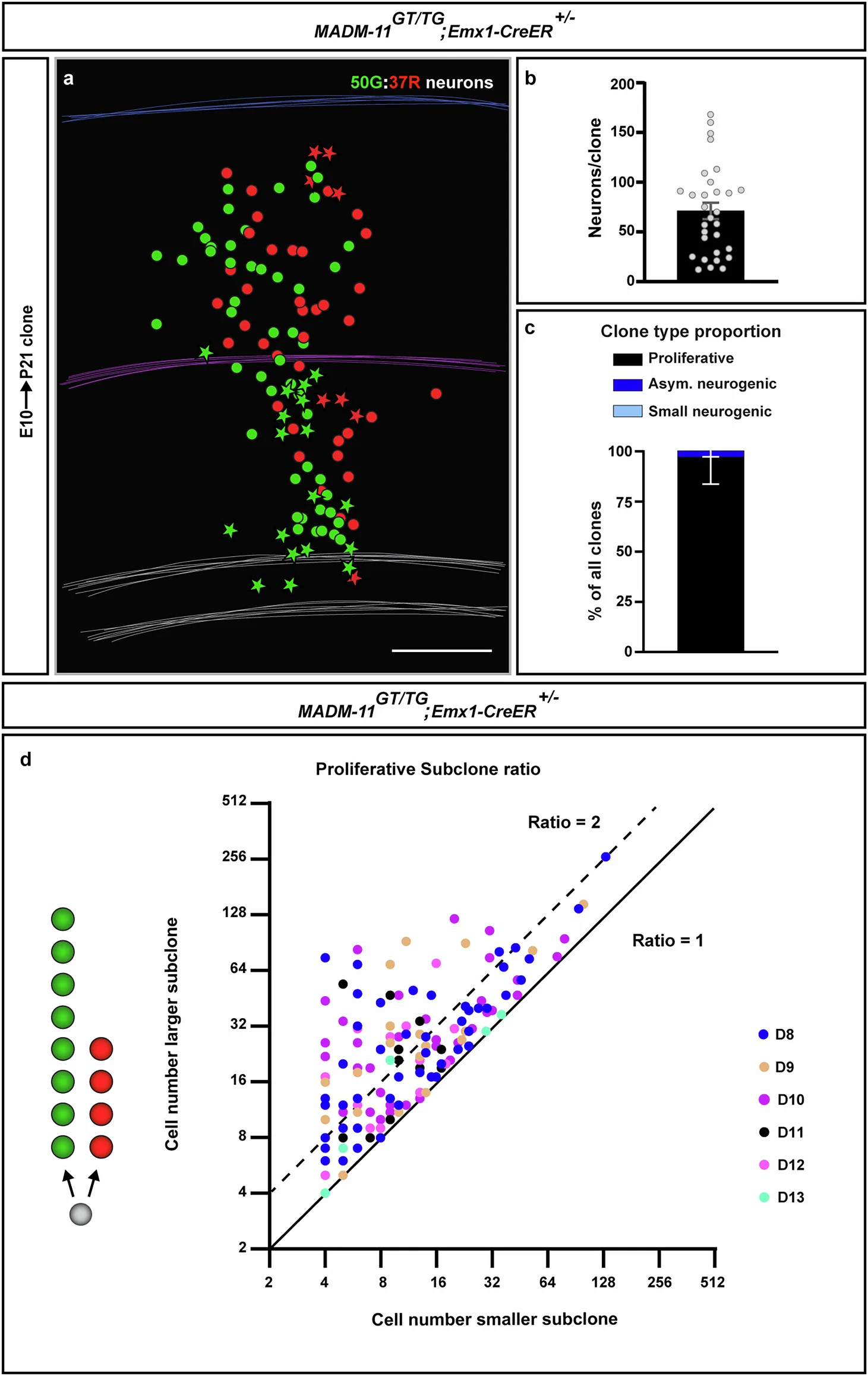
Proportions of different clone architectures in E10-P21 MADM clones in vivo and quantitative assessment of proliferative MADM subclone size in organoids. **a**, Representative MADM clone in somatosensory cortex, induced at E10 and analyzed at P21 in *MADM-11*^*GT/TG*^;*Emx1-CreER*^*+/−*^ mice. Clone is presented as a maximum z-projected cluster of red (tdT) and green (GFP) cells shown as dots (neurons) and stars (glia). Colored lines indicate pia, deep/upper layer boundary, and white matter. **b**, Quantification of neuron numbers per MADM clone (n = 30 MADM clones). Data represent mean ± S.E.M. **c**, Stacked bar plot indicating the proportion of proliferative (29/30 = 96.7%), asymmetric (1/30 = 3.3%) and small (0/31) neurogenic E10-P21 MADM clones. Note that very small proportion (i.e. 3.3%) show asymmetric neurogenic architecture; and the absence of small neurogenic clone architecture in clones induced at early E10 developmental time. Error bars indicate 95% confidence interval. n = 30 clones. Scale bar: 200 µm. **d**, For each proliferative clone, the cell number of the smaller and larger subclone were plotted on the x-axis and y-axis, respectively, for each MADM clone induction time point (D8-D13). Subclone size ratios of 1 and 2 are indicated by the solid and dashed lines. Note that in contrast to in vivo condition^1^, the ratios of many clones were larger than two (~1.6 on average in vivo). Source data

**Extended Data Fig. 8:**
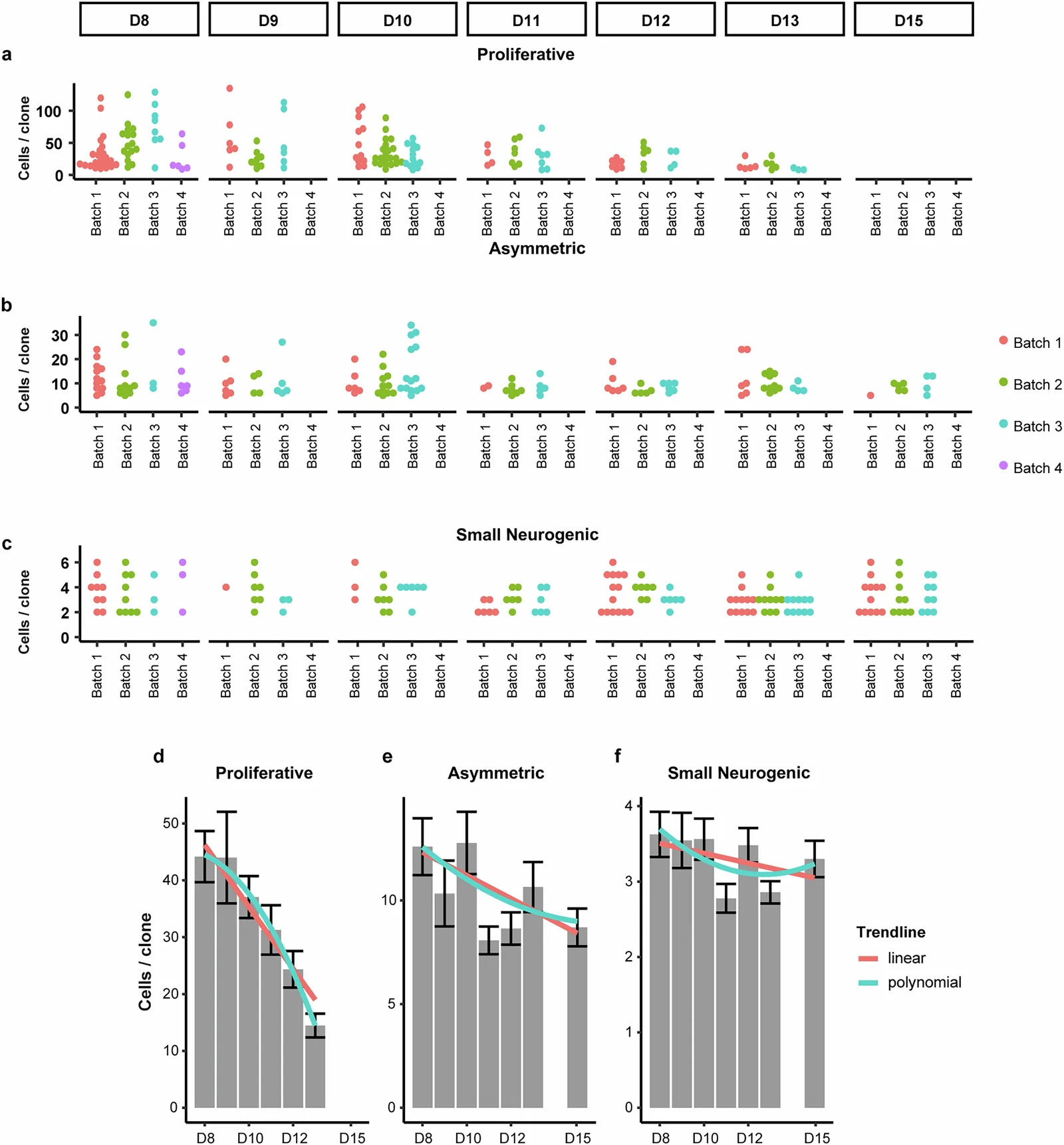
Quantification of cell numbers and trendline analysis in MADM clones with distinct clone architecture across different batches. **a-c**, Quantification of cell number per MADM clone in proliferative (**a**), asymmetric (**b**) and small neurogenic clones (**c**) with distinct induction time points (D8-D15) across four different batches of organoids for D8 induction and 3 different batches for D9-D15 induction time points. Note that statistical tests identified no major batch effects, except for batch 3 (1/4 batches) at induction time point D8 See Source Data for detailed analysis. **d-f**, Linear and polynomial trendline analysis of clone size in proliferative (**a**), asymmetric (**b**) and small neurogenic MADM clones (**c**) with distinct induction time points (D8, D10, D12, and D15). Data shown as mean ± S.E.M. This analysis is an extension of that in Fig. 2**g-k**. Proliferative clones (**d**, D8, n = 53; D9, n = 20; D10, n = 46; D11, n = 18; D12, n = 17; D13, n = 13), asymmetric neurogenic (**e**, D8, n = 32; D9, n = 15; D10, n = 31; D11, n = 14; D12, n = 17; D13, n = 20; D15, n = 10), and small neurogenic clones (**f**, D8, n = 24; D9, n = 11; D10, n = 16; D11, n = 18; D12, n = 27; D13, n = 35; D15, n = 30). Source data

Next, we quantified progenitor potential and output by taking into account progenitor cell division mode. Proliferative clone size in organoids decreased gradually over time from 44.17 ± 4.5 at D8 to 14.46 ± 2.1 at D13 (Fig. 2h), and was almost half the size on average at D10 (37.07 ± 3.69 cells per clone) when compared to proliferative clones in vivo at E10 (71.2 ± 8.3 cells per clone^1^ (Extended Data Fig. 7a–c); note that E10 and D10 are comparable transcriptionally (Extended Data Fig. 1e)). The output potential of the two sister RGPs emerging from a proliferative mother RGP in vivo was shown to be very similar (that is, mean ratio of larger/smaller sister subclone in the range of about 1.6) across time and thus within the full spectrum of clone sizes. However, in cortical organoids, sister RGPs showed less correlated proliferation potential, especially at early differentiation time points (Extended Data Fig. 7d). Despite a progressive decrease in asymmetric clone size and an absence of small neurogenic clones in vivo during the neurogenic phase (E12–E16), the reductions in size of asymmetric and small neurogenic clones in organoids were less pronounced over time (Fig. 2i,j and Extended Data Fig. 8).

Next, we quantified the abundance of all three MADM clone architectures (inferred progenitor division mode) for each clone induction time point (Fig. 2k). Of note, all three progenitor division modes occurred concurrently at every clone induction time point. Proliferative divisions persisted until late differentiation stages; asymmetric neurogenic divisions did not become predominant, but instead small neurogenic clone architectures increased over time. Although smaller clone sizes also appeared at later developmental stages in vivo owing to decreasing progenitor potential^1,30^, small neurogenic clones were not detected when MADM events were induced at earlier developmental stages such as E10, a stage at which RGPs were found almost exclusively (96.7%) in symmetric proliferative division mode in vivo^1^ (Extended Data Fig. 7a–c).

**Extended Data Fig. 9:**
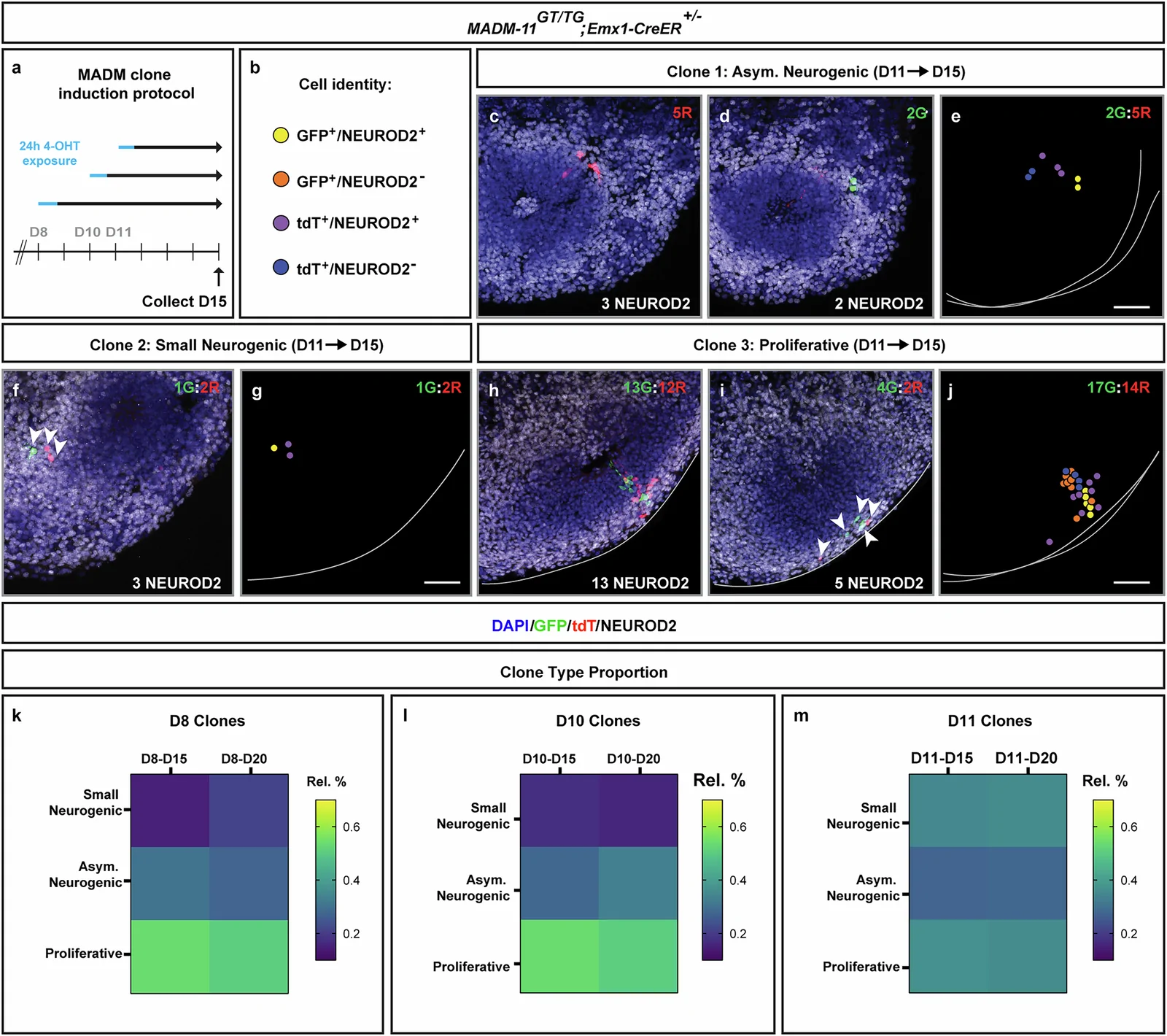
Proportions of different MADM clone architectures at D15 differentiation time point. **a**, MADM clone induction protocol in *MADM-11*^*GT/TG*^;*Emx1-CreER*^*+/−*^ cortical organoids. MADM clones were induced at different developmental time points (D8, D10, D11) by 24 h 4-OHT exposure, with analysis at D15. **b**, Color scheme representing cell-type identity: yellow (GFP^+^/NEUROD2^+^), orange (GFP^+^/NEUROD2^*−*^), purple (tdT^+^/NEUROD2^+^), blue (tdT^+^/NEUROD2^*−*^). NEUROD2^+^ cells represent nascent postmitotic neurons and NEUROD2^*−*^ indicate progenitors. **c-j**, Serial sections depicting representative MADM clones, induced at D11 and analyzed at D15, and indicating asymmetric neurogenic (**c-e**), small neurogenic (**f** and **g**), and proliferative (**h-j**) clone architectures. Immunostaining indicates GFP^+^ (green), tdT^+^ (red), and NEUROD2^+^ (white) cells. **k-m**, Heatmaps illustrating quantification of relative abundance (%) of MADM clone architectures (proliferative, asymmetric neurogenic, small neurogenic) at D15, induced at D8 (**k**, n = 39 clones), D10 (**l**, n = 28 clones), and D11 (**m**, n = 54 clones), compared to D20. No statistically significant differences were found between D15 and D20 (Fisher’s exact test, see Source Data for analysis details). DAPI staining (blue) indicates nuclei. Scale bar: 50 µm. Source data

The presence of high numbers of small neurogenic clones, even for clones induced at early differentiation stages, could reflect false positives, in the sense that an increased rate of cell death over time would ‘erode’ original large proliferative clones to small sizes, which would thus ultimately appear as small neurogenic clones. To test this possibility, we assessed MADM clones 5 days earlier (that is, at D15 rather than at D20) and determined their relative abundance compared to D20 (Extended Data Fig. 9). We utilized NEUROD2 (a marker for postmitotic nascent projection neurons) immunostaining and reasoned that (1) a small neurogenic clone would contain only NEUROD2^+^ cells; (2) in an asymmetric neurogenic clone the smaller subclone would contain only NEUROD2^+^ cells; and (3) a proliferative clone would contain NEUROD2^−^ cells in both subclones. We induced clones at D8, D10 and D11 with analysis at D15, but found similar relative proportions of clone architectures for all three induction time points when compared to the data obtained at D20 (Extended Data Fig. 9k–m). Thus, cell death seems to not be a major factor influencing the interpretation of our data, although we cannot exclude the possibility that sparse, sporadic cell death may eliminate some cells. Nevertheless, small neurogenic clones represent an organoid-specific clone type that is not observed in vivo.

**Extended Data Fig. 10:**
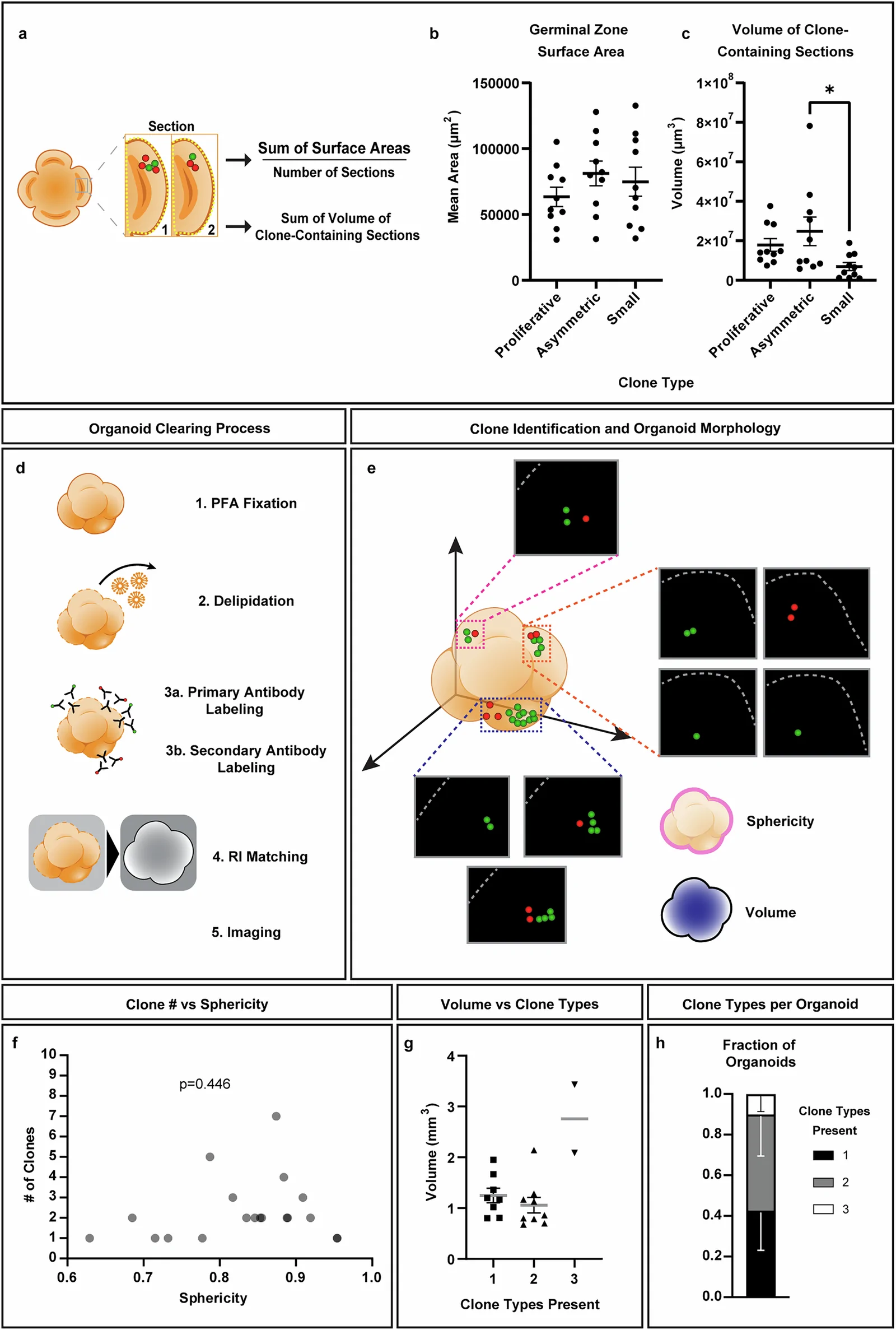
Morphological analyses of organoids containing MADM clones. **a**, Scheme indicating the methodological approach. **b**, The average surface area of rosettes did not correlate with a particular clone type. One-way ANOVA with Tukey’s multiple comparisons test. **c**, Small neurogenic clones occupied significantly less space throughout the rosette than asymmetric neurogenic clones. One-way ANOVA with Tukey’s multiple comparisons test: Symmetric vs Asymmetric *p* = 0.56, Symmetric vs Small *p* = 0.25, Asymmetric vs Small *p* = 0.0327 *. (**b-c**) Data shown as mean ± S.E.M. n = 30 clones. **d**, Scheme indicating the methodological approach of clearing, labeling, RI (refractive index) matching, and imaging whole mouse cortical organoids. **e**, Illustration and schematic of one representative organoid. This large organoid contained 3 identifiable clones. The two key morphological features – sphericity and volume – were quantified. **f**, Organoid sphericity did not correlate with the number of clones within an organoid. Pearson correlation test (two-tailed) *p* = 0.446. **g**, Clone types (proliferative, asymmetric neurogenic, or small neurogenic) did not correlate with organoid volume. Note however that the two organoids which contained all 3 clone types tended to be larger than all others. Data shown as mean ± S.E.M. **h**, Stacked bar plot indicating the fraction of distinct clone types present within an organoid (57.9% of organoids contained 2 or more clone types, 42.1% contained only 1 type of clone, in rare cases all 3 clone types were present). Error bars indicate 95% confidence interval. (**c-e**) n = 19 organoids. Source data

We found that rosette size did not correlate with abundance of any clone type, but small neurogenic clones had a significantly smaller spread across sections compared with asymmetric neurogenic clones (Extended Data Fig. 10a–c). Using optical clearing methods, we observed that gross organoid morphology did not correlate with specific abundance of any of the three clone types (Extended Data Fig. 10d–h).

**Extended Data Fig. 11:**
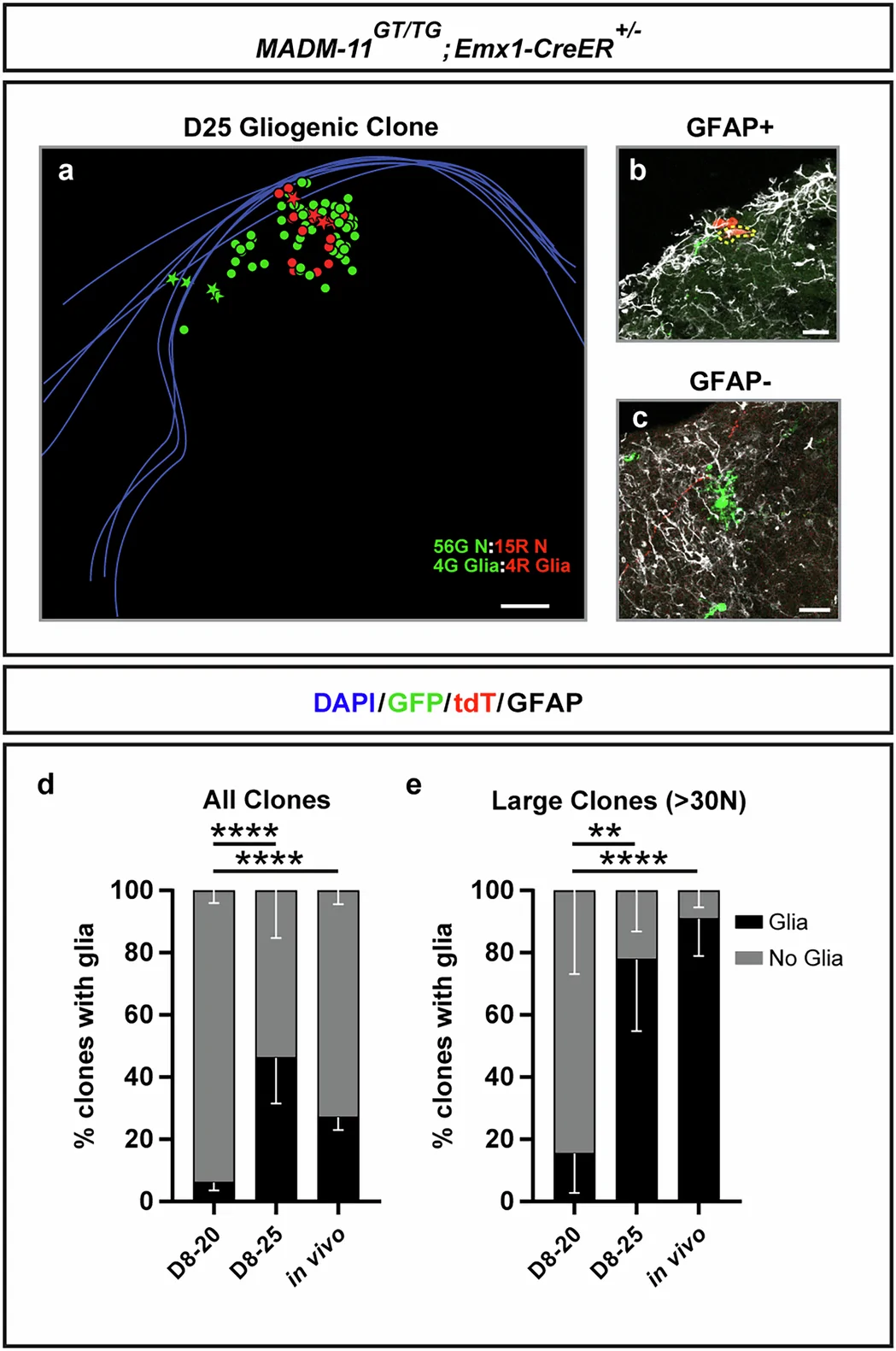
RGPs in cortical organoids sequentially adopt gliogenic potential. **a**, Reconstruction of a representative glia-containing MADM clone. **b-c**, Representative images of a GFAP^+^ (**b**) and a GFAP^*−*^ (**c**) glial cell based on morphological assessment. **d-e**, Quantification of the percent of all clones (**d**) and clones with >30 neurons (**e**) containing glia. D8-D20: n = 215 clones; D8-D25: n = 39 clones, in vivo n = 410 clones^1^. Fisher’s exact test (two-tailed): (**d**) D8-20 vs D8-25 *p* = 1.46e-8 ****, D8-20 vs in vivo *p* = 1.44e-10 ****, D8-25 vs in vivo *p* = 0.095; (**e**) D8-20 vs D8-25 *p* = 0.0057 **, D8-20 vs in vivo *p* = 2.92729e-6 ****, D8-25 vs in vivo *p* = 1.0. Error bars indicate 95% confidence interval. Source data

Organoids were competent to switch from neurogenesis to gliogenesis. Whereas at D20 organoids contained very few glia, around 46% of all clones contained glia at D25, and a large fraction of clones with more than 30 neurons contained glia at D25, similar to the in vivo paradigm (Extended Data Fig. 11). Together, the above findings in self-organizing cortical organoids contrast with the in vivo concept of linear, temporally stereotyped progression of RGP cell division mode from strictly proliferative to exclusively asymmetric neurogenic, while still sequentially executing the switch from neurogenic to gliogenic.

## RGP lineage restriction in organoids

**Fig. 3:**
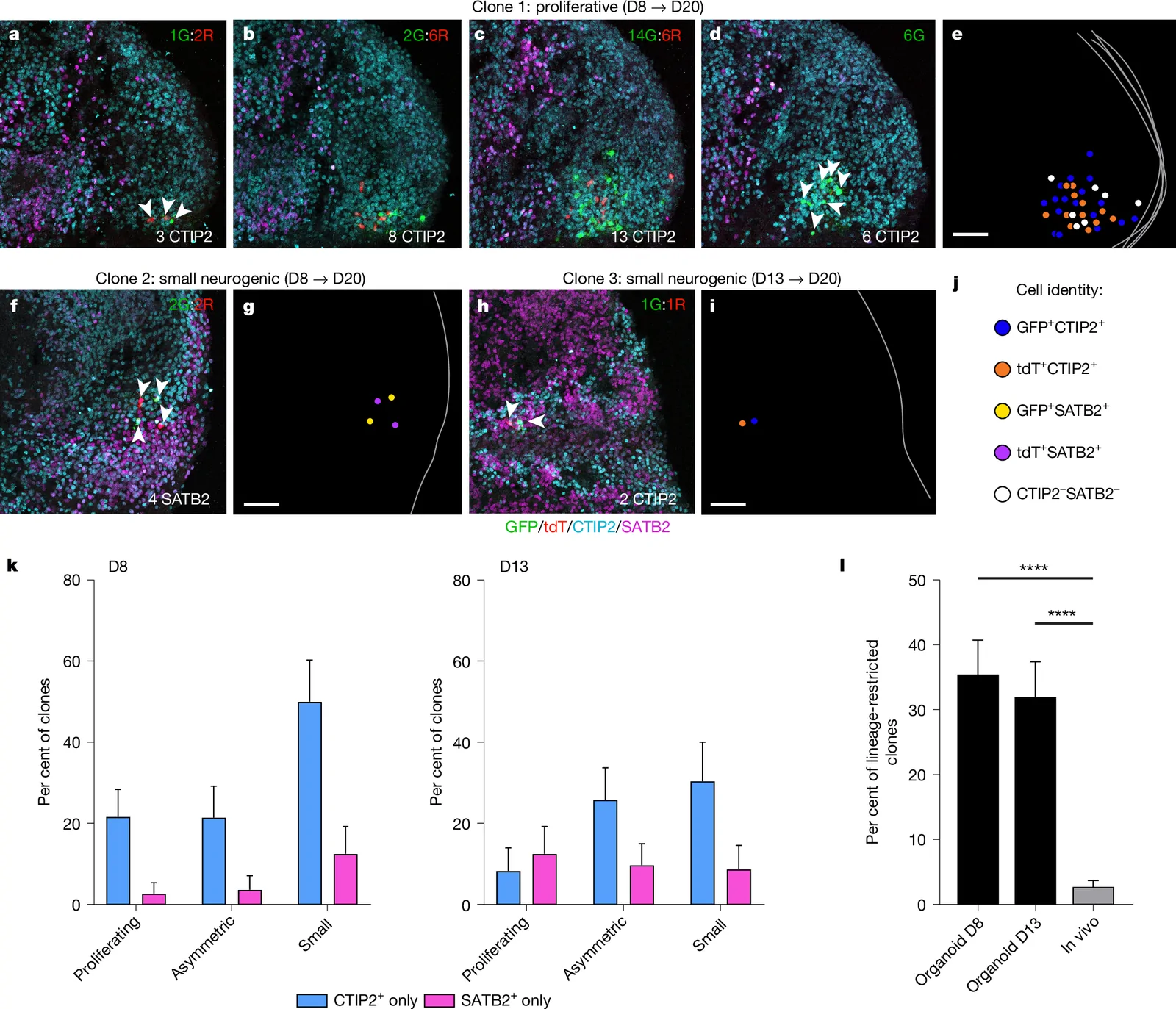
RGPs show a high level of lineage restriction in organoid system. **a**–**e**, A representative proliferative MADM clone, induced at D8 and analysed at D20, with lineage restriction towards CTIP2^+^ cells. The clone is depicted in consecutive sections immunostained for GFP, tdT, CTIP2 and SATB2 (**a**–**d**) and as a reconstruction (**e**). The clone contained 37 cells (30 CTIP2^+^, 0 SATB2^+^ and 7 SATB2^−^CTIP2^−^ cells). **f**,**g**, A representative small neurogenic clone, induced at D8 and analysed at D20, with lineage restriction towards SATB2^+^ cells. The clone is depicted immunostained for GFP, tdT, CTIP2 and SATB2 (**f**) and as a reconstruction (**g**). The clone contained 4 cells (4 SATB2^+^ and 0 CTIP2^+^ cells). Note that D8 corresponds to an early differentiation time point whereby RGPs at a comparable developmental stage in vivo mostly produce CTIP2^+^ neurons. **h**,**i**, A representative small neurogenic clone, induced at D13 and analysed at D20, with lineage restriction towards CTIP2^+^ cells. The clone is depicted immunostained for GFP, tdT, CTIP2 and SATB2 (**h**) and as a reconstruction (**i**). The clone contained 2 cells (0 SATB2^+^ and 2 CTIP2^+^ cells). Note that D13 corresponds to a late developmental stage, in which RGPs in vivo mostly produce SATB2^+^. **j**, Colour scheme indicating cell-type identities in **e**,**g**,**i**. **k**, Bar plot indicating the percentage of proliferative, asymmetric neurogenic and small neurogenic clones with lineage restriction towards CTIP2^+^ or SATB2^+^, induced at D8 (left; proliferative, *n* = 37; asymmetric, *n* = 28; small, *n* = 22) or D13 (right; proliferative, *n* = 24; asymmetric, *n* = 31; small, *n* = 23) and analysed at D20. Error bars indicate standard error of the proportion. **l**, Percentage of total lineage-restricted MADM clones induced at D8 (*n* = 87; 31 out of 87 clones, 35.6%) and D13 (*n* = 78; 25 out of 78 clones, 32.1%) in organoids, and in vivo (*n* = 386; 11 out of 386 clones, 2.85%; see Methods, ‘Laminar positioning in MADM clones’). Data are mean + standard error of the proportion. Chi-square test: D8 versus in vivo *P* ≤ 0.0001; D13 versus in vivo *P* ≤ 0.0001. *n* denotes the number of individual MADM clones (**k**,**l**). Scale bars, 50 µm. Source data

Individual RGPs in vivo are multipotent and generate both CTIP2^+^ subcerebral projection neurons (SCPNs) and SATB2^+^ callosal projection neurons (CPNs) in a temporally sequential manner, with SCPNs produced predominantly at early neurogenic stages and CPNs produced at later neurogenic stages^7,26,31^. To assess RGP multipotency in cortical organoids, we induced MADM clones at D8 (early) and D13 (late) differentiation stages, and monitored the presence of clonally related CTIP2^+^ and SATB2^+^ cells within individual MADM clones (Fig. 3a–j). We found a large number of MADM clones that contained CTIP2^+^ SCPNs but no SATB2^+^ CPNs, and conversely, a substantial number of clones that contained SATB2^+^ CPNs but no CTIP2^+^ SCPNs. Lineage restriction was found in MADM clones induced at D8 (35.6% of clones) and D13 (32.1% of clones), and among all three types of clone architecture (that is, proliferative, asymmetric neurogenic and small neurogenic), regardless of batch (Fig. 3k and Supplementary Fig. 8). When compared to the in vivo condition, in which around 3% of RGP clones showed some bias towards either SCPN or CPN^1,9^, lineage restriction in the cortical organoid system was increased by an order of magnitude (Fig. 3l).

**Fig. 4:**
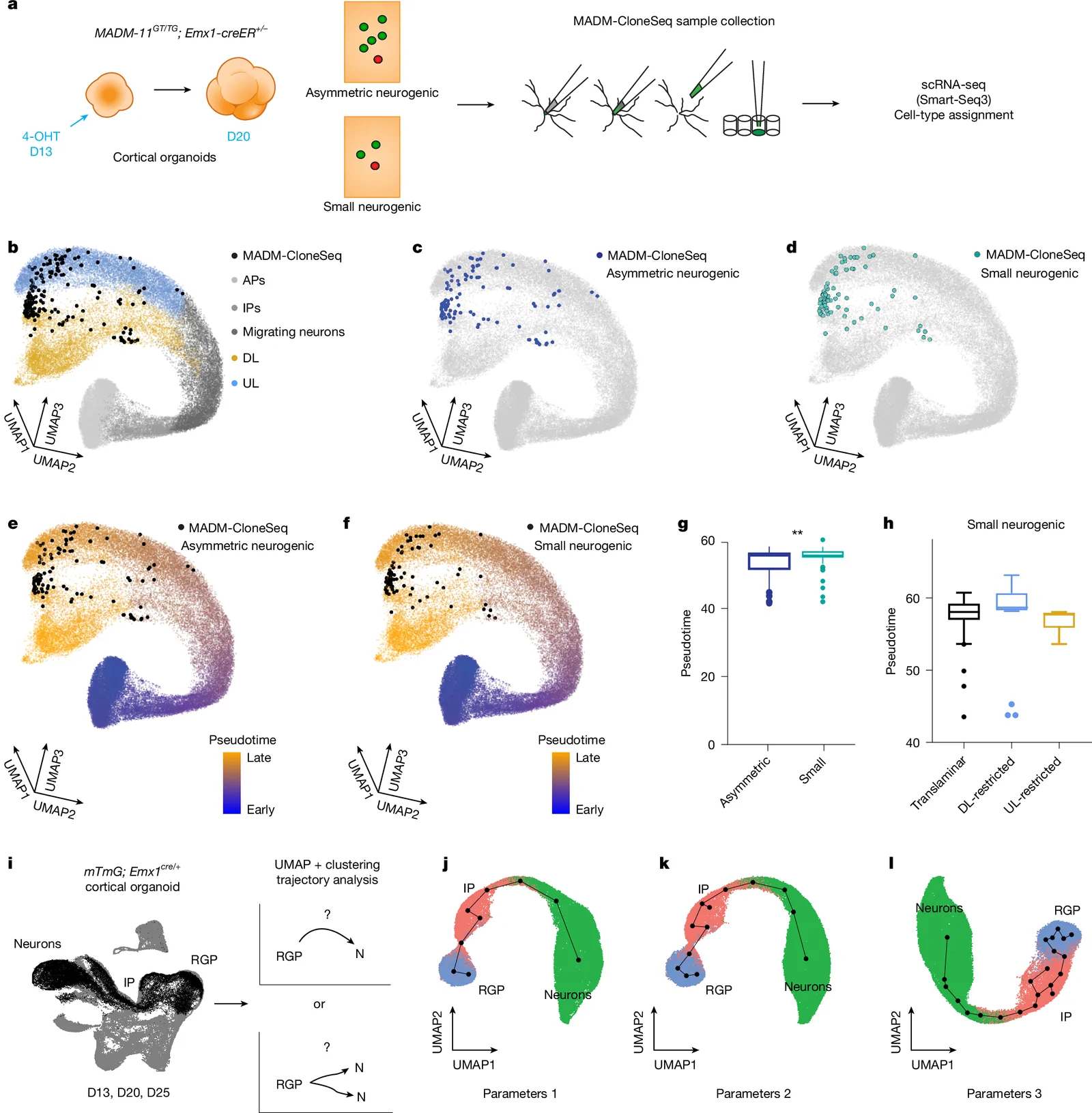
MADM-CloneSeq in combination with scRNA-seq reveals a single developmental trajectory of RGP lineage progression to generate all neuronal cell types in a cortical organoid system. **a**, Schematic illustrating the MADM-CloneSeq experimental workflow in cortical organoids containing asymmetric neurogenic and small neurogenic clones induced at D13 and collected at D20. Cell collection scheme adapted from ref. ^32^, CC BY 4.0 (http://creativecommons.org/licenses/by/4.0/). **b**–**d**, Projection of 195 MADM-CloneSeq cells on a reference UMAP showing all cells (**b**), neurons from asymmetric neurogenic clones (**c**) and neurons from small neurogenic clones (**d**), in the context of a reference cell-type atlas^26^. APs, apical progenitors. **e**,**f**, UMAPs as depicted in **c**,**d**, but with reference cells coloured on the basis of pseudotime analysis. **g**, Box plots comparing pseudotime distribution of neurons from asymmetric (asym.) and small neurogenic clones (Welch’s two-sample *t*-test *P* value = 0.005, two-tailed). Note, although the variation of pseudotime within the small neurogenic clone cells was significantly smaller than in asymmetric neurogenic clone cells (asymmetric, 4.91; small, 1.56), resulting in a significant difference, the absolute difference in pseudotime was still small. These data indicate that small neurogenic clones are likely to be generated within a smaller temporal window. Asymmetric, *n* = 109 cells; small, *n* = 86 cells. **h**, Box plots comparing pseudotime distribution of neurons from DL-restricted, UL-restricted or translaminar small neurogenic clones (two-way ANOVA with Tukey’s honest significant difference *P* value = 0.9). **g**,**h**, The centre line indicates the median, box limits show the 25–75% interquartile range (IQR), whiskers extend to include the furthest data points within 1.5× the interquartile range from box edges, and data points outside this range are represented as dots. *n* = 16 translaminar, *n* = 11 DL-restricted, *n* = 7 UL-restricted. **i**, Overview of scRNA-seq analysis in *mTmG*;*Emx1*^*cre/+*^ cortical organoids. *Emx1*^*+*^ neuronal lineage cells were extracted (black dots) and subjected to dimensionality reduction, clustering and trajectory analysis. **j**, UMAPs with cell types as in Fig. 1g and predicted trajectories using different dimensionality reduction and clustering parameters. For details see Extended Data Fig. 15. Source data

**Extended Data Fig. 12:**
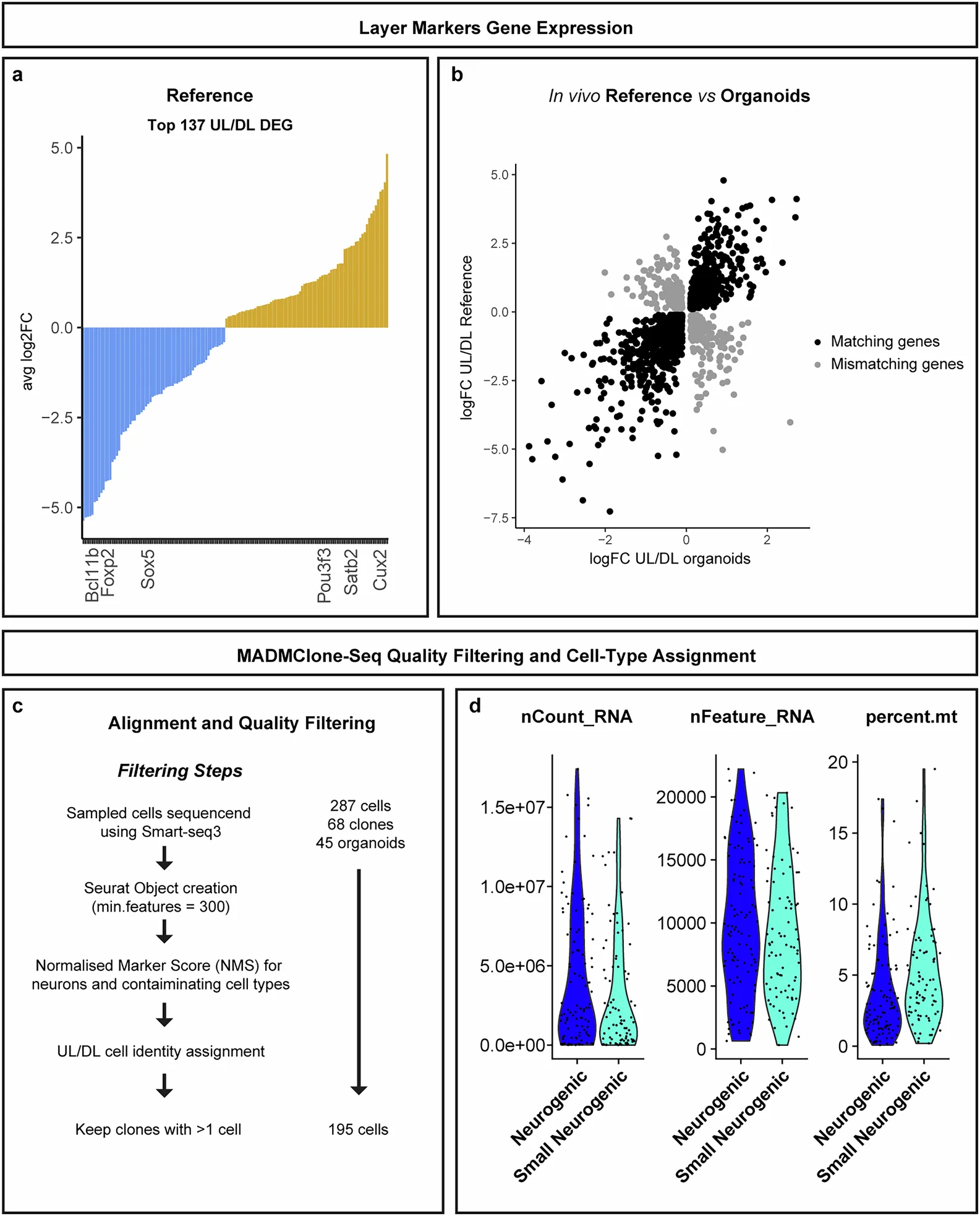
Layer marker gene expression from scRNAseq data and quality control, cell type assignment, and filtering of MADM-CloneSeq data. **a**, Average log2 fold change of top 137 differentially expressed genes from comparison of UL/DL reference cells^26^ with 6 key marker genes. **b**, Correlation analysis of average log2 fold changes of 2106 genes, significantly differentially expressed (adjusted *p*-value < 0.01) in comparison of UL/DL cells in both organoid and in vivo mouse data^26^. In total 81,5% of genes (1716/2106, *p* < 2,2 × 10^−16^ Chi-square) show matching expression in both organoid and in vivo mouse. **c**, Quality control and filtering pipeline of MADM-CloneSeq data, resulting in 195 high quality neurons. **d**, Number of transcripts (left), number of genes (middle) and % mitochondrial reads (right) detected in 195 MADM-CloneSeq cells.

**Extended Data Fig. 13:**
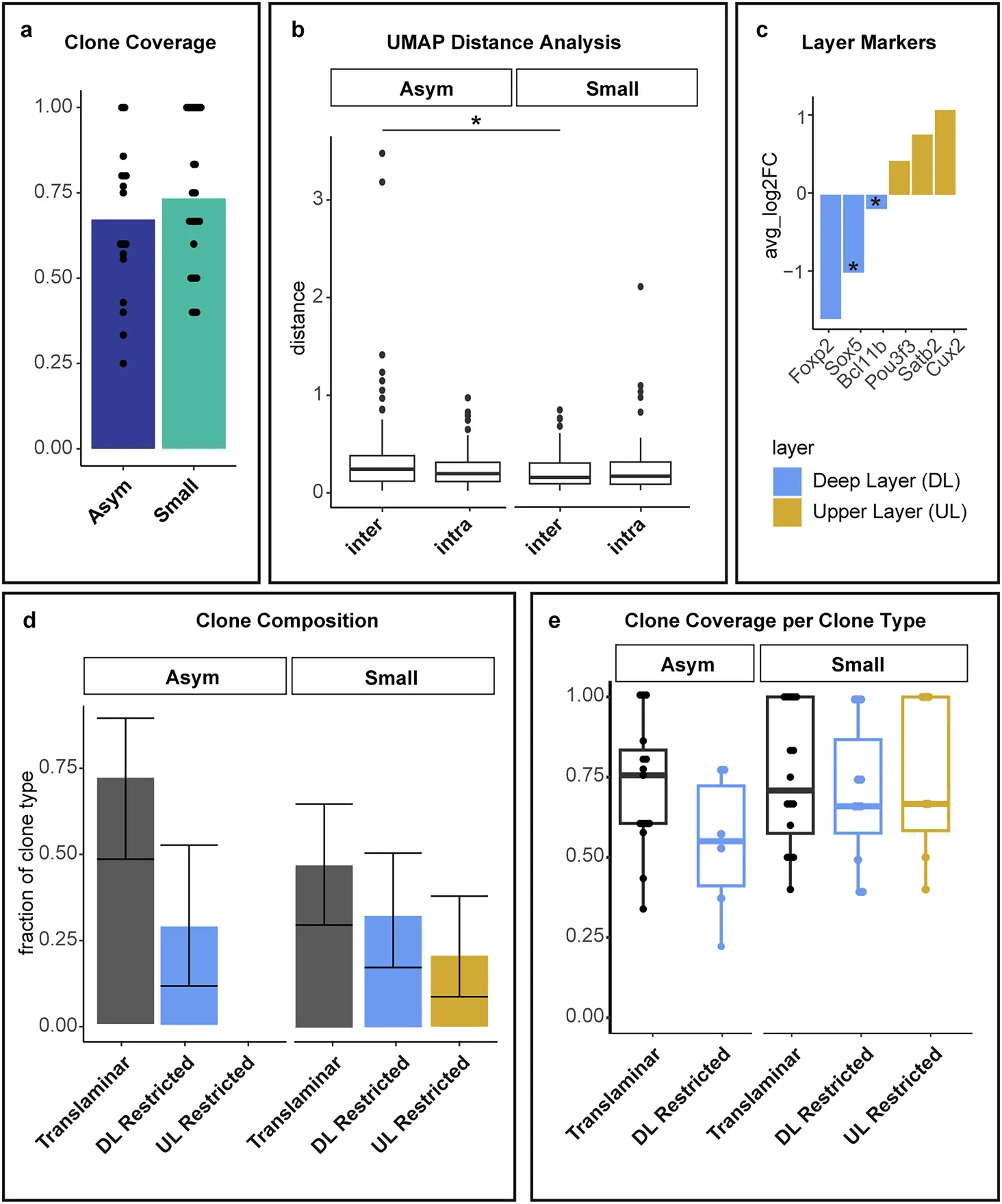
Analysis of individual cells in asymmetric and small neurogenic clones assessed by MADM-CloneSeq transcriptome data. **a**, Clone coverage defined as fraction of cells in a clone with high quality MADM-CloneSeq transcriptomes was comparable between asymmetric and small neurogenic clones. **b**, Box plots show the distribution of nearest neighbor distance (in 3D UMAP space). The nearest neighbor distance was calculated for each cell either within its clone type (small neurogenic-small neurogenic, asymmetric neurogenic-asymmetric neurogenic; intra distance) or between cells of different clone types (small neurogenic-asymmetric neurogenic, asymmetric neurogenic-small neurogenic; inter distance) in 3D UMAP space. Note: only ‘inter’ clone distances showed significant difference (adjusted *p* = 0.017, Two-way ANOVA with Tukey HSD). **c**, Average log2 fold change from differential expression analysis of MADM-CloneSeq cells assigned as upper layer (UL) / deep layer (DL). Key marker genes are listed. Asterisks indicate adjusted *p*-value < 0.1 (MAST hurdle model, two-tailed LRT: Sox5 p-value = 0.005, Bcl11b p-value = 0.043). **d**, Bar plot showing fraction of clones with deep layer (DL) or upper layer (UL) restricted clone composition (restricted); clones containing both DL and UL neurons (translaminar). Error bars represent 95% Clopper Pearson confidence interval. **e**, Clone coverage defined as fraction of cells in a clone with high quality MADM-CloneSeq transcriptomes, separated for DL and UL restricted-, and translaminar clones. (**a-e**) n = 21 asymmetric clones (109 cells), n = 34 small neurogenic clones (86 cells). (**b**, **e**) The centre line indicates the median, the box limits show the 25–75% IQR, whiskers extend to include the furthest data points within 1.5x the IQR from box edges, and data points outside this range are represented as dots. (**d-e**) n = 6 Asym DL restricted, n = 15 Asym translaminar, n = 11 SN DL restricted, n = 16 SN translaminar, n = 7 SN UL restricted (‘n’ denotes clones). Source data

To corroborate the above findings using a different methodology and to gain further insight into the transcriptomic features of lineage-restricted clones, we established MADM-CloneSeq^32^ in cortical organoids. This approach combines MADM clone generation with scRNA-seq in situ to determine the transcriptional identity of clonally related cells, offering an unprecedented correlation of lineage relationship, progenitor division mode and transcriptomic features while preserving spatial information (Fig. 4a). In total, we collected 287 cells from 68 asymmetric neurogenic and small neurogenic MADM clones induced at D13 from 45 cortical organoids and performed RNA sequencing using SMART-seq v3 technology (Fig. 4a). To assess the transcriptional identity of MADM-CloneSeq cells, we created an integrated scRNA-seq dataset, including MADM-CloneSeq cells, 64,647 cells from all *mTmG*;*Emx1-cre* organoid replicates (RGP, intermediate progenitor and neurons from D13, D20 and D25; see above), and 49,039 cells from an in vivo developmental time course of cortical development (E12–P1^26^; Methods). By using reference annotation and marker gene expression, we annotated neurons with upper layer (UL) and deep layer (DL) identity (Fig. 4b) in the reference dataset and confirmed the presence of neurons with UL or DL transcriptional identity in our organoid scRNA-seq dataset (Extended Data Fig. 12a,b). We applied a stringent filtering pipeline and retained 195 MADM-CloneSeq cells from 21 asymmetric neurogenic and 34 small neurogenic clones that were similar in quality and clone coverage (Extended Data Figs. 12c,d and  13a). Cells associated with asymmetric neurogenic and small neurogenic clones showed no major transcriptional differences, based on the analysis of projecting MADM-CloneSeq cells onto the reference UMAP and nearest neighbour analysis (Fig. 4b–d and Extended Data Fig. 13b). We assigned each MADM-CloneSeq cell to DL and UL neuronal identities to unequivocally identify lineage-restricted clones (Extended Data Figs. 12a and  13c–e). Notably, we found lineage-restricted clones among both asymmetric and small neurogenic clones with similar abundance, as reported for histological analysis (Chi-square test, *P* > 0.3) (Extended Data Fig. 13d). To test whether lineage restriction observed in organoids was associated with putative developmental delay, we performed pseudotime analysis of asymmetric and small neurogenic clones (Fig. 4e–h) and found no significant difference between lineage-restricted and non-restricted (translaminar) small neurogenic clones (Fig. 4h).

## Unitary lineage trajectory in organoids

**Extended Data Fig. 14:**
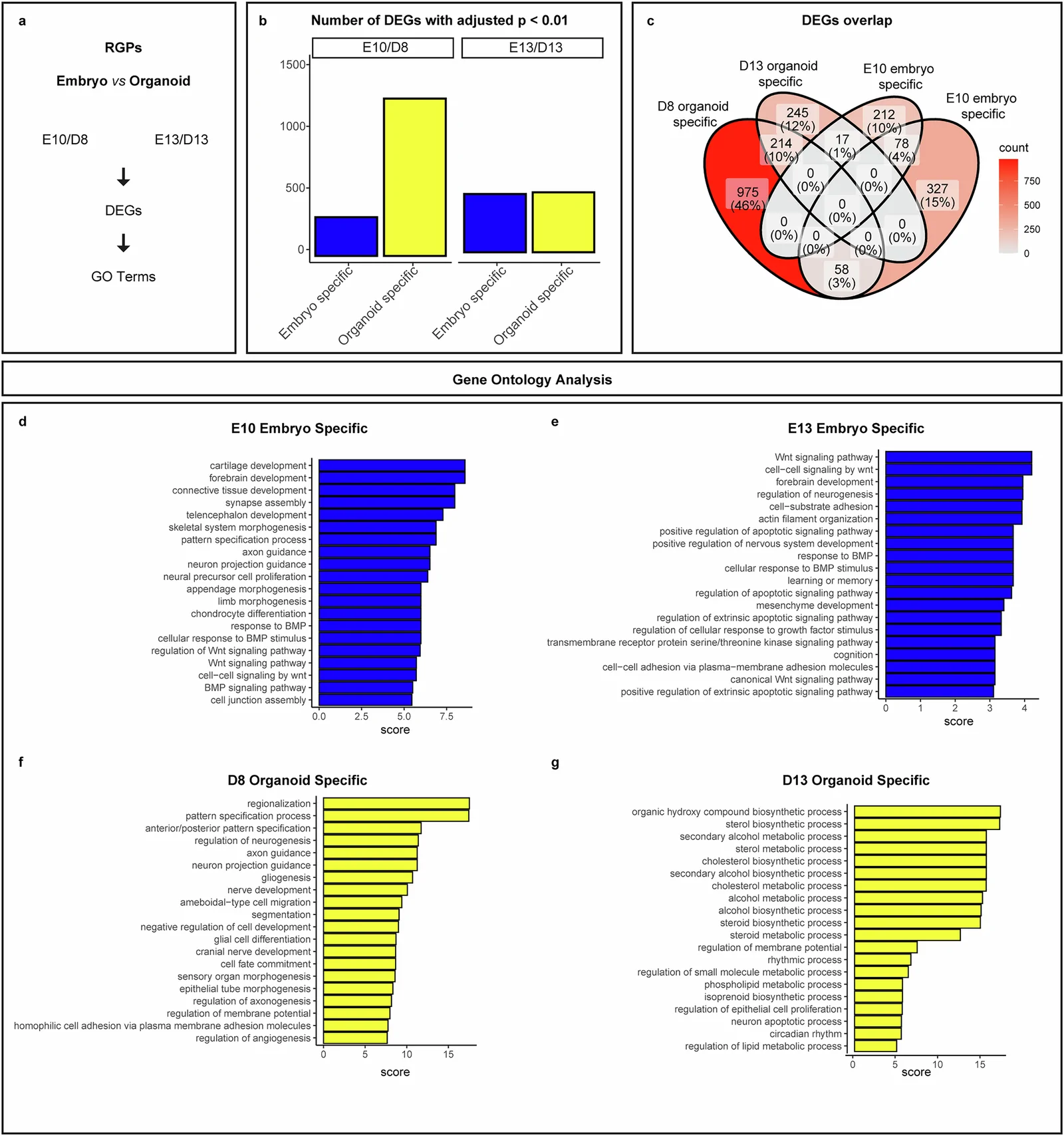
Analysis of differentially expressed genes in organoid and in vivo mouse RGPs. **a**, Overview of the RGP analysis in vivo embryo versus in vitro organoid. **b**, Number of differentially expressed genes (DEGs) in embryo (E) versus organoid (D) with adjusted *p*-value < 0.01 (Wilcoxon Rank Sum test with Bonferroni correction, two-tailed) at E8 versus D8 (left) and E13 versus D13 (right). **c**, Analysis of DEG overlap in embryo and organoid at distinct stages. **d-g**, Top 20 terms from Gene Ontology enrichment analysis for embryo specific genes from E10/D8 comparison (**d**) and E13/D13 comparison (**e**), and organoid specific genes from E10/D8 comparison (**f**) and E13/D13 comparison (**g**).

**Extended Data Fig. 15:**
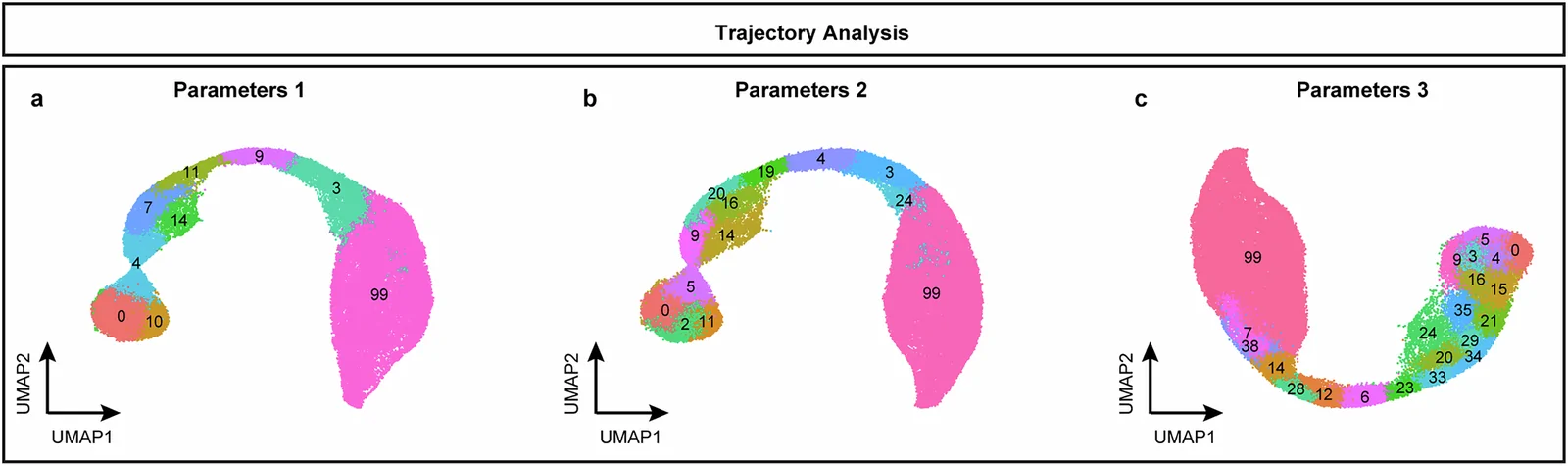
Dimensionality reduction and clustering parameter testing for trajectory analysis. **a-c**, UMAPs and unbiased Seurat clustering for cells from organoid *Emx1* neuronal lineage with different parameters (dimensionality reduction: n_neighbors, min_dist and clustering: res): res: 1, n_neighbors: 10, min_dist: 0.1 (**a**), res: 2, n_neighbors: 20, min_dist: 0.2 (**b**), res: 4, neighbors: 30, min_dist: 0.3 (**c**). Note that we specifically tested for trajectories originating from RGPs to neurons and focused the analysis by combining more mature neurons to cluster 99.

The presence of lineage-restricted and small neurogenic clones in the organoids raised the question about putative differences in RGPs and/or their lineage trajectories in vivo versus in vitro. However, scRNA-seq analysis did not show any indication of an organoid-specific RGP population, but instead indicated a homogenous pool of RGPs (Fig. 1g,j). Yet, direct analysis of transcriptional differences between organoid and embryonic RGPs revealed hundreds of differentially expressed genes (DEGs) that were largely specific for a particular developmental age (Extended Data Fig. 14a–c). Gene Ontology (GO) term analysis indicated that these DEGs were connected to biological processes such as regionalization, metabolism, development and signalling (Extended Data Fig. 14d–g). To assess whether gene expression differences in organoid RGPs might have an effect on their developmental trajectories, we focused on the integrated organoid scRNA dataset. We performed UMAP clustering and trajectory analysis using slingshot and identified a single unitary trajectory connecting RGPs via intermediate progenitors to neurons, which was consistent using several different UMAP and clustering parameters (Fig. 4j and Extended Data Fig. 15). However, we do not exclude the possible presence of minor diverging sublineages or subtle progenitor heterogeneity. On the basis of the above results, we conclude that RGPs in self-organizing cortical organoids form a transcriptionally uniform group of progenitor cells, despite showing distinct proliferation (division) patterns at any given differentiation time point, and demonstrating lineage restriction in one-third of cases in clonally related progenies.

## Discussion

**Fig. 5:**
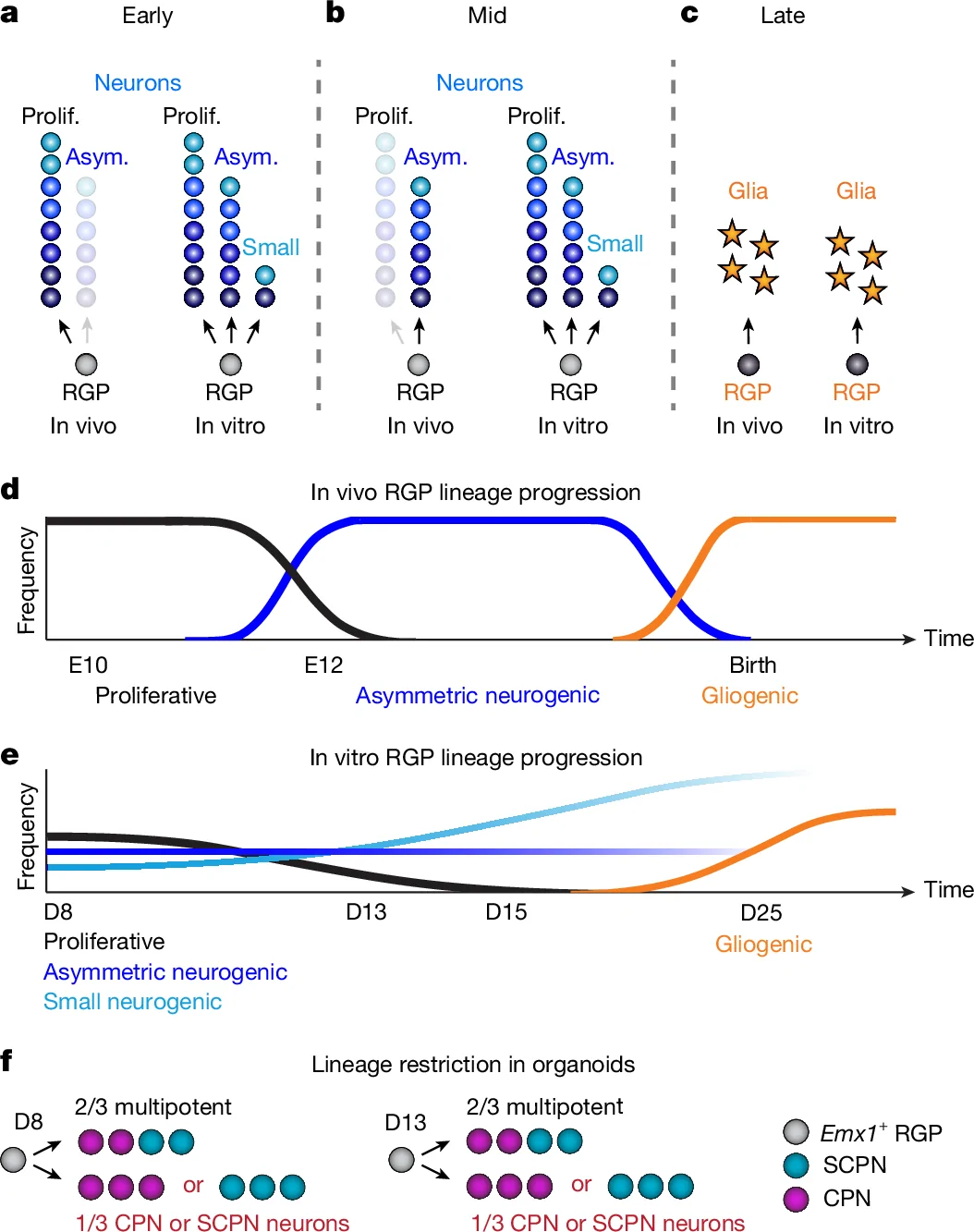
RGP cell lineage progression models in vivo and in a self-organizing cortical organoid system. **a**–**c**, Schematic illustrating cortical RGP lineage progression in vivo and in vitro during early (**a**), mid (**b**) and late (**c**) corticogenesis. **a**, In vivo, RGPs divide in a proliferative (prolif., symmetric division) manner (black) at the onset of corticogenesis, whereas in vitro we observed proliferative (black), asymmetric neurogenic (blue) and small neurogenic (light blue) RGP clone architectures concurrently, implying the respective division modes and thus no division mode bias at early stages of organoid differentiation. **b**, During mid neurogenesis, in vivo RGPs divide in an asymmetric manner (blue), producing post-mitotic neurons or intermediate progenitors with each division. RGPs in vitro continue to produce all three clone types until at least D13, after which we observe asymmetric and predominantly small neurogenic divisions. **c**, Once neurogenesis is complete, both in vivo and in vitro RGPs undergo neurogenic-to-gliogenic transition. In mice, gliogenesis begins around birth, whereas in organoids we observed the first glia at approximately D20, peaking at D25. **d**,**e**, Summary of RGP lineage progression in mouse (**d**) and the mouse cortical organoid system (**e**) over time. The black line represents proliferative divisions, the dark blue line represents asymmetric neurogenic divisions, and the light blue line represents small neurogenic divisions observed in the mouse cortical organoid system. **f**, Summary of RGP lineage restriction in the cortical organoid system over time. At both D8 and D13, around one-third of RGPs generate exclusively SCPNs or CPNs. Compared with estimated lineage restriction in vivo, the observed rates in the organoid system were about an order of magnitude higher.

Self-organization constitutes a major factor that regulates a range of developmental processes without significant control from external agents^33^. Recent work has demonstrated that self-organization instructs the faithful formation of all germ layers and major tissues in embryos from pluripotent embryonic stem cells in isolated culture systems^34–37^. Here, to test the rigidity of self-organization in driving stem cell lineage progression, we probed lineage progression of RGPs (representing committed multipotent NSCs) in a cortical organoid culture system derived from mESCs. By using MADM-based clonal analysis, we determined the potential, proliferation behaviour and lineage progression of RGPs across differentiation at single-cell level. As a blueprint for comparison to the in vivo context, we leveraged our previously established framework that emerged from lineage tracing experiments that used identical genetic constituents for marking RGPs and their clonal progeny^1^. Whereas RGP lineage progression follows a strict linear and temporally stereotyped proliferation pattern and output potential in vivo, RGPs at any differentiation time point in organoids showed no bias in cell division capacity (Fig. 5) and more variable neuron output potential. At the qualitative level, RGP lineages in cortical organoids showed a high level of lineage restriction (that is, strong bias towards reduced neuron diversity within units of clonally related cells) independent of the differentiation time point. Thus, the integrity of developmental and/or temporal progression was lost or severely altered, possibly resulting in more flexible and/or permissive competence windows. Of note, the generation of glia occurred strictly after neurogenesis, similar to a number of stem cell niches in vivo^1,32,38,39^.

RGPs in vivo have been shown to exhibit a certain level of plasticity in response to a changing environment^40^. Thus, the absence of an endogenous stem cell niche and therefore various altered non-cell-autonomous cues in cortical organoids could result in altered temporal plasticity and/or sensitivity to the in vitro tissue environment. Although the precise nature of the non-cell-autonomous, niche-derived cues remains unclear, a variety of factors have been implicated, also considering radial glial development in humans^8,41^. Extracellular matrix^42^, extracellular vesicles^43,44^, mechanical support cells such as the radial glial fibre grid^45,46^, microglia^47^, immature nascent projection neurons, the absence of interneurons, blood vessels, choroid plexus and cerebrospinal fluid, missing axon targets (ref. ^48^ and references therein) and certain metabolic microenvironments^28^ could have effects on RGP lineage progression. Of note, our comparison of RGPs from distinct in vivo and in vitro niche environments indicates that predictions of biological function of DEGs using GO suggest metabolic changes, altered cell–cell interactions (adhesion) and responses to external signalling cues. Thus, our gene expression data may provide some basis for future hypothesis generation and directions.

Our clonal dataset could imply that the entire population of RGPs in cortical organoids are more plastic and responding increasingly or only to stochastic means. Alternatively, they may have differentiated into distinct RGP types and/or RGP cell states (owing to the altered cellular environment) that would directly or indirectly result in the observed diversity of lineages, including the prominent small neurogenic clones and increased lineage-restricted clones. Prior studies have probed RGP cell and lineage diversity in vivo by using fate mapping^49,50^ and/or transcriptome assessment at the single-cell level^26,51^. At the population level and depending on the phenotype manifestation, RGP lineages may be grouped into distinct classes^52^. Yet, scRNA-seq data do not show significant clustered gene expression that would segregate and/or correlate with distinct RGP types at the transcriptional level in vivo. Our data show that RGPs in cortical organoids also form a uniform group of progenitors with a unitary lineage trajectory, similar to in vivo data^26,53^, despite altered proliferation behaviour and output. Prospective probing of RGP lineage progression rules in isolated systems across species, including human in normal and pathogenic conditions^54,55^, will help us to decipher the principles that drive NSC evolution and the aetiology of neurodevelopmental diseases.

## Methods

### Maintenance of mouse lines

All animal procedures were approved by the Austrian Federal Ministry of Women, Science and Research in accordance with the Austrian and European Union animal law (license number: BMWF-66.018/0007-II/3b/2012; BMWFW-66.018/0006-WF/V/3b/2017; GZ: 2020-0.579.989 and GZ: 2025-0.597.515). Experimental mice were bred and maintained according to regulations approved by the institutional animal care and use committee and institutional ethics committee and the guidelines of the preclinical facility (PCF) at ISTA. Mice with specific pathogen-free status according to FELASA recommendation^57^ were bred and maintained in experimental rodent facilities (room temperature 21 ± 1 °C (mean ± s.e.m.); relative humidity 40–55%; photoperiod 12 h light:12 h dark). Food (V1126, Ssniff Spezialitaten) and tap water were available ad libitum. Mouse lines with MADM cassettes inserted on chromosome 11^58^, *Emx1-cre*^59^, *Emx1-creER*^60^ and *mTmG* reporter^61^ have been reported previously and were used to generate experimental mice. All mouse lines were kept in a mixed C57/Bl6 and CD1 genetic background. Mice were used at an age range of 2–8 months for general breeding, 2–4 months (females) for collecting blastocysts, and at P21 for clonal analysis experiments in vivo. All efforts were made to minimize the number of animals by following the 3R principles.

### Timed breeding and superovulation for the generation of genetically defined blastocysts

*MADM-11*^*TG/TG*^ and *MADM-11*^*GT/GT*^;*Emx1*^*cre/+*^ or *MADM-11*^*GT/GT*^;*Emx1-creER*^*+/−*^ stock mice were crossed to generate *MADM-11*^*GT/TG*^;*Emx1*^*cre/+*^ or *MADM-11*^*GT/TG*^;*Emx1-creER*^*+/−*^ blastocysts, respectively. For scRNA-seq experiments (see ‘scRNA-seq’), *mTmG* reporter mice were crossed to *Emx1*^*cre/+*^ mice to generate *mTmG*;*Emx1*^*cre/+*^ blastocysts. Superovulation was performed according to the ISTA Preclinical Facility protocol. In brief, to synchronize the oestrous cycle and induce superovulation, 0.1 ml (5 IU) pregnant mare serum gonadotropin (PMSG; Sigma) was administered by intraperitoneal injection during the afternoon (between 16:00 and 18:00) of day −3 before ovulation. On day −1 (46–48 h after PMSG), 0.1 ml (5 IU) human chorionic gonadotropin (Sigma) was administered by intraperitoneal injection and the female immediately added to the male cage. Hormones in lyophilized powder form were resuspended in Dulbecco’s PBS (Sigma) and stored in aliquots at −20 °C until use.

### Derivation and culture of mESCs

Blastocyst collection at E3.5 and derivation of mESCs from individually cultured blastocysts were performed as described previously^62^. Blastocysts were flushed from the uterine horn with M2 medium (Sigma) using a syringe, collected with a micropipette and washed with 1 ml of M2 medium. Blastocysts were cultured for up to 24 h in KSOM medium (Sigma) until expansion of the blastocoel and/or hatching was observed. Each blastocyst was transferred to a single well in a 96-well plate that was prepared with mouse embryonic fibroblasts (MEFs; Thermo Fisher Scientific) the day before at a density of 1.5 × 10^4^ cells per well. Blastocysts were cultured in KO-DMEM (Thermo Fisher Scientific) medium containing 15% knockout serum replacement (Thermo Fisher Scientific), 1 mM sodium pyruvate (Thermo Fisher Scientific), 0.1 mM non-essential animo acids (Thermo Fisher Scientific), 0.1 mM 2-mercaptoethanol (Sigma), 2 mM GlutaMAX (Thermo Fisher Scientific), 50 U ml^−1^ penicillin/streptomycin (Thermo Fisher Scientific), 2i (1 µM PD0325901 and 3 µM CHIR99021, Sigma) and LIF (1 2 ng ml^−1^, batch tested, Thermo Fisher Scientific). Once large outgrowth was observed (~7 days) the cells were passaged for the first time. mESCs were maintained on MEFs in medium containing ES-qualified FBS (Thermo Fisher Scientific)/LIF, with passaging every 3 days on average. mESCs were moved off MEFs 2 passages before generating organoids, and were plated on EmbryoMax 0.01% gelatin (Sigma) coated wells in medium containing FBS/LIF/2i^63^ for a maximum of 10 passages. mESCs were routinely tested for mycoplasma using the LookOut Mycoplasma PCR Detection Kit (Sigma). Standard cell culture conditions (37 °C with 5% CO_2_) were used throughout all procedures. Early passage (P3–P8) cells were frozen in liquid nitrogen cryovials in large stocks, using ES-qualified FBS with 20% DMSO as the freezing medium. Early passage stocks were used for all experiments (final passage of cells used to make organoids between P8 and P15).

### Methanol fixation for karyotyping of mESC lines

Methanol fixation was performed according to instructions provided by Cell Guidance Systems’ karyotyping service. In short, cell cultures in 6-well plates were incubated with medium supplemented with 10 µg µl^−1^ KaryoMAX colcemid solution (Thermo Fisher Scientific) for 30 min at 37 °C. Medium was removed and colonies were dissociated with 400 µl of pre-warmed 0.05% Trypsin EDTA (Thermo Fisher Scientific) for 5 min at 37 °C. Trypsin was deactivated with 800 µl of warm medium and cells were transferred to a 15 ml conical tube and centrifuged for 5 min at 200*g*. The supernatant was discarded and the cell pellet was broken by flicking the tube 20 times. Cells were treated with 2 ml of warm 0.075 M KCl drop by drop, followed by a further 2 ml dispensed slowly down the wall of the tube. The suspension was mixed by inversion and incubated for 15 min at 37 °C. Next, 10 drops of cold freshly prepared fixative (3 parts methanol (VWR) to 1 part acetic acid (VWR) by volume) were added, using a 1 ml Pasteur pipette. The suspension was mixed by gentle inversion. Samples were centrifuged for 5 min at 150*g*, the supernatant was discarded and the pellet was broken by flicking the tube 20 times. Cells were washed with 4 ml of cold fixative, added very slowly, and centrifuged for 5 min at 150*g*. The supernatant was discarded, pellet broken, and cells were finally resuspended in 1.5 ml fixative. Samples were shipped off to Cell Guidance Systems’ karyotyping service. Chromosome 8 and 11 abnormalities have been previously reported in mESC lines^64,65^.

### Metaphase spread for chromosome counts

Cells for metaphase chromosome spreads were fixed as described above. In preparation, Superfrost glass slides (Thermo Fisher Scientific) were cooled at −20 °C for 5 min. 100 µl of the fixed cell suspension was dropped, as a single drop, from 10–15 cm above the slide. The slide was air dried, and a coverslip was placed with Mowiol 4-88 (Carl Roth) and 1,4-diazabicyclooctane (Carl Roth) with DAPI (4′,6-diamidino-2-phenylindole, Thermo Fisher Scientific, 1:5,000 dilution) to stain DNA. Slides were imaged with a Plan-Apochromat 40×/1.2 water immersion objective using an inverted LSM 800 series confocal microscope (Zeiss), and images were processed using Zeiss ZEN Blue 2.3 and 2.6 software (Zeiss). Chromosomes were counted manually in the Zeiss ZEN Blue 2.6 software (Zeiss) and plotted using Graphpad Prism 10.2.2 (Dotmatics) software.

### Generation of cortical organoids

mESCs were plated at high density so that colonies covered approximately half of the well area after 2 days of incubation. Medium was changed 1–2 h prior to dissociation with StemPro Accutase (Thermo Fisher Scientific) or CTS TrypLE (Thermo Fisher Scientific) for 5 min at 37 °C. Cells were counted with an automated cell counter, and were used only if >90% of cells were alive based on Trypan blue staining (Thermo Fisher Scientific). mESCs were centrifuged for 5 min at 200*g*, resuspended in Solution 1, and plated at a density of 3,500 cells per microwell in a 24w-Aggrewell plate (Stem Cell Technologies). Solution 1 contained G-MEM (Thermo Fisher Scientific), 10% knockout serum replacement (Thermo Fisher Scientific), 1 mM sodium pyruvate, 0.1 mM non-essential amino acids, 0.1 mM 2-mercaptoethanol, 2 mM GlutaMAX, 50 U ml^−1^ penicillin/streptomycin and Wnt inhibitor (3 µM, IWR1, Sigma). No SMAD inhibitors were used as they were not required to direct cortical differentiation in mouse^66^. Embryoid bodies were generated by D1, which were then transferred to 10 cm dishes with fresh Solution 1 and 2% growth factor-reduced Matrigel (Corning). On D5, organoids were gently pipetted to remove Matrigel and Solution 2 was added, containing DMEM/F12 (Thermo Fisher Scientific), 1% N2 supplement (Thermo Fisher Scientific), 0.1 mM non-essential amino acids, 2 mM GlutaMAX, 50 U ml^−1^ penicillin/streptomycin, 1% chemically defined lipid concentrate (Thermo Fisher Scientific), and heparin (1 μg ml^−1^; Sigma). On D7, organoids were transferred to 6-well plates and placed on an orbital shaker at low speed (50 rpm) to prevent organoid fusion. From D9, organoids were grown in medium containing a 50:50 mix of DMEM/F12 and Neurobasal (Thermo Fisher Scientific), 0.5% N2, 1% B27 (Thermo Fisher Scientific), 0.5 mM non-essential amino acids, 2 mM GlutaMAX, 50 U ml^−1^ penicilin/streptomycin, 1% chemically defined lipid concentrate, and 50 μM 2-mercaptoethanol. Throughout the protocol medium was replaced as necessary every 2–3 days, and orbital shaker speed was increased after D9 (80 rpm). Fused organoids and/or organoids that failed to grow were discarded throughout the procedure. Standard cell culture conditions (37 °C with 5% CO_2_) were applied throughout all procedures.

### Induction of MADM clones in cortical organoids

Stock solutions of 4-hydroxytamoxifen (4-OHT, Sigma) were generated by dilution in 100% ethanol to a concentration 1,000-fold greater (20–80 µM) than the working solution. 4-OHT was titrated in order to generate ~1 clone per organoid on average, which varied per time point: a maximum of 80 nM for D8, 40 nM for D9–D12, 60 nM for D13, and 80 nM for D15. If 4-OHT treatment occurred on a day without medium change, medium was removed from wells containing organoids to be treated and placed in a tube, 4-OHT was added, and medium was added back to wells. After 24 h, medium was completely replaced and organoids were washed once with PBS. Organoids were collected at D20 for MADM clonal analysis, except for astrocyte analysis, which included collection at D25. For MADM clone analysis, each clone initiation time point included clones from 3 cell lines and 3 differentiations, except for D9, D11 and D12, which included clones from 2 cell lines and 4 differentiations. The numbers of clones per organoid per clone initiation time point were as follows: D8–D20 (109 clones/100 organoids = 1.09), D9–D20 (46 clones/49 organoids = 0.94), D10–D20 (93 clones/165 organoids = 0.56), D11–D20 (50 clones/67 organoids = 0.75), D12–D20 (61 clones/50 organoids = 1.22), D13–D20 (68 clones/133 organoids = 0.51), D15–D20 (40 clones/63 organoids = 0.63) and D8–D25 (39 clones/92 organoids = 0.42).

### Induction of MADM clones in vivo

MADM clone induction in vivo was performed as described^56,58^. In brief, *MADM-11*^*GT/TG*^;*Emx1-creER*^*+/−*^ experimental mice were generated by crossing *MADM-11*^*GT/GT*^;*Emx1-creER*^*+/−*^ with *MADM-11*^*TG/TG*^ mice. Timed pregnant females were administered 2 mg tamoxifen (Sigma) dissolved in corn oil (Sigma) by intraperitoneal injection at E10 to induce MADM clones. Live embryos were recovered via caesarean section at E18–E19 and fostered until P21. Both male and female specimens were used. In total, 30 MADM clones were obtained from 37 brains, averaging 0.81 clones per brain.

### Tissue collection and cryosectioning

Organoids were fixed for 4 h or overnight in 4% paraformaldehyde (Sigma). Tissue collection from mice was performed according to previously described protocols^56^. Mice were deeply anaesthetized with an intraperitoneal injection of ketamine/xylazine (65 mg kg^−1^ and 13 mg kg^−1^ body weight, respectively), and were confirmed to be unresponsive through pinching the paw. Perfusion was performed with ice-cold PBS followed by ice-cold 4% paraformaldehyde prepared in PBS using a peristatic pump (Carl Roth, 4–6 ml min^−1^). Mouse brains were further fixed overnight in 4% paraformaldehyde, then washed with PBS. Organoids and mouse brains were cryopreserved with 30% sucrose in PBS for 1 and 2–3 days, respectively. Tissues were embedded in Tissue-Tek O.C.T. (Sakura) and stored at −20 °C or −80 °C until further use. All samples were cryosectioned using a CryoStar NX70 cryostat (Thermo Fisher Scientific). Brains were sectioned coronally (45 µm thickness) and were collected in PBS, and mounted onto glass slides in sequential order. Organoids were sectioned (40 µm thickness) and directly mounted onto Superfrost glass slides (Thermo Fisher Scientific). Mounted sections were air dried while protected from light and processed immediately for analysis (mouse tissue) or were stored at −20 ^o^C until use (organoid).

### Immunostaining

Cryosections mounted on glass slides were first rehydrated with PBS for 15 min at room temperature. For antibodies requiring antigen retrieval (PAX6, TBR2, CTIP2, SATB2, OCT3/4, NANOG and GFAP), samples were incubated in citrate buffer (10 mM citric acid, 0.05% Tween-20; pH 6.0) for 25 min at 85 °C. After cooling to room temperature and washing 3× with PBS, samples were incubated for 2 h at room temperature in blocking solution (0.5% Triton X-100 (Thermo Fisher Scientific) with 10% Donkey Serum (Thermo Fisher Scientific) in PBS. Primary antibodies were diluted in blocking solution, and samples were incubated for 16–72 h at 4 °C. After washing (3× 15 min) with PBS, secondary antibodies were diluted in blocking solution, and samples were incubated for 2–24 h at room temperature. After washing (3× 15 min) with PBS, cell nuclei were stained with DAPI (Thermo Fisher Scientific, 1:5,000 dilution) for 15 min. All sections were mounted using Mowiol 4-88 (Carl Roth) and 1,4-diazabicyclooctane (Carl Roth) and stored at 4 °C until image acquisition.

### Antibodies

Primary antibodies and dilution factors included chicken anti-GFP (Aves, GFP1020, 1:1,000), goat anti-tdTomato (SICgen, ab8181-200, 1:1,000), rabbit anti-PAX6 (Cell Signaling, 60433S, 1:500), rat anti-TBR2 (Thermo Fisher Scientific, 14-4875-82, 1:400), rat anti-CTIP2 (ab18465, 1:400), mouse anti-SATB2 (Abcam, ab51502, 1:200), rabbit anti-NANOG (Abcam, ab80892, 1:500), mouse anti-OCT3/4 (Santa Cruz, sc5279, 1:200), rabbit anti-GFAP (DAKO, Z0334 1:1,000), rabbit anti-NEUROD2 (ab109406, 1:200) and rabbit anti-CASPASE3 (Cell Signaling, 9661S, 1:400). Secondary antibodies and dilution factors included donkey anti-chicken-Alexa488 (Jackson Immuno, 703-545-155, 1:1,000), donkey anti-goat-Alexa568 (Invitrogen, A11057, 1:1,000), donkey anti-goat-CY3 (Jackson Immuno, 705-165-147, 1:1,000), donkey anti-rat-Alexa594 (Life Technologies, A21205, 1:1,000), donkey anti-mouse-Alexa647 (Life Technologies, A32787, 1:1,000) and donkey anti-rabbit-Alexa647 (Life Technologies, A31573, 1:1,000). For MADM clones that were immunostained with GFAP (using donkey anti-rabbit-Alexa647), we used donkey anti-chicken-Alexa488 and donkey anti-goat-Alexa568 for immunostaining of MADM-labelled cells. For MADM clones that were immunostained with SATB2 (donkey anti-mouse-Alexa647) and CTIP2 (donkey anti-rat-Alexa594), we used donkey anti-chicken-Alexa488 and donkey anti-goat-CY3 for immunostaining of MADM-labelled cells.

### Image acquisition and analysis of MADM clones

Before image acquisition, immunostained samples were first screened for MADM clones using an axioscope (Zeiss Axio Imager, Zeiss) coupled to a CoolLED p300 SB light source (CoolLED) and equipped with Plan-Apochromat 10×/0.45 and 20×/0.8 objectives (Zeiss). Green and red fluorescence were observed using an HC-dualband GFP/DsRed filter (F56-420, AHF). The presence of MADM-labelled cells was documented for subsequent confocal image acquisition. Organoid and mouse brain MADM clones were imaged with a Plan-Apochromat 20×/0.8 objective using an inverted LSM 800 series confocal microscope (Zeiss), and images were processed using Zeiss ZEN Blue 2.3 and 2.6 software (Zeiss). Confocal images were acquired in *z*-stacks and tiles with excitation lasers 405, 488, 561, and 640 nm. Five-channel imaging (DAPI, GFP, tdT, CTIP2 and SATB2) was specifically performed with a Leica Stellaris 5 series confocal microscope (Leica) and an HC PL APO 20×/0.75 CS objective, using a white light laser optimized by the software for all fluorophores (A488, CY3, A594, A647) except DAPI (excitation laser 405). Images were processed using LAS X 2.5.7.23225 (Leica) software. Images were imported into ImageJ (Fiji) where MADM-labelled cells were manually counted based on marker expression. MADM clone architecture was reconstructed by using previously published protocols^29^. In short, proliferative clones are clones in which subclone sizes are ≥4 for both (red and green) subclones. Asymmetric neurogenic clones have one subclone with ≥4 cells and one subclone with <4 cells. Small neurogenic clones are those in which subclone sizes are ≤3 cells for both subclones.

#### Laminar positioning in MADM clones

Figure 3l, in vivo data. Data are based on laminar position as determined by DAPI staining (2 out of 263 MADM clones induced at E10–E12^1^ and 9/106 MADM clones induced at E12)^9^, and laminar-specific immunostaining (0 out of 17 MADM clones at E10–E12)^1^.

### Organoid clearing, imaging and analysis

#### Delipidation

Organoids were fixed as described above; one modification to note was that the sucrose dehydration step was skipped. After fixation, organoids were embedded in 1.5% agarose, to provide structural support during the clearing process. The organoid clearing protocol was adapted from previously published tissue clearing methods^67–69^. In brief, embedded organoids were first washed in a 50% solution of 1:1 CUBIC-L (10% w/v *N*-butyldiethanolamine, 10% w/v Triton X-100 in dH_2_O):CUBIC-R1a (5% w/v *N*,*N*,*N*′,*N*′-tetrakis, 10% w/v urea, 10% w/v Triton X-100 1:200 5 M NaCl in dH_2_O) in ddH_2_O for up to 16 h at 37 °C, 300 rpm. Next, organoids were incubated in 100% 1:1 CUBIC-L:CUBIC-R1a twice for 2 h each time, at 37 °C, 300 rpm. Finally, organoids were washed with PBS twice for 2 h at 37 °C, 300 rpm, and once overnight at 37 °C, 300 rpm.

#### Immunolabelling of cleared organoids

Cleared organoids were incubated with chicken anti-GFP (Aves, GFP1020, 1:500) and goat anti-tdTomato (SICgen, ab8181-200, 1:500) in 0.2% PBST (note the higher antibody concentration for cleared organoids) overnight at 37 °C, 300 rpm. The primary incubation solution was removed and organoids were washed 3× for 1 h with PBS at 37 °C, 300 rpm. Next, organoids were incubated in donkey anti-chicken-Alexa488 (Jackson Immuno, 703-545-155, 1:1,000) and donkey anti-goat-Alexa568 (Invitrogen, A11057, 1:1,000) in 0.2% PBST overnight (note the higher antibody concentration for cleared organoids) at 37 °C, 300 rpm. The secondary incubation solution was removed and organoids were washed twice for 1 h with PBS at 37 °C, 300 rpm.

#### Refractive index matching

Once washed, organoids underwent a refractive index-matching step. Organoids were transferred through gradually increasing concentrations of CUBIC-R + (N) (30% nicotinamide and 45% antipyrine in dH_2_O): 1 h steps of 25%, 50%, 75% and 100% CUBIC-R + (N) in dH_2_O. After the last step, the 100% CUBIC-R + (N) solution was replaced and organoids were stored overnight before imaging.

#### Imaging and analysis of cleared organoids

Whole cleared organoids embedded in agarose were imaged with an Andor Dragonfly 505 spinning disk system using a 20× Lambda/NA 0.75/WD 1.00 mm objective and 488 and 561 nm excitation lasers. During imaging, organoids were immersed in either fresh CUBIC-R + (N) or mineral oil with a refractive index of 1.52. Nikon ND2 files were converted and stitched using IMARIS File Converter and IMARIS Stitcher, respectively.

#### 3D Image analysis

Once files were converted and stitched, they were loaded into IMARIS v9.9.1. The surfaces of the organoid were reconstructed using the surface tool. Once reconstructed, IMARIS automatically calculated both sphericity and volume.

### scRNA-seq

#### Generation of *mTmG*;*Emx1*^*cre/+*^ organoids for scRNA-seq

mESC lines with *mTmG*;*Emx1*^*cre/+*^ genotype and corresponding cortical organoids were generated as described above. Two mESC lines (A and B) were used to generate two batches each (batch 1 and 2, made from consecutive passages of the same cell lines) in order to generate four replicates per time point (A1, A2, B1 and B2). Large batches were generated so that organoids could be collected at all four time points from the same batch, for each of the four batches.

#### Generation of *mTmG*;*Emx1*^*cre/+*^ embryos for scRNA-seq

Cortex samples were prepared using previously described protocols^70^. In brief, embryos were collected from timed-pregnant females sacrificed by cervical dislocation whereas pups were isolated from their mothers. All embryos and pups were euthanized by decapitation. Either whole heads or extracted brains were kept in ice-cold HBSS solution. Under a dissection microscope, the cortex was carefully dissected from the brain and placed in fresh ice-cold HBSS solution. The cortex samples (or telencephalic vesicles at E10.5) were isolated free of meninges, medial and ventral structures. Isolated tissues were then pooled and processed.

#### Generation and fixation of single-cell suspension

To collect 10^6^ to 2 × 10^6^ fixed cells per replicate, appropriate numbers of organoids or embryos were pooled for dissociation per replicate. Embryos: A total of 40, 30, 10 and 4 brains were pooled from 8, 4, 2 and 2 litters for E10, E13, E16 and P1 time points, respectively. Organoids: D8 organoids generated from 4–5 Aggrewells, ~800 organoids; D13: ~300 organoids; D20: 80 organoids; D25: 60 organoids). Organoids or embryos were dissociated following a previously published protocol with some modifications^71^. Organoids or embryos were first incubated in Earle’s balanced salt solution (EBSS, Thermo Fischer Scientific) containing Papain (Worthington) and DNaseI (Worthington) for a total of 20 (D8, D13) or 25 min at 37 °C with gentle shaking at 150 rpm. Organoids or embryos were gently pipetted 3× with a P1000 tip. After adding Ovomucoid I-Albumin (Worthington), tissue suspension was briefly dissociated mechanically with a pipette. After centrifugation at 1,000 rpm for 10 min at room temperature, the cell pellet was re-suspended and further mechanically dissociated until a homogeneous cell suspension was obtained (up to 20× with a P1000 tip). The cell suspension was passed through a 70-µm filter, and centrifuged again at 1,500 rpm for 10 min at room temperature. Samples were next processed to remove debris prior to fixation. Cell pellets were re-suspended and mixed with cold Debris Removal Solution (Miltenyi Biotec). The mixture was carefully overlaid with cold PBS before centrifugation at 3,000*g* for 10 min at 4 °C. The top two layers containing PBS and debris were then aspirated and removed. The remaining cleaned cell suspension was collected by a last centrifugation step at 1,000*g* for 10 min at 4 °C. Following manual cell counting with Trypan blue, 10^6^ to 2 × 10^6^ cells were collected and pelleted. Cells were then fixed with the kit and protocol for Chromium Fixed RNA Profiling (10X Genomics, protocol CG000478, RevD). Pellets were re-suspended in a solution containing 4% formaldehyde and 1× Fix and Perm Buffer, and were stored at 4 °C for 22 h. Cells were then pelleted and re-suspended in a solution containing 1× quenching buffer. Cells were counted again, followed by addition of Enhancer solution and glycerol, after which samples were stored at −80 °C until processed.

### Sample collection for MADM-CloneSeq in organoids

Organoids with MADM clones induced at D13 were collected at D20 for MADM-CloneSeq. The sample collection steps for MADM-CloneSeq were adapted from a previously published protocol^72^. Organoids were kept in cell culture membrane (Millicell) in oxygenated (95% O_2_, 5% CO_2_) artificial cerebrospinal fluid (ACSF) containing (in mM): 118 NaCl, 2.5 KCl, 1.25 NaH_2_PO_4_, 1.5 MgSO_4_, 1 CaCl_2_, 10 glucose, 3 myo-inositol, 30 sucrose, 30 NaHCO_3_ prepared with ultra-distilled water; pH 7.4, at 35 °C, until sample collection.

All work surfaces and equipment were cleaned using RNase away solution (Thermo Fisher Scientific) prior to experiments. Single organoids were transferred to a LNscope 240XY miscroscope (Luigs & Neumann) and superfused with ACSF at a rate of 0.5 ml min^−1^ at room temperature. Individual clones were visualized under a 20× objective and with ImageJ software. To observe red or green fluorescence, light was emitted from a LED light source (CoolLED). Glass pipettes (B150-86-10, Sutter Instrument) previously autoclaved, were pulled using a P1000 pipette puller (Sutter Instrument) to generate pipettes with an opening of around 3–5 μm. Before use, each pipette was filled with 3.5 μl of pipette solution, which consisted of RNase inhibitor (Takara, 1.95%) in RNase-free PBS (Invitrogen) filtered through a 0.22-μm filter. Red or green fluorescent neurons were approached with the pipette under positive pressure to avoid tissue contamination of the pipette tip. When contact with the cell membrane was made, tight seal formation was achieved under infrared differential interference contrast (IR/DIC) visualization. The complete aspiration of the cell body was immediately performed by applying gentle and steady suction while monitoring under fluorescence, which typically takes 5–10 s. The negative pressure was removed immediately once the target cell was collected and the pipette was carefully withdrawn from the acute slice to avoid contamination. The content of each pipette was directly transferred into individual PCR tubes and kept at −80 °C until cDNA library preparation.

### Preparation of cDNA libraries and sequencing

10X Genomics: experiments were performed by the Next Generation Sequencing Facility at Vienna BioCenter Core Facilities (VBCF-NGS), member of the Vienna BioCenter (VBC), Austria. Single-cell libraries were prepared based on the 10X Genomics Flex workflow according to manufacturer’s instructions (CG000527). In order to detect GFP and tdTom mRNA expression, custom probes were designed according to 10X Genomics Technical Note CG000621 and ordered from Integrated DNA Technologies (IDT) as 4 different pools, one for each FLEX barcode sequence (BC001–BC004, custom probe sequence are provided in Supplementary Table 1). Custom probes were added to the mouse probes (PN-100496, 10X Genomics) following the manufacturer’s instructions (CG000621). MADM-CloneSeq: cDNA libraries for individual MADM-CloneSeq cells were were prepared using Smart-seq3 protocol^73^ in 3 batches (96-well plates). Sequencing was performed at VBCF-NGS on NovaSeqX platform (Illumina).

### Bioinformatic analysis

#### Initial analysis 10X Flex organoid data

All bioinformatic analysis was performed using a pipeline developed in-house^74^. Fastq files were processed using refdata-cellranger-mm10-3.0.0 and cellranger v8.0.0 with GFP and tdTom sequences added to Chromium_Mouse_Transcriptome_Probe_Set_v1.0.1_mm10-2020-A.csv. Filtered feature matrix files (in h5 format) were used for downstream analyses in R v4.3.2 using the Seurat v5.0.1 package^75^. Initial filtering for high quality cells: nFeature_RNA > 1000 & nFeature_RNA < 8000 & nCount_RNA < 40000 & percent.mt <5. We determined cell clusters using FindNeighbors (dims = 1:25) and FindClusters(resolution = 0.3). Significance of differential expression of GFP was used to determine GFP expressing cell clusters. Stressed cells were determined using granular functional filtering (Gruffi)^76^. In brief, Gruffi uses gene sets from Gene Ontology (GO) pathways to calculate GO scores and identifies stressed cells by integrating multiple scores. We obtained relevant gene sets from org.Mm.eg.db (v3.18.0) using 2 terms defining stress: GO:0061621 (canonical glycolysis), GO:0034976 (response to endoplasmic reticulum stress). We found that both RGPs as well as glia cells scored high for the above terms. Therefore, we included 2 gene sets defining non-stressed cells: glia GO:0042063 (gliogenesis) and RGPs: GO:0021872 (forebrain generation of neurons), GO:0021873 (forebrain neuroblast division). Before Gruffi analysis cluster analysis was performed again: FindNeighbors (reduction = “pca”, dims = 1:30), FindClusters(resolution = 2).

#### Initial analysis 10X Flex mouse in vivo data

Initial filtering and identification of GFP expressing clusters of mouse in vivo data was performed as for organoid data with nCount_RNA < 20000. Clustering analysis: FindNeighbors (reduction = “pca”, dims = 1:15), FindClusters(resolution = 0.25).

#### Tissue origin of *Emx1*^*+*^/*Gfp*^*+*^ cells in organoids

Supplementary Figs. 2 and 3. This analysis was performed as for embryonic data with modifications (see below). We used the reference data prepared for the mouse in vivo analysis for label transfer: organoid D8 to reference e11.0, organoid D13 to reference e13.5, organoid D20 to reference e16.5, organoid D25 to reference e18.0. Identification of anchors and label transfer: FindTransferAnchors (normalization.method = “SCT”, reference.reduction = “pca”, dims = 1:20), TransferData (dims = 1:20). Note that during this analysis we identified that a group of D8 cells that was previously labelled as *Gfp*^+^ was actually *Gfp*-negative. To remove these cells we clustered D8 data with higher resolution, FindClusters (resolution = 0.6) and removed cluster 19 (333 cells) from downstream analyses.

#### Tissue origin of *Emx1*^*+*^/*Gfp*^*+*^ cells for mouse in vivo

Supplementary Figs. 4 and 5. Analysis of *Gfp* expression identified a number of cell clusters that did not express *Gfp*. To further investigate the origin of these cell clusters we compared our 10X FLEX scRNA-Seq data (before extraction of *Gfp* expressing cells) to a reference dataset that investigated multiple brain tissues during embryonic development^25^. We obtained read counts (in loom format) and additional annotation data from http://mousebrain.org/. We compared the E10 data from this study to reference E11.0 (SampleIDs: 10×40_3, 10×40_4, 10×40_5, 10×40_6), E13 from this study to reference E13.5 (SampleID: 10×14_4), E16 from this study to reference e16.5 (SampleID: 10×17_4) and P1 from this study to reference E18.0 (SampleID: 10×32_2). Cell cycle states were assigned using marker genes for G/M and S phase (https://github.com/hbc/tinyatlas/tree/master) and the CellCycleScoring function in Seurat. We normalized, scaled data and partially regressed out cell cycle effects using SCTransform with parameter: vars.to.regress = (S.score – G2M.score), separately for reference samples and samples from this study. We transferred annotations from reference to data from this study using FindTransferAnchors (normalization.method = “SCT”, reference.reduction = “pca”, dims = 1:15) and TransferData (dims = 1:15). Note that the Class ID was obtained from http://mousebrain.org/development/celltypes.html and was assigned via the ClusterName annotation given in the loom file. The Subclass ID originates directly from the loom file annotation. In order to simplify the annotation we identified the majority Subclass label in each seurat cluster (FindNeighbors (dims = 1:25), FindClusters(resolution = 1)) and assigned cell labels accordingly. For final presentation we combined Subclass and Class IDs and focused on annotations with >3% abundance.

#### Data integration and cell-type annotation

Figure 1 and Extended Data Figs. 2 and 14. To allow direct comparison between organoid and embryonic data we performed data integration. To this end, we utilized cluster similarity spectrum integration (CSS)^77^. CSS relies on cell clusters, identified in each sample, which it uses as an intrinsic reference for integration across samples. We merged mouse in vivo and organoid data (filtered for *Gfp* expressing cells as described above), joined all layers, and performed standard processing: NormalizeData, FindVariableFeatures (nfeatures = 5000), ScaleData and RunPCA (npcs = 30). Integration was performed with simspec (v0.0.0.9000) and cluster_sim_spectrum (label_tag = “group”, cluster_col = “seurat_clusters”, use_scale = F, var_genes = VariableFeatures([Seurat object])), where group defined either in vivo mouse or organoid. Cell-type identification (Fig. 1g, Extended Data Fig. 2a): dimensionality reduction was performed with RunUMAP (reduction = “css”, dims = 1:ncol(Embeddings ([Seurat object], “css”)), n.neighbors = 10, metric = “cosine”, n.components = 2, min.dist = 0.2). We defined clusters in the integrated data using FindNeighbors (reduction = “css”, dims = 1:ncol(Embeddings([Seurat object], “css”))) and FindClusters (resolution = 1). We manually associated seurat clusters to cell types using marker genes, seurat clusters, location on the UMAP and developmental timing of appearance: neurons: *Neurod2*, intermediate progenitors: *Eomes/Top2a*, RGP: *Hes5/Top2a*, astrocytes: *Aldh1l1*, oligodendrocytes: *Olig2*, olfactory bulb neuroblasts: *Dlx5*, Cajal–Retzius cells: *Nhlh2*, adult stem cells: mixed markers, linked to olfactory bulb neuroblasts, oligodendrocytes and astrocytes, appearing late in development. Down-sampling: owing to the use of multiple replicates, our dataset contained ~4× more organoid than embryo cells. To equalize this number for downstream analyses we down-sampled the data using SketchData (ncells = 35000, method = “LeverageScore”, sketched.assay = “sketch”). Such analysis identified 28,568 organoid cells, which we extracted and combined with the 24,942 embryonic cells. We used this down-sampled dataset for Extended Data Figs. 2 and 14, Fig. 1g–j and for marker gene analysis. Cell-type marker gene overlap was identified using FindAllMarkers (only.pos = T), separately for organoid and embryo data. We determined either the % overlap of the top 100 marker genes (Fig. 1h) or the intersection of the top 20 marker genes (Extended Data Fig. 2b) of each cell type for organoid/mouse in vivo data to prepare figures. *Emx1* expression (Extended Data Fig. 2c): we determined pseudobulk expression for each cell type using AggregateExpression and plotted normalised expression. Compare cell-type abundance dynamics during development between organoid and in vivo (Fig. 1i): For this analysis we transferred the cell-type labels from the embryo data from this study to reference datasets^25,26^. For^25^ data we used data from E11.0 (SampleID: 10×40_3, 10×40_4, 10×40_5, 10×40_6), E13.5 (SampleID: 10×14_4), E16.5 (SampleID: 10×17_4) and E18.0 (SampleID: 10×32_2). We extracted cells with the following subclass annotations: Cajal-Retzius, cortical hem, cortical or hippocampal glutamatergic, dorsal forebrain, forebrain, forebrain astrocyte, forebrain glutamatergic, neuronal intermediate progenitor, glioblast, mixed region astrocytes, committed oligodendrocyte precursor, oligodendrocyte precursor cell, oligodendrocyte. For^26^, we used data from E11 (GSM4635072), E13 (GSM4635074), E16 (GSM4635077), P1 (GSM4635080) and extracted cells with the following New_cellType annotations: apical progenitors, intermediate progenitors, immature neurons, Cajal–Retzius cells, migrating neurons, SCPN, CThPN, DL CPN, UL CPN, layer 4, NP, layer 6b, cycling glial cells, astrocytes and oligodendrocytes. For label transfer we normalized, scaled data and partially regressed out cell cycle effects using SCTransform as described before, separately for embryo data from this study and reference data^25,26^. We transferred annotations using FindTransferAnchors (normalization.method = “SCT”, reference.reduction = “pca”, dims = 1:15) and TransferData (dims = 1:15). We determined relative abundances of cells for each cell type at each developmental time points for embryo data from this study, and reference datasets^25,26^ and plotted the mean ± s.d. of these data points. For organoid data we plotted the mean (connected lines in the figure) as well as the actual relative abundances for the four biological replicates.

#### Matching in vivo mouse developmental age to organoid differentiation stage

Extended Data Figure 1e,f. We used transcriptional signature and relative abundance changes of key cell types to identify the matching embryonic age to our organoid data. (Extended Data Fig. 1e): For the heatmaps, we calculated pseudobulk expression levels of neurons and RGPs at the indicated time points for ref. ^25^ and our organoid data by averaging normalised expression. Next, we calculated all pairwise Pearson correlations within one cell type using all genes shared between datasets. We transformed correlations to *z*-scores within each reference time point. For heatmap plotting, we adjusted the data matrix so that the highest *z*-score at each organoid time point is 0 allowing direct comparison to the best match. (Extended Data Fig. 1f): we used two published datasets^25,26^. For data from ref. ^25^, we used data from E9–E18 in one-day intervals (10 samples). We extracted cells using a stepwise approach based on annotation in the loom file. (1) We extracted cells with a forebrain region annotation. (2) From that dataset, we extracted cells with the following subclass annotations: cortical hem, cortical or hippocampal glutamatergic, dorsal forebrain, forebrain, forebrain astrocyte, forebrain glutamatergic, neuronal intermediate progenitor, committed oligodendrocyte precursor, oligodendrocyte precursor cell, oligodendrocyte. (3) From this dataset we removed cells with the following class annotations: ependymal, glioblast, Cajal-Retzius. To match cell-type annotations across references we combined all class annotations containing astrocyte or oligodendrocyte to glia and renamed neuroblast to intermediate progenitors. For ref. ^26^, we used data from E10–E18 in one-day intervals, as well as P1 and P4 (11 samples). We extracted cells with the following New_cellType annotations: apical progenitors, intermediate progenitors, immature neurons, migrating_neurons, SCPN, CThPN, DL CPN, UL CPN, layer 4, NP, layer 6b, astrocytes, oligodendrocytes. To match cell-type annotations across references, we renamed apical progenitors to ‘radial glia’, combined astrocytes and oligodendrocytes to ‘glia’ and combined all other annotations (except for intermediate progenitors) to ‘neurons’. For organoid data, we used the annotation described above and combined astrocytes and oligodendrocytes to glia. Since we were interested in the trends of the abundance changes, we plotted reference data as a smoothed curve (method = ‘loess’, formula = ‘y ~ x’) with geom_smooth(fill = “grey70”, colour = “grey50”, level=0.9). Note that for this analysis only the metadata from the respective datasets were used, since no integration or label transfer was necessary.

#### MADM-CloneSeq data processing

We obtained MADM-CloneSeq data from 287 cells. Alignment was performed using STAR (v.2.7.9a)^78^ on GRCm39 and Gencode vM27 with STAR parameters:–outFilterMultimapNmax 1,–outSAMstrandField intronMotif,–outFilterIntronMotifs RemoveNoncanonical,–outFilterScoreMinOverLread 0.22,–outFilterMatchNminOverLread 0.22. Note that we only used the R2 reads from paired end sequencing for this analysis. Reads in exonic and intronic regions (Gencode vM27) were counted using the aligned bam files produced by STAR and summarizeOverlaps (GenomicAlignments v1.40.0, R v4.4.0) with singleEnd=TRUE, mode = “IntersectionNotEmpty”, ignore.strand = T, inter.feature = T parameters. We combined exonic and intronic reads and used the resulting expression matrix for CreateSeuratObject (min.cells = 3, min.features = 300), to prepare a Seurat object with 279 cells. We used annotated organoid data from D20 and D25 for normalized marker sum (NMS) analysis. We identified top 200 marker genes for each cell type (aNSC, astrocyte, Cajal–Retzius cell, neuron, intermediate progenitor, OBNB, oligodendrocyte and RGP). Such analysis identified 1270 unique genes (minimum of 154 genes per group). Next, we assigned each gene to one cell type through the highest expression. We calculated (1) the mean expression of the top 154 genes (highest expression) for each cell type to determine (2) one mean expression value for each cell type in the reference. For MADM-CloneSeq, mean marker gene expression was calculated as for the reference. The final NMS score was calculated as: mean cell-type marker gene expression in MADM-CloneSeq divided by the mean cell-type marker gene expression in reference. We determined the highest NMS score for non-projection neuron cell types (non-neuron NMS). Heatmap and clustering analysis of NMS scores identified 3 clusters of PatchSeq cells with either high neuron NMS score (largest cluster, 249 cells) or high score in any other cell type (2 smaller clusters with 13 and 17 cells). We assumed that cells with a high score in a non-neuron cell type are either patched cells that were not neurons or that had a high amount of contaminating RNA from surrounding, non-neuronal cells. In both cases such cells would confound downstream analysis and were removed. We found that non neuronal cells were characterized by a ratio of neuron NMS/non-NMS of 1.2 (95th percentile: 1.195). We used a cutoff of 0.3 neuron NMS score to identify cells with a poor sequencing quality, to reduce noise. High quality neurons were defined as cells with neuron NMS / non-NMS > 1.2 and neuron NMS score > 0.3 (215 cells). Note that remaining 64 cells consisted of 31 low quality cells (highest NMS score <0.31) and 33 high quality cells (highest NMS score > 0.31). As such ~87% of high quality cells (215/248 cells) are of neuronal identity. For data integration we identified Seurat clusters in MADM-CloneSeq cells. Note that library preparation was performed in 3 batches (plates) and batch effects were considered in downstream analyses: SCTransform (vars.to.regress = c(“plate”, “NMS_Neurons”), RunPCA (assay = “SCT”), FindNeighbors (dims = 1:12), FindClusters (resolution = 2.5).

#### Neuron analysis

Figure 4b–h and Extended Data Figs. 12 and 13. Data integration: we prepared reference data^26^ (E12, E13, E14, E15, E16, E17, E18_S3 and P1_S1) by extracting cells from the neuronal lineage (New_cellType annotations: Apical progenitors, Intermediate progenitors, Immature neurons, Migrating neurons, SCPN, CThPN, DL CPN, UL CPN, Layer 4, NP, Layer 6b) and processed each sample separately: NormalizeData, FindVariableFeatures(nfeatures = 3000), ScaleData, RunPCA, FindNeighbors(dims = 1:15), FindClusters(resolution = 0.8). We extracted organoid neuronal lineage cells (RGP, intermediate progenitor, neurons) from D13, D20 and D25 and processed each sample (D13, D20, D25) separately: NormalizeData, FindVariableFeatures (nfeatures = 5000), ScaleData, RunPCA (npcs = 30), FindNeighbors (dims = 1:15), FindClusters (resolution = 0.8). Finally we combined all datasets (reference, organoid, PatchSeq) and performed integration: NormalizeData, FindVariableFeatures (nfeatures = 3000), ScaleData, RunPCA (npcs = 30), cluster_sim_spectrum (spectrum_type = “corr_ztransform”, label_tag = “mergeGroup”, cluster_col = “seurat_clusters”). The mergeGroup was either developmental age (reference, organoid) or plate (MADM-CloneSeq). Reference UMAP and annotation: To prepare a common reference UMAP we extracted reference cells from the integrated data and performed dimensionality reduction in integrated (css) space: RunUMAP (reduction = “css”, dims = 1:ncol(Embeddings([Seurat object], “css”)), n.neighbors = 30, metric = “euclidean”, n.components = 3, min.dist = 0.3, return.model = T, seed.use = 2401, reduction.name = “umap_3d”). We annotated cell types using reference annotation and marker gene expression via Seurat clusters: FindNeighbors (dims = 1:150, reduction = “css”), FindClusters (resolution = 1.6). Annotating organoid data: we projected organoid cells onto the reference UMAP: ProjectUMAP(query = [organoid Seurat object], query.reduction = “css”, query.dims = 1:ncol(Embeddings([organoid Seurat object], “css”)), reference = [reference Seurat object], reference.reduction = “css”, reference.dims = 1:ncol(Embeddings([reference Seurat object], “css”)), reduction.name = “umap_3d”, reduction.model = “umap_3d”) and identified nearest reference cells for each organoid cell in 3D UMAP space using nn2 function (RANN package v2.6.1). This analysis identified the 10 closest reference cells for each organoid cell. We then identified the reference Seurat clusters of each of 10 closest reference cells and assigned the cluster name with the highest frequency. Note that for 86.5% of organoid cells >=80% of reference annotations were matching. For ~1.9% of organoid cells, no reference annotation could be assigned as 2 or more reference annotations had the same frequency. Cell-type annotation for organoid data was assigned using Seurat clusters as for the reference data. Cortical layer marker analysis (Extended Data Fig. 12a-b): we calculated DEGs between upper layer and lower layer cells using FindMarkers for reference and organoid data separately and plotted the average log_2_ fold change as indicated in the figure legend. MADM-Clone-Seq analysis: we removed 2 cells with uncertain clone assignment for further analyses. We projected MADM-CloneSeq cells on the reference 3D UMAP using ProjectUMAP (query = [MADM-CloneSeq Seurat object], query.reduction = “css”, query.dims = 1:ncol(Embeddings([MADM-CloneSeq Seurat object], “css”)), reference = [reference Seurat object], reference.reduction = “css”, reference.dims = 1:ncol(Embeddings([reference Seurat object], “css”)), reduction.name = “umap_3d”, reduction.model = “umap_3d”). We determined 10 nearest neighbour reference cells in 3D UMAP space for each MADM-CloneSeq cell using the nn2 function. Cell-type annotation: We identified the reference cell type for each of the 10 closest reference cells and assigned the cell type with the highest frequency. Note that for 86.4% of MADM-CloneSeq cells ≥80% of nearest neighbour reference annotations were matching. We removed 6 (~2.8%) MADM-CloneSeq cells as no reference annotation could be assigned as 2 or more reference annotations had the same frequency, resulting in 207 cells. We additionally excluded 12 clones with only 1 informative cell, resulting in a final dataset of 195 cells, which was used for all downstream analyses unless indicated otherwise. Note that this analysis assigned MADM-CloneSeq cells only to upper/deep layer annotations despite inclusion of all reference cell types in the analysis. Nearest neighbour distance Extended Data Fig. 13b: distance to the nearest neighbour (from each small neurogenic to the closest small neurogenic, from each asymmetric neurogenic to the closest asymmetric neurogenic, from each small neurogenic to the closest asymmetric neurogenic and from each asymmetric neurogenic to the closest small neurogenic cell) was calculated in 3D UMAP space using the nn2 function. Cortical layer markers (Extended Data Fig. 13c): We determined differential expression of upper/deep layer cells in the 207 MADM-CloneSeq cells with unambiguous cell-type assignment, for the top 200 DEGs identified in upper/deep layer comparison in the reference and plotted key marker genes identified in Extended Data Fig. 12a. Pseudotime annotation (Fig. 4e-h): we determined pseudotime for reference data using the monocle3 (v1.3.7) pipeline: as.cell_data_set, cluster_cells, learn_graph, order_cells. For each MADM-CloneSeq cell we determined the mean pseudotime of the 10 nearest neighbour reference cells.

#### RGP analysis

Extended Data Figure 14. We extracted RGP cells (as defined in Fig. 1) from organoid and mouse in vivo data and determined DEGs for comparisons D8/E10, D13/E13 using FindMarkers. DEGs were defined as p_val_adj <0.01 and avg_log2FC < -1 (embryo specific)/avg_log2FC > 1 (organoid specific). Gene Ontology term enrichment was identified using clusterProfiler (v4.10.0)^79^ and org.Mm.eg.db (v.3.18.0) via enrichGO(OrgDb = org.Mm.eg.db, ont = “BP”, readable = T). Significance score was determined as the negative log_10_ of the adjusted *P* value.

#### Developmental trajectory analysis

Figure 4j–l and Extended Data Fig. 15. To determine developmental trajectories we used slingshot (v2.10.0)^80^: getLineages(clusterLabels = [seurat_clusters], dist.method = “slingshot”, omega = T, omega_scale = 2). We used a combination of variable (provided in respective figure legend) and fixed parameters for dimensionality reduction and clustering: RunUMAP(reduction = “css”, dims = 1:ncol(Embeddings([organoid neuron Seurat object], “css”)), n.neighbors = [variable], metric = “euclidean”, min.dist = [variable], return.model = T), FindClusters(resolution = [variable]). To focus analysis and display we combined the central portion of neuronal cells to one Seurat cluster (Nr. 99).

### Quantification and statistical analysis

Data for all studies were stored and processed using Microsoft Excel (Microsoft). Statistical analyses were performed using Graphpad Prism software v10.2.2 (Dotmatics). All data were expressed as mean ± s.e.m. or 95% confidence intervals, as indicated in the figure legend, where *n* represents the number of clones, unless otherwise stated. Data were checked for normality using the Shapiro-Wilk test, and analysed with appropriate parametric or non-parametric tests. To test for statistically significant differences of clone size over time, we implemented either the Kruskal-Wallis test, followed by multiple comparisons tests for statistical significance between two time points or count data were modelled using a generalized linear mixed model (GLMM) implemented in R (glmmTMB, v1.1.14), assuming either a negative binomial (asymmetric and proliferative clones) or a Conway–Maxwell–Poisson error distribution (Small neurogenic clones), depending on which best fit the data. The fixed effect was age and the random effect was a random intercept for batch. The overall effect of age on counts was evaluated with a likelihood ratio test comparing the full model (counts ~ age + (1|batch)) to a reduced model without the age term (counts ~ 1 + (1|batch)). The Chi-squared test was used to compare relative abundance between samples. All explanations of *n* numbers, statistical tests used and *P* values have been provided throughout the results and the Methods section, in the corresponding figure legends and in Source Data as well as Supplementary Tables 2 and 3.

### Reporting summary

Further information on research design is available in the Nature Portfolio Reporting Summary linked to this article.

## Online content

Any methods, additional references, Nature Portfolio reporting summaries, source data, extended data, supplementary information, acknowledgements, peer review information; details of author contributions and competing interests; and statements of data and code availability are available at https://doi.org/10.1038/s41586-026-10916-7.

## Supplementary information

Supplementary Information This file contains Supplementary Figs. 1–8.

Reporting Summary

Supplementary Table 1 Custom FLEX barcode probe sequences for detection of *Gfp* and *tdT* expression.

Supplementary Table 2 Table with MADM clone data considering all batches and different lines.

Supplementary Table 3 Table with relevant source data for supplementary figures.

Peer Review File

## Source data

Source Data Figs. 2–4 and Extended Data Figs. 4, 5, 7–11 and 13

## Data Availability

All data generated and analysed in this study are included in the paper, source data and/or Supplementary Tables 2 and 3. Raw sequencing data have been deposited with Gene Expression Omnibus (GEO) accession number GSE327470. Source data are provided with this paper.

## Code availability

All scripts used to prepare data and figures for this manuscript are accessible on GitHub at https://github.com/fpauler/Temporal-Uncoupling-of-Radial-Glia-Lineage-Progression-in-Cortical-Organoid.
