# Supplementary material for: Temporal uncoupling of radial glia lineage progression in cortical organoids
Source: Nature. 2026 Aug 12;658(8136):716–24. doi: 10.1038/s41586-026-10916-7 (PMC13668663; doi:10.1038/s41586-026-10916-7)
Supplement: Supplementary file 1 [file 41586_2026_10916_MOESM1_ESM.pdf]

nature portfolio

https://doi.org/10.1038/s41586-026-10916-7

Supplementary information

Temporal uncoupling of radial glia lineage progression in cortical organoids

In the format provided by the authors and unedited

Supplementary Figures

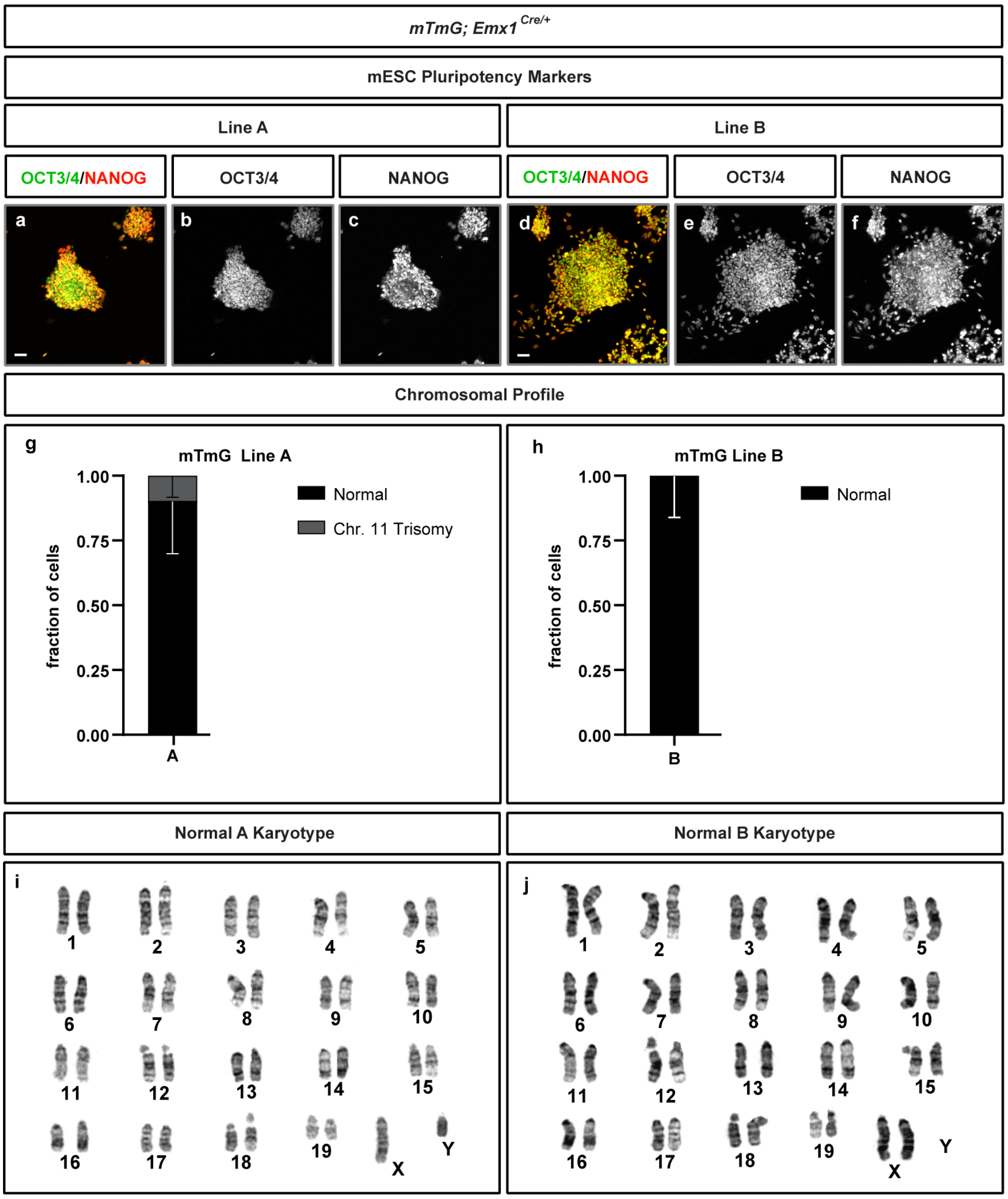

Supplementary Figure 1. Expression of pluripotency markers and chromosome profiles in novel *mTmG* mouse embryonic stem cell lines.

a-c and d-f, Representative 10x confocal images of mESC colonies from 2 different lines. Colonies from lines A, and B were stained for OCT3/4 (green, white; **a**, **b**, **d**, **e**) and NANOG (red, white; **a**, **c**, **d**, **f**), respectively.

g-j, Genomic integrity was assayed by performing a G-banding karyotype analysis for each of the cell lines. Representative images of the results from the G-banding karyotype (**i**, **j**). Karyotyping showed that the vast majority of all cell lines had an unaltered genome (**g and h**), We only found chromosome 11 trisomy, a chromosomal aberration commonly found in mESCs [79], in two individual cells sampled from line A. n = 20 cells for each line. Error bars indicate 95% confidence interval.

Scale bar: 50 µm.

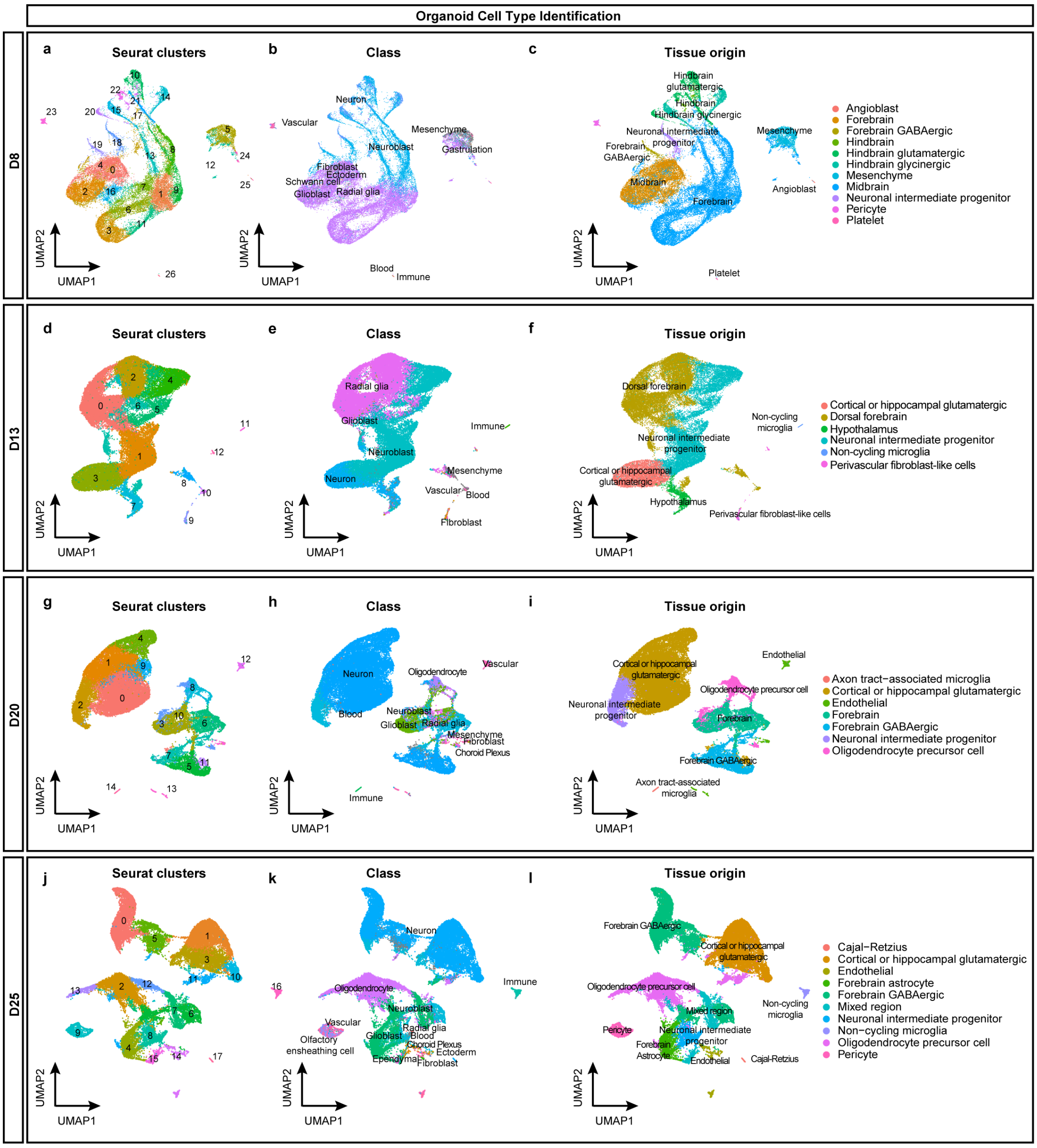

Supplementary Figure 2. Cell type identification in organoid scRNA-Seq data sets.
a-l, UMAPs derived from scRNAseq data from *mTmG*$^{+/-}$;*Emx1*$^{Cre/+}$ organoids across distinct differentiation time points, D8 (**a-c**), D13 (**d-f**), D20 (**g-i**), D25 (**j-l**) with different colors and labels indicating distinct modalities. Unbiased Seurat cluster analysis (**a, d, g, j**), label transfer from reference [25] Class annotation indicate main cell types (**b, e, h, k**); and label transfer from reference [25] SubclassID annotation indicate main tissue origin (**c, f, i, l**). Note that each differentiation time point was analyzed using 4 biological replicates, which were combined for the analysis.

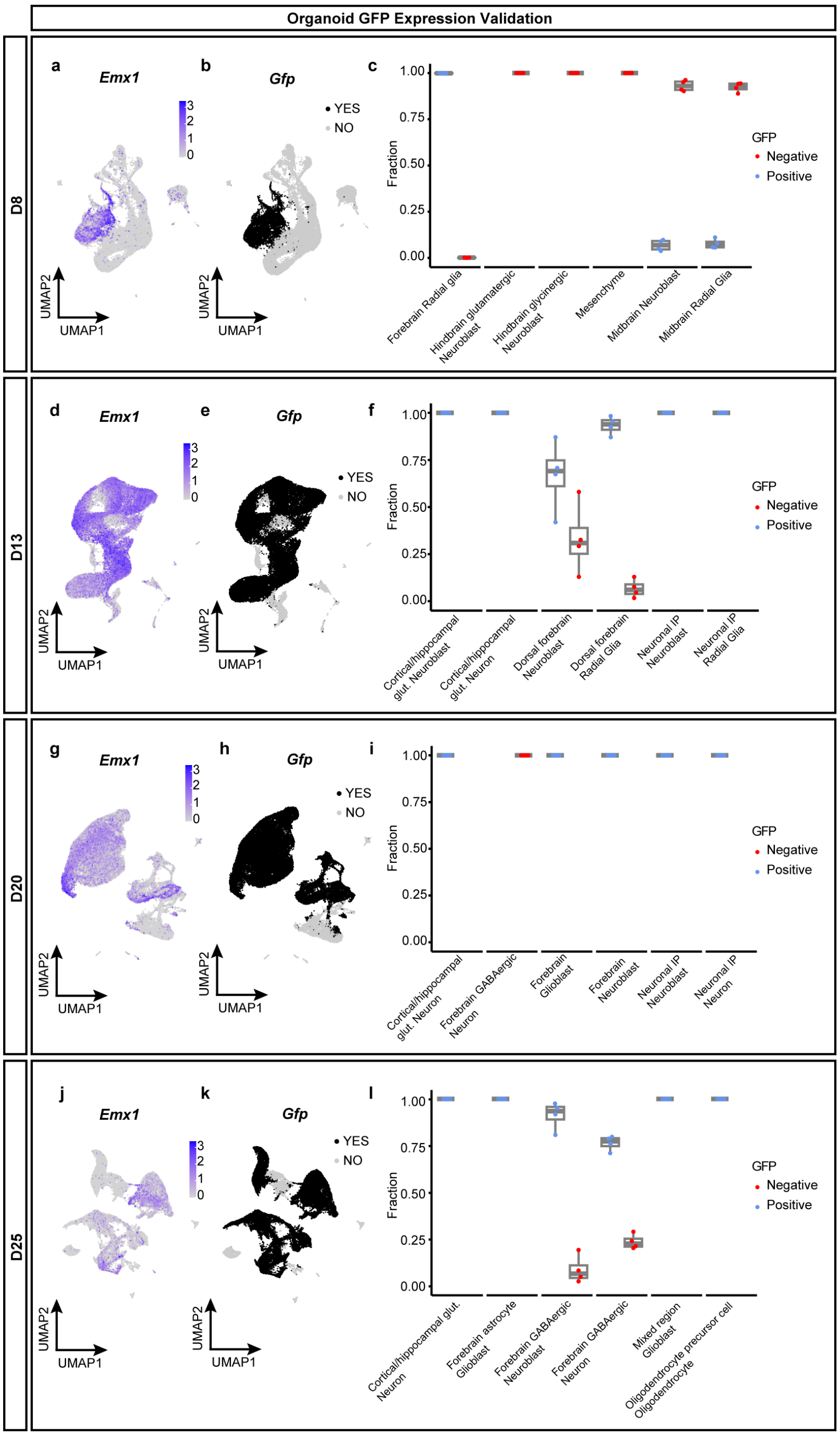

Supplementary Figure 3. *Emx1* and *Gfp* expression analysis in organoid scRNA-Seq data sets. a-l, UMAPs derived from scRNAseq data from *mTmG*$^{+/-}$;*Emx1*$^{Cre/+}$ organoids across distinct differentiation time points: D8 (**a-b**), D13 (**d-e**), D20 (**g-h**), D25 (**j-k**), indicating *Emx1* expression (**a, d, g, j**) and *Gfp* annotation (**b, e, h, k**); and quantification of the fraction of *Gfp* positive/negative cells in each of the resulting groups (**c, f, i, l**), n = 4 organoids per time point. The centre line indicates the median, the box limits show the 25–75% interquartile range (IQR), whiskers extend to include the furthest data points within 1.5x the IQR from box edges, and data points outside this range are represented as dots.. Note that the low number of *Emx1*$^{+}$/*Gfp*$^{+}$ expressing cells at D8 (**a-c**) correlates with incomplete forebrain patterning. From D13 onwards most cell types were *Gfp*$^{+}$. At D20 we detected a "Forebrain GABAergic neuron" type that was *Gfp*$^{-}$. Such a cell type is reminiscent of *in vivo* GABAergic interneurons that originate from *Emx1*$^{-}$ progenitors in the MGE, and later migrate tangentially into the cortex. At D25 *Gfp*$^{+}$ "Forebrain GABAergic neuron" and "Forebrain GABAergic neuroblast" cell types emerged. These data correlate well with the appearance of *Gfp*$^{+}$ olfactory bulb

neuroblasts (OBNB) *in vivo* that originate from *Emx1*$^{+}$ RGPs, and show many characteristics of GABAergic interneurons (see also Supplementary Fig. 4, 5). Since the reference dataset [25] which we utilized did not discriminate between GABAergic interneurons and OBNB, the observed “Forebrain GABAergic neuron” and to a lesser extent “Forebrain GABAergic neuroblast” in our dataset contain both cell types and thus a mix of *Gfp*$^{-}$ and *Gfp*$^{+}$ cells.

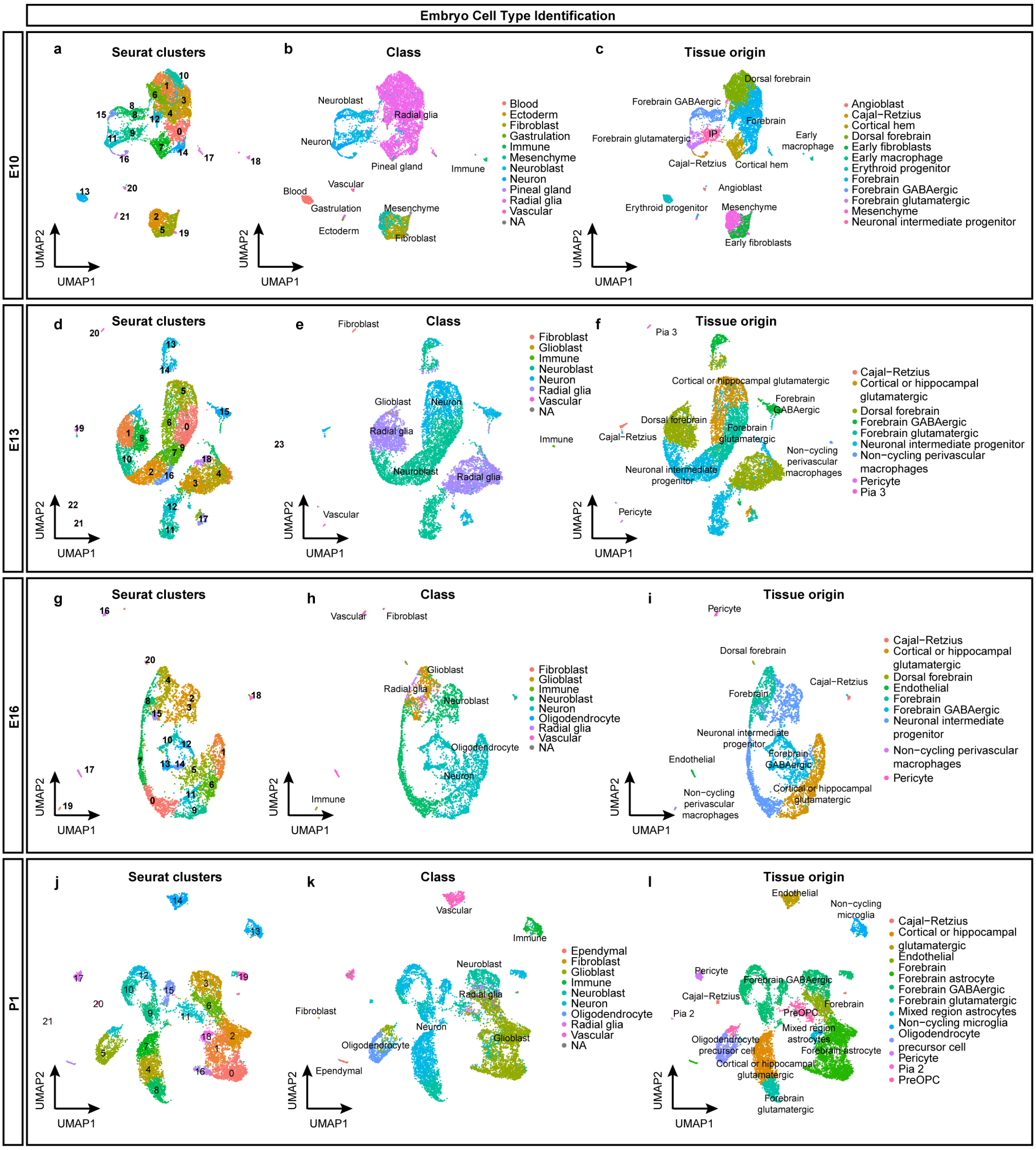

Supplementary Figure 4. Cell type identification and tissue annotation in embryo scRNA-Seq data sets.

a-l, UMAPs derived from scRNAseq data from *mTmG*$^{+/-}$;*Emx1*$^{Cre/+}$ embryos across distinct developmental time points: E10 (**a-c**), E13 (**d-f**), E16 (**g-i**), P1 (**j-l**) with different colors and labels indicating distinct modalities. Unbiased Seurat cluster analysis (**a, d, g, j**); label transfer from reference [25] Class annotation indicating main cell types (**b, e, h, k**); and label transfer from reference [25] SubclassID annotation indicating main tissue origin (**c, f, i, l**). n = 40, 30, 10, and 4 brains pooled from 8, 4, 2, and 2 litters for E10, E13, E16 and P1 timepoints, respectively.

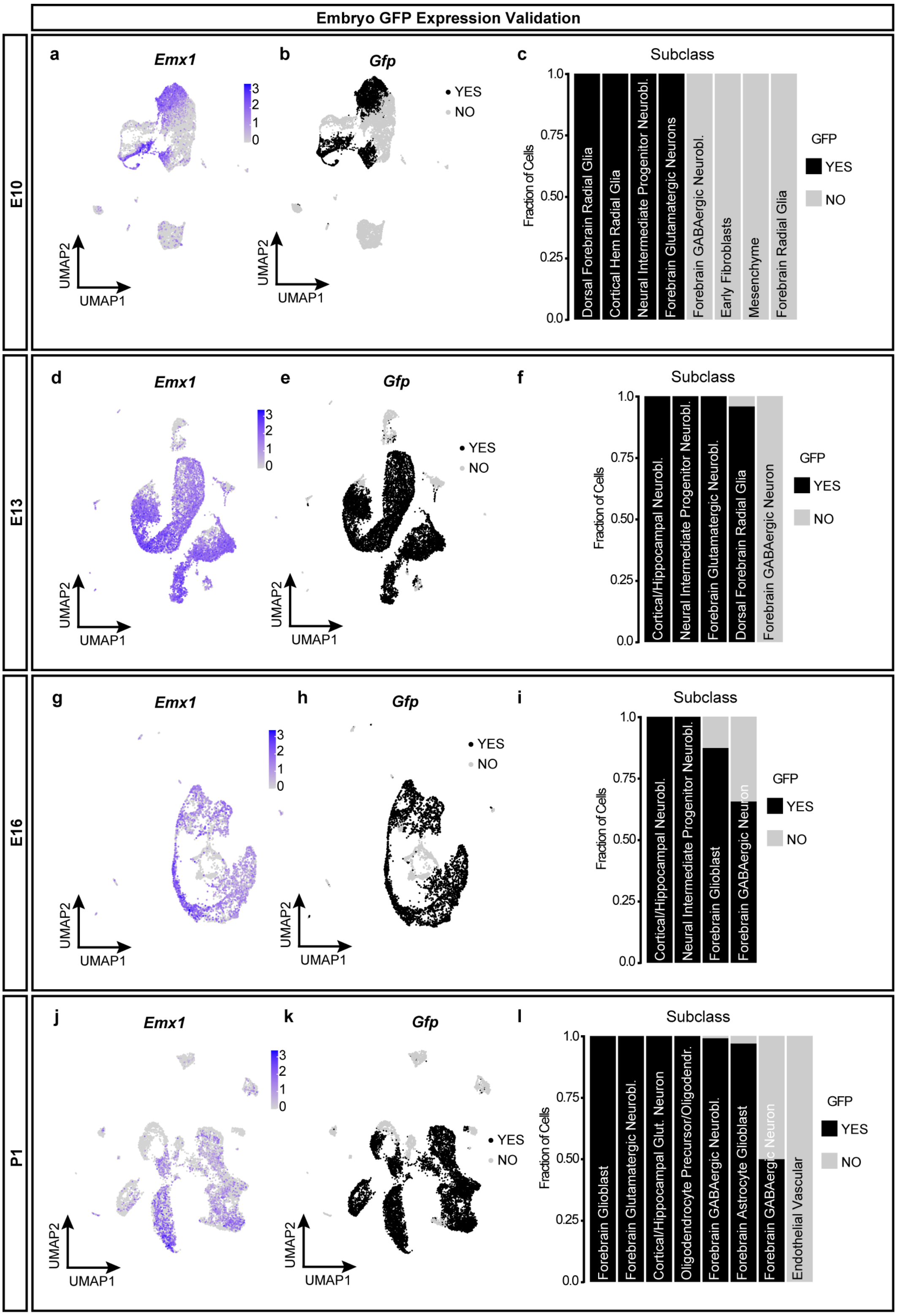

Supplementary Figure 5. *Emx1* and *Gfp* expression analysis in embryo scRNA-Seq data sets.
a-l, UMAPs derived from scRNAseq data from *mTmG*$^{+/-}$;*Emx1*$^{Cre/+}$ embryos across distinct developmental time points: E10 (**a-b**), E13 (**d-e**), E16 (**g-h**), P0 (**j-k**) indicating *Emx1* expression (**a, d, g, j**) and GFP annotation (**b, e, h, k**). Embryonic cells were grouped by combining the cell type/tissue annotations as shown in Supplementary Fig. 4, and the fraction of *Gfp* positive/negative cells was quantified in each of the resulting groups (**c, f, i, l**). Note that the low number of *Emx1*/*Gfp* expressing cells at E10 (**a-c**) correlates with a higher amount of non-forebrain tissue similar to our observation in organoid (see Supplementary Fig. 3).

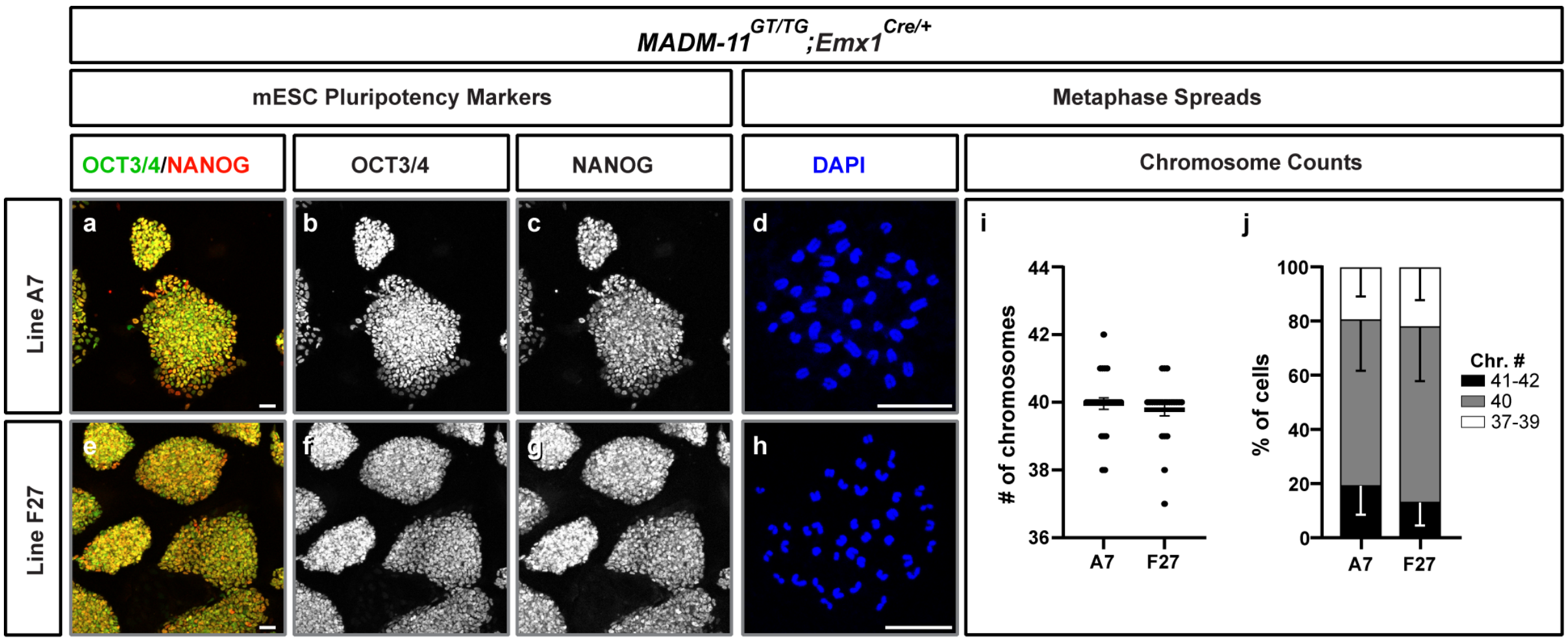

Supplementary Figure 6. Expression of pluripotency markers and genomic integrity in novel *MADM-11*$^{GT/TG}$;*Emx1*$^{Cre/+}$ mouse embryonic stem cell lines.

a-c and e-g, Representative 10x confocal images of mESC colonies from 2 lines (Line A7 and Line F27) stained for OCT3/4 (**a, b, e, f**) and NANOG (**a, c, e, g**), respectively.

d, h, i, j, Genomic integrity was assayed by performing a metaphase spread for each of the cell lines. DNA was labeled using DAPI (**d and h**). Quantification of chromosome numbers showed that the vast majority of cells from both lines had 40 chromosomes (**i and j**), with a minority of sampled cells carrying abnormal numbers of chromosomes. n = 26 and 23 cells for lines A7 and F27, respectively. (**i**) Data shown are mean ± S.E.M.

(**j**) Error bars indicate 95% confidence interval.

Scale bar: 50 µm (**a-c and e-g**), 20 µm (**d and h**).

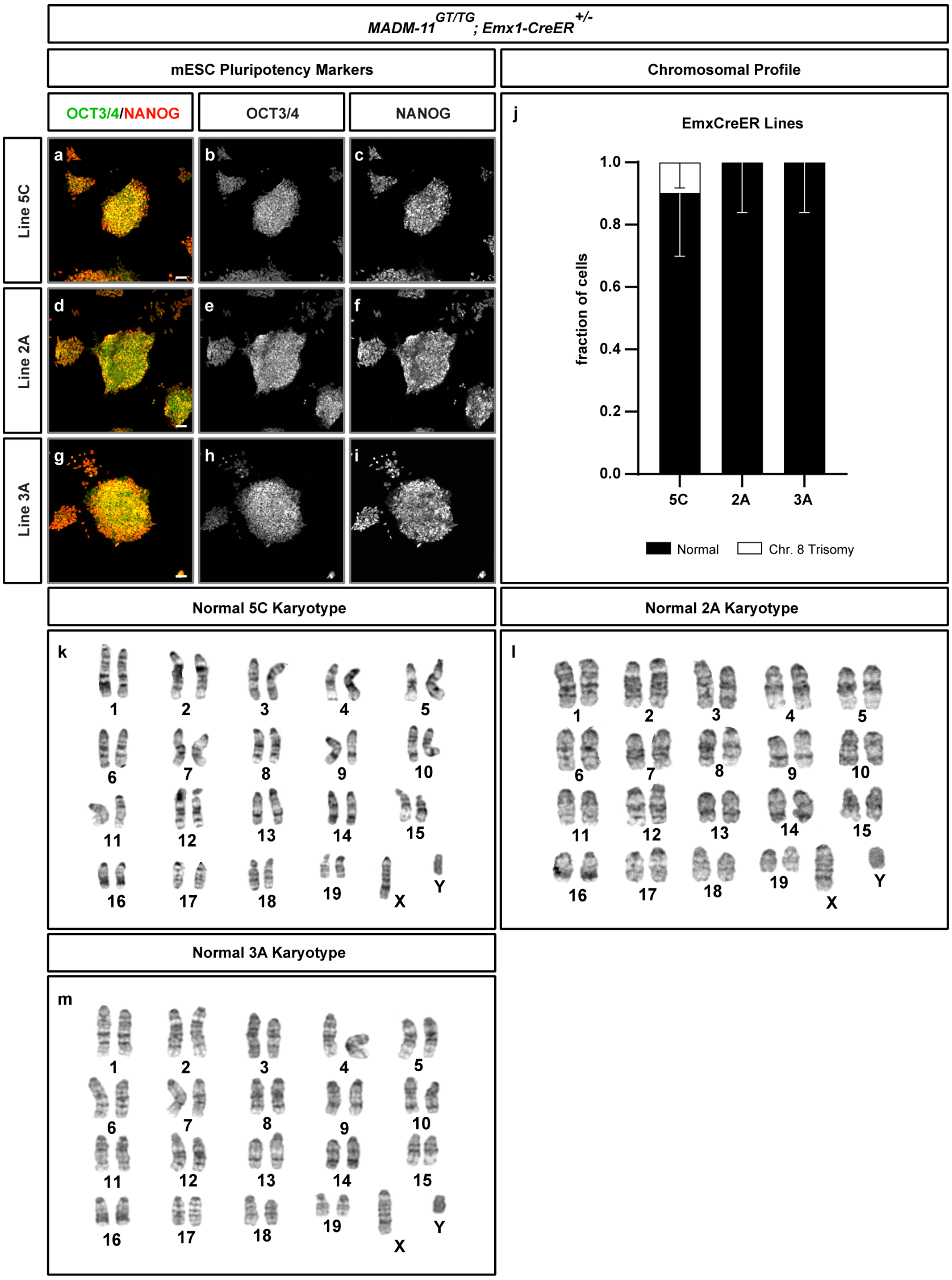

Supplementary Figure 7. Expression of pluripotency markers and chromosome profiles in novel *MADM-11*$^{GT/TG}$*;Emx1-CreER*$^{+/-}$ mouse embryonic stem cell lines.

a-c, d-f, and g-i, Representative 10x confocal images of mESC colonies from 3 lines (Line 5C, Line 2A, and Line 3A). Colonies from lines 5C, 2A, and 3A were stained for OCT3/4 (**a, b, d, e, g, h**) and NANOG (**a, c, d, f, g, i**), respectively. Stem cell marker labeling was performed once per line.

j, Genomic integrity was assayed by performing a G-banding karyotype for each of the cell lines. Karyotyping showed that the majority of all cell lines had an unaltered genome, with a minority of sampled cells (2 individual cells from Line 5C) carrying some anomalies in chromosome 8 which are commonly found in mESCs [79,80]. n = 20 cells for each line. Error bars indicate 95% confidence interval.

k-m, Representative images of the results from the G-banding karyotype.

Scale bar: 50 μm.

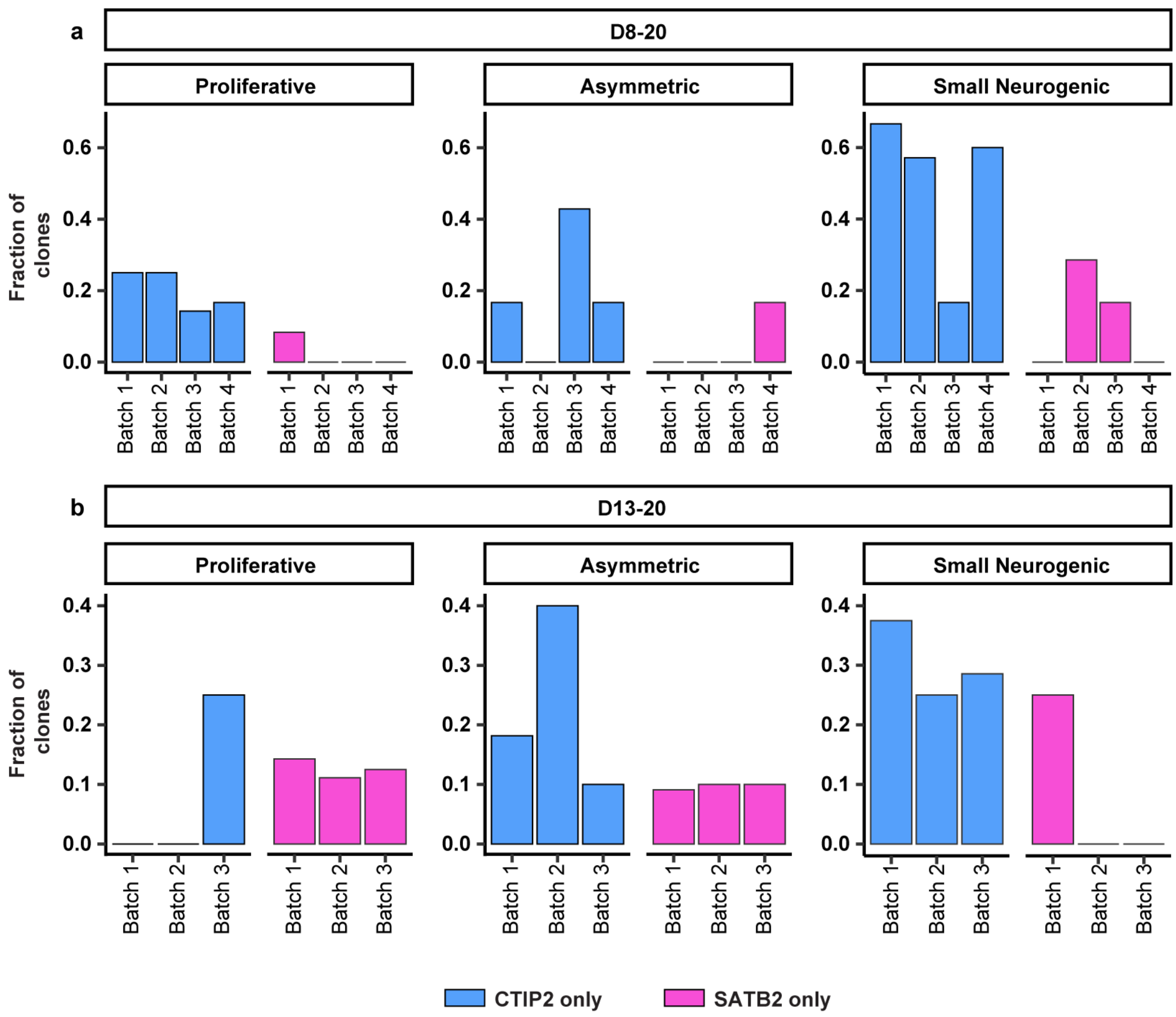

Supplementary Figure 8. Analysis of RGP lineage bias across distinct batches of organoids.
a-b, Fraction of proliferative, asymmetric neurogenic, and small neurogenic MADM clones with lineage restriction towards CTIP2^{+} (blue bars) or SATB2^{+} (magenta bars), induced at D8 (**a**, n=37, Asym. n=28, Small n=22) or D13 (**b**, n=24, asym. n=31, small n=23) and analyzed at D20; across distinct batches of organoids. Two-way ANOVA: D8-20 CTIP2 vs SATB2 p = 0.00034 ***, D13-20 CTIP2 vs SATB2 p = 0.18, ns.
