# Supplementary material for: Temporal uncoupling of radial glia lineage progression in cortical organoids
Source: Nature. 2026 Aug 12;658(8136):716–24. doi: 10.1038/s41586-026-10916-7 (PMC13668663; doi:10.1038/s41586-026-10916-7)
Supplement: Supplementary file 2 [file 41586_2026_10916_MOESM2_ESM.pdf]

nature portfolio

Corresponding author(s): Simon Hippenmeyer

Last updated by author(s): Jun 30, 2026

Reporting Summary

Nature Portfolio wishes to improve the reproducibility of the work that we publish. This form provides structure for consistency and transparency in reporting. For further information on Nature Portfolio policies, see our Editorial Policies and the Editorial Policy Checklist.

Statistics

For all statistical analyses, confirm that the following items are present in the figure legend, table legend, main text, or Methods section.

n/a	Confirmed	
☐	☒	The exact sample size (*n*) for each experimental group/condition, given as a discrete number and unit of measurement
☐	☒	A statement on whether measurements were taken from distinct samples or whether the same sample was measured repeatedly
☐	☒	The statistical test(s) used AND whether they are one- or two-sided<br>*Only common tests should be described solely by name; describe more complex techniques in the Methods section.*
☐	☒	A description of all covariates tested
☐	☒	A description of any assumptions or corrections, such as tests of normality and adjustment for multiple comparisons
☐	☒	A full description of the statistical parameters including central tendency (e.g. means) or other basic estimates (e.g. regression coefficient) AND variation (e.g. standard deviation) or associated estimates of uncertainty (e.g. confidence intervals)
☐	☒	For null hypothesis testing, the test statistic (e.g. *F*, *t*, *r*) with confidence intervals, effect sizes, degrees of freedom and *P* value noted<br>*Give P values as exact values whenever suitable.*
☒	☐	For Bayesian analysis, information on the choice of priors and Markov chain Monte Carlo settings
☒	☐	For hierarchical and complex designs, identification of the appropriate level for tests and full reporting of outcomes
☐	☒	Estimates of effect sizes (e.g. Cohen's *d*, Pearson's *r*), indicating how they were calculated

Our web collection on statistics for biologists contains articles on many of the points above.

Software and code

Policy information about availability of computer code

Data collection	Images of cells for metaphase chromosome spread and cortical organoids were processed using Zeiss ZEN Blue 2.3 and 2.6 software (Zeiss).
Data analysis	Data visualization and statistical analyses were performed using Graphpad Prism 10.2.2 (Dotmatics). Data were stored and processed using Excel 2016 (Microsoft). Confocal images were processed and analyzed using ImageJ 1.54p (Fiji), IMARIS File Converter (Oxford Instruments), IMARIS Stitcher (Oxford Instruments), IMARIS v9.9.1 (Oxford Instruments). Bioinformatic analyses were performed using R v4.3.2/4.4.0 (R-Project), cellranger v8.0.0 (10X Genomics), STAR v.2.7.9a (DOI: 10.1093/bioinformatics/bts635), R packages: gruffi v1.5.5 (DOI: 10.15252/embj.2022111118), Seurat v5.0.1 (DOI: 10.1038/s41587-023-01767-y), simspec v0.0.0.9000 (DOI: 10.1186/s13059-020-02147-4), slingshot v2.10.0 (DOI: 10.1186/s12864-018-4772-0), monocle3 v1.3.7, RANN v2.6.1, clusterProfiler v4.10.0 (10.1016/j.xinn.2021.100141), org.Mm.eg.db v.3.18.0, GenomicAlignments v1.40.0. Scripts used are available at: https://github.com/fpauler/Temporal-Uncoupling-of-Radial-Glia-Lineage-Progression-in-Cortical-Organoid

For manuscripts utilizing custom algorithms or software that are central to the research but not yet described in published literature, software must be made available to editors and reviewers. We strongly encourage code deposition in a community repository (e.g. GitHub). See the Nature Portfolio guidelines for submitting code & software for further information.

Data

Policy information about availability of data

All manuscripts must include a data availability statement. This statement should provide the following information, where applicable:

- Accession codes, unique identifiers, or web links for publicly available datasets
- A description of any restrictions on data availability
- For clinical datasets or third party data, please ensure that the statement adheres to our policy

All data generated and analyzed in this study are included in the paper, Source Data and/or Supplementary Table 2. Raw sequencing data has been deposited with Gene Expression Omnibus (GEO) accession number GSE327470 (https://www.ncbi.nlm.nih.gov/geo/query/acc.cgi?acc=GSE327470).

Research involving human participants, their data, or biological material

Policy information about studies with human participants or human data. See also policy information about sex, gender (identity/presentation), and sexual orientation and race, ethnicity and racism.

Reporting on sex and gender	No data or biological material from humans was used in this study.
Reporting on race, ethnicity, or other socially relevant groupings	n/a
Population characteristics	n/a
Recruitment	n/a
Ethics oversight	n/a

Note that full information on the approval of the study protocol must also be provided in the manuscript.

Field-specific reporting

Please select the one below that is the best fit for your research. If you are not sure, read the appropriate sections before making your selection.

☒ Life sciences ☐ Behavioural & social sciences ☐ Ecological, evolutionary & environmental sciences

For a reference copy of the document with all sections, see nature.com/documents/nr-reporting-summary-flat.pdf

Life sciences study design

All studies must disclose on these points even when the disclosure is negative.

Sample size	Sample sizes were not predetermined in this study. Variability among replicates was used to determine appropriate sample size, with a minimum of 3 biological replicates for histology experiments. Sample sizes were also based on previously reported data from our group (Gao et al Cell 2014, Beattie et al Neuron 2017, Amberg et al Sci. Adv. 2022). Male and female mice were used.
Data exclusions	No data were excluded in histology experiments. Standard scRNA-Seq workflow includes removal of low quality cells based on the following criteria: Organoid: nFeature_RNA > 1000 & nFeature_RNA < 8000 & nCount_RNA < 40000 & percent.mt < 5, Embryo: as for organoid data with nCount_RNA < 20000
Replication	All histological experiments in organoid had more than or equal to 3 independent batches, organoid sequencing experiments included 4 pools of organoids from distinct batches and 2 cell lines per time point, while in vivo sequencing experiments included x1 biological replicate per time point.
Randomization	Organoids used in this study were selected randomly. Mice were excluded only if they were not of the correct genotype, no other exclusionary criteria was applied. The dataset was monitored for batch effects.
Blinding	Analyses were performed without blinding. There are only one experimental organoid group and one experimental mouse group hence blinding was not possible.

Reporting for specific materials, systems and methods

We require information from authors about some types of materials, experimental systems and methods used in many studies. Here, indicate whether each material, system or method listed is relevant to your study. If you are not sure if a list item applies to your research, read the appropriate section before selecting a response.

Materials & experimental systems

n/a	Involved in the study
☐	☒ Antibodies
☐	☒ Eukaryotic cell lines
☒	☐ Palaeontology and archaeology
☐	☒ Animals and other organisms
☒	☐ Clinical data
☒	☐ Dual use research of concern
☒	☐ Plants

Methods

n/a	Involved in the study
☒	☐ ChIP-seq
☒	☐ Flow cytometry
☒	☐ MRI-based neuroimaging

Antibodies

Antibodies used	Primary antibodies and dilution factors included chicken anti-GFP (Aves, GFP1020, 1:1000), goat anti-tdTomato (SICgen, ab8181-200, 1:1000), rabbit anti-PAX6 (Cell Signaling, 60433S, 1:500), rat anti-TBR2 (Thermo Fisher Scientific, 14-4875-82, 1:400), rat anti-CTIP2 (ab18465, 1:400), mouse anti-SATB2 (Abcam, ab51502, 1:200), rabbit anti-NANOG (Abcam, ab80892, 1:500), mouse anti-OCT3/4 (Santa Cruz, sc5279, 1:200), rabbit anti-GFAP (DAKO, Z0334 1:1000), rabbit anti-NEUROD2 (ab109406, 1:200) and rabbit anti-CASPASE3 (Cell Signaling, 9661S). Secondary antibodies and dilution factors included donkey anti-chicken-Alexa488 (Jackson Immuno, 703-545-155, 1:1000), donkey anti-goat-Alexa568 (Invitrogen, A11057, 1:1000), donkey anti-goat-CY3 (Jackson Immuno, 705-165-147, 1:1000), donkey anti-rat-Alexa594 (Life Technologies, A21205, 1:1000), donkey anti-mouse-Alexa647 (Life Technologies, A32787, 1:1000) and donkey anti-rabbit-Alexa647 (Life Technologies, A31573, 1:1000).
Validation	All antibodies were tested and validated by manufacturers to be reactive to the mouse epitope of the target protein. Working concentrations of antibodies were based on the manufacturers' recommendations. These were then titrated based on optimization experiments performed on in-house tissue to determine the best concentration for the optimal signal-to-noise ratio.

Eukaryotic cell lines

Policy information about cell lines and Sex and Gender in Research

Cell line source(s)	All mouse embryonic stem cells used in this study were derived from timed mating of mice via blastocyst collection at embryonic day E3.5.
Authentication	Expression of stem cell markers and absence of expression of differentiation markers was tested by antibody staining / immunohistochemistry. Karyotyping was performed to validate integrity of newly-derived stem cell lines.
Mycoplasma contamination	mESCs were routinely tested for mycoplasma using the LookOut Mycoplasma PCR Detection Kit (Sigma)
Commonly misidentified lines<br>(See ICLAC register)	No commonly misidentified cell line was used in this study.

Animals and other research organisms

Policy information about studies involving animals; ARRIVE guidelines recommended for reporting animal research, and Sex and Gender in Research

Laboratory animals	Mouse lines used in this study:<br>Mouse: MADM-11-GT - ISTA or The Jackson Laboratory IMSR_JAX: 013749 (Hippenmeyer et al. 2010)<br>Mouse: MADM-11-TG- ISTA or The Jackson Laboratory IMSR_JAX: 013751 (Hippenmeyer et al. 2010)<br>Mouse: Emx1-Cre - ISTA or The Jackson Laboratory RRID:IMSR_JAX:005628 (Gorski et al. 2002)<br>Mouse: Emx1-CreER - ISTA or The Jackson Laboratory RRID:IMSR_JAX:027784 (Kessaris et al. 2006)<br>Mouse: mTmG reporter - ISTA or The Jackson Laboratory RRID:IMSR_JAX:007676 (Muzumdar et al. 2007)<br><br>All mouse lines used in this study were kept in a mixed C57/Bl6 and CD1 genetic background. Mice were used at an age range from 2 – 8 months for general breeding, from 2 – 4 months (females) for collecting blastocysts, and at P21 for clonal analysis experiments in vivo.
Wild animals	This study did not involve wild animals.
Reporting on sex	Both male and female littermates of the desired genotypes were used randomly. No difference between sex was observed.
Field-collected samples	No field collection was carried out in this study.
Ethics oversight	All animal procedures were approved by the Austrian Federal Ministry of Women, Science and Research in accordance with the Austrian and European Union animal law (license number: BMWF-66.018/0007-II/3b/2012; BMWFW-66.018/0006-WF/V/3b/2017; GZ: 2020-0.579.989 and GZ: 2025-0.597.515) Experimental mice were bred and maintained according to regulations approved by institutional animal care and use committee, institutional ethics committee and the guidelines of the preclinical facility (PCF) at ISTA.

Note that full information on the approval of the study protocol must also be provided in the manuscript.

Plants

Seed stocks	No plant material was used in this stdy.
Novel plant genotypes	n/a
Authentication	n/a
