# Supplementary material for: Temporal uncoupling of radial glia lineage progression in cortical organoids
Source: Nature. 2026 Aug 12;658(8136):716–24. doi: 10.1038/s41586-026-10916-7 (PMC13668663; doi:10.1038/s41586-026-10916-7)
Supplement: Supplementary file 3 [file 41586_2026_10916_MOESM3_ESM.pdf]

BC001	CCTTGGCACCCGAGAATTCCAAGGGTGTCGCCCTCGAACTTCACCT	GFP
BC001	/5Phos/CGGCGCGGGTCTTGTAGTTGCCGTCACGCGGTTAGCACGTAN NACTTTAGGCGGTCCTAGCAA	GFP
BC001	CCTTGGCACCCGAGAATTCCAATGGTGCGCTCCTGGACGTAGCCTT	GFP
BC001	/5Phos/CGGGCATGGCGGACTTGAAGAAGTCACGCGGTTAGCACGTAN NACTTTAGGCGGTCCTAGCAA	GFP
BC001	CCTTGGCACCCGAGAATTCCAGGTAGTGGTCGGCGAGCTGCACGCT	GFP
BC001	/5Phos/GCCGTCCTCGATGTTGTGGCGGATCACGCGGTTAGCACGTANN ACTTTAGGCGGTCCTAGCAA	GFP
BC001	CCTTGGCACCCGAGAATTCCATAGTCCTCGTTGTGGGAGGTGATGT	tdT
BC001	/5Phos/CCAGCTTGGTGTCCACGTAGTAGTAACGCGGTTAGCACGTANN ACTTTAGGCGGTCCTAGCAA	tdT
BC001	CCTTGGCACCCGAGAATTCCAAACTCGTGGCCGTTCATGGAGCCCT	tdT
BC001	/5Phos/CCATGCGCACCTTGAAGCGCATGAAACGCGGTTAGCACGTAN NACTTTAGGCGGTCCTAGCAA	tdT
BC001	CCTTGGCACCCGAGAATTCCATTGTAATCGGGGATGTCGGCGGGGT	tdT
BC001	/5Phos/GCTTCACGTACGCCTTGGAGCCGTAACGCGGTTAGCACGTAN NACTTTAGGCGGTCCTAGCAA	tdT
BC002	CCTTGGCACCCGAGAATTCCAAGGGTGTCGCCCTCGAACTTCACCT	GFP
BC002	/5Phos/CGGCGCGGGTCTTGTAGTTGCCGTCACGCGGTTAGCACGTAN NAACGGGAACGGTCCTAGCAA	GFP
BC002	CCTTGGCACCCGAGAATTCCAATGGTGCGCTCCTGGACGTAGCCTT	GFP
BC002	/5Phos/CGGGCATGGCGGACTTGAAGAAGTCACGCGGTTAGCACGTAN NAACGGGAACGGTCCTAGCAA	GFP
BC002	CCTTGGCACCCGAGAATTCCAGGTAGTGGTCGGCGAGCTGCACGCT	GFP
BC002	/5Phos/GCCGTCCTCGATGTTGTGGCGGATCACGCGGTTAGCACGTANN AACGGGAACGGTCCTAGCAA	GFP
BC002	CCTTGGCACCCGAGAATTCCATAGTCCTCGTTGTGGGAGGTGATGT	tdT
BC002	/5Phos/CCAGCTTGGTGTCCACGTAGTAGTAACGCGGTTAGCACGTANN AACGGGAACGGTCCTAGCAA	tdT
BC002	CCTTGGCACCCGAGAATTCCAAACTCGTGGCCGTTCATGGAGCCCT	tdT
BC002	/5Phos/CCATGCGCACCTTGAAGCGCATGAAACGCGGTTAGCACGTAN NAACGGGAACGGTCCTAGCAA	tdT
BC002	CCTTGGCACCCGAGAATTCCATTGTAATCGGGGATGTCGGCGGGGT	tdT
BC002	/5Phos/GCTTCACGTACGCCTTGGAGCCGTAACGCGGTTAGCACGTAN NAACGGGAACGGTCCTAGCAA	tdT
BC003	CCTTGGCACCCGAGAATTCCAAGGGTGTCGCCCTCGAACTTCACCT	GFP
BC003	/5Phos/CGGCGCGGGTCTTGTAGTTGCCGTCACGCGGTTAGCACGTAN NAGTAGGCTCGGTCCTAGCAA	GFP
BC003	CCTTGGCACCCGAGAATTCCAATGGTGCGCTCCTGGACGTAGCCTT	GFP
BC003	/5Phos/CGGGCATGGCGGACTTGAAGAAGTCACGCGGTTAGCACGTAN NAGTAGGCTCGGTCCTAGCAA	GFP
BC003	CCTTGGCACCCGAGAATTCCAGGTAGTGGTCGGCGAGCTGCACGCT	GFP
BC003	/5Phos/GCCGTCCTCGATGTTGTGGCGGATCACGCGGTTAGCACGTANN AGTAGGCTCGGTCCTAGCAA	GFP
BC003	CCTTGGCACCCGAGAATTCCATAGTCCTCGTTGTGGGAGGTGATGT	tdT

BC003	/5Phos/CCAGCTTGGTGTCCACGTAGTAGTAACGCGGTTAGCACGTANNAGTAGGCTCGGTCCTAGCAA	tdT
BC003	CCTTGGCACCCGAGAATTCCAAACTCGTGGCCGTTCATGGAGCCCT	tdT
BC003	/5Phos/CCATGCGCACCTTGAAGCGCATGAAACGCGGTTAGCACGTANNAGTAGGCTCGGTCCTAGCAA	tdT
BC003	CCTTGGCACCCGAGAATTCCATTGTAATCGGGGATGTCGGCGGGGT	tdT
BC003	/5Phos/GCTTCACGTACGCCTTGGAGCCGTAACGCGGTTAGCACGTANNAGTAGGCTCGGTCCTAGCAA	tdT
BC004	CCTTGGCACCCGAGAATTCCAAGGGTGTCGCCCTCGAACTTCACCT	GFP
BC004	/5Phos/CGGCGCGGGTCTTGTAGTTGCCGTCACGCGGTTAGCACGTANNATGTTGACCGGTCCTAGCAA	GFP
BC004	CCTTGGCACCCGAGAATTCCAATGGTGCGCTCCTGGACGTAGCCTT	GFP
BC004	/5Phos/CGGGCATGGCGGACTTGAAGAAGTCACGCGGTTAGCACGTANNATGTTGACCGGTCCTAGCAA	GFP
BC004	CCTTGGCACCCGAGAATTCCAGGTAGTGGTCGGCGAGCTGCACGCT	GFP
BC004	/5Phos/GCCGTCCTCGATGTTGTGGCGGATCACGCGGTTAGCACGTANNATGTTGACCGGTCCTAGCAA	GFP
BC004	CCTTGGCACCCGAGAATTCCATAGTCCTCGTTGTGGGAGGTGATGT	tdT
BC004	/5Phos/CCAGCTTGGTGTCCACGTAGTAGTAACGCGGTTAGCACGTANNATGTTGACCGGTCCTAGCAA	tdT
BC004	CCTTGGCACCCGAGAATTCCAAACTCGTGGCCGTTCATGGAGCCCT	tdT
BC004	/5Phos/CCATGCGCACCTTGAAGCGCATGAAACGCGGTTAGCACGTANNATGTTGACCGGTCCTAGCAA	tdT
BC004	CCTTGGCACCCGAGAATTCCATTGTAATCGGGGATGTCGGCGGGGT	tdT
BC004	/5Phos/GCTTCACGTACGCCTTGGAGCCGTAACGCGGTTAGCACGTANNATGTTGACCGGTCCTAGCAA	tdT
