# Supplementary material for: Temporal uncoupling of radial glia lineage progression in cortical organoids
Source: Nature. 2026 Aug 12;658(8136):716–24. doi: 10.1038/s41586-026-10916-7 (PMC13668663; doi:10.1038/s41586-026-10916-7)
Supplement: Supplementary file 4 [file 41586_2026_10916_MOESM6_ESM.pdf]

Peer Review File

Temporal Uncoupling of Radial Glia Lineage Progression in Cortical Organoid

Corresponding Author: Professor Simon Hippenmeyer

Any redactions in this file are there to maintain patient confidentiality, the confidentiality of unpublished data, or to remove third-party material.

This file contains all reviewer reports in order by version, followed by all author rebuttals in order by version.

Attachments originally included by the reviewers as part of their assessment can be found at the end of this file.

Version 0:

Reviewer comments:

Referee #1

(Remarks to the Author)
Summary: Stouffer et al. present an extensive clonal analysis of radial glial progenitor (RGP) cell behavior in mouse cortical organoids using Mosaic Analysis with Double Markers (MADM). The authors establish a MADM system in embryonic stem cells and generate cortical organoids to track RGP lineage progression over time. Their data suggest that, unlike the in vivo environment where RGPs undergo a stereotyped and temporally ordered progression from proliferative to neurogenic to gliogenic fates, RGPs in organoids show greater equipotency, reduced cell-type diversity, and increased lineage restriction. The authors conclude that self-organization alone is insufficient to recapitulate the in vivo lineage behavior of RGPs.
Major Comments:
1. Lineage Marker Specificity:
o The use of the Emx1-CreER driver raises concerns. While it is intended to mark RGPs, Emx1 is also expressed in intermediate progenitors (IPs) and neurons, both newborn and mature. This could confound lineage tracing results and lead to erroneous interpretations of clone origin and architecture. The authors should explicitly validate the specificity of Emx1 expression in their ESC-derived system.
2. Clone Architecture Interpretation in Organoids:
o The classification of clone types (proliferative, asymmetric neurogenic, small neurogenic) relies on spatial organization and ratios of red/green cells, criteria validated in vivo where spatial layering allows neuron identification. In organoids, such laminar organization is often absent. Without reliable positional cues, defining clone types based solely on cell number ratios could be misleading.
o The authors make an assumption that red/green labeled cells in organoids represent neurons, unlike the cortical plate context in vivo. This assumption should be addressed explicitly and experiments performed to prove if this is actually valid.
3. Lineage Restriction and Trajectory Analysis:
o The observation of increased lineage restriction in organoids (~32% of clones biased toward either SCPN or CPN fates) contrasts with in vivo (~3%). However, the conclusion that this represents a fundamental divergence requires caution. The observed restriction may be due to technical artifacts such as leaky Cre expression, stochastic behavior in vitro, or even metabolic/culture-related stresses.
o The trajectory analysis shows a single, unitary transcriptional path connecting RGPs to both SCPN and CPN types. This appears inconsistent with the clonal restriction data and should be reconciled. Are the authors possibly over-interpreting a relatively smooth transcriptional trajectory as uniform potency?
o The trajectory analysis could benefit from clearer explanation and robustness checks. Are there multiple trajectories or forks within the iN cluster? How robust are these to clustering and dimensionality reduction parameters?
4. Cell Death Considerations:
o The manuscript addresses whether clone shrinkage might result from cell death. Caspase-3 staining indicates <6% cell death, comparable to in vivo. This is a valuable control, but the interpretation assumes this rate is uniformly distributed. A deeper analysis (e.g., spatial localization or lineage-stage dependence of apoptosis) would strengthen the conclusion.
5. Gliogenesis:
o The authors mention that gliogenesis occurs after neurogenesis, mirroring in vivo behavior. However, this point is

underdeveloped. How does this temporally ordered fate switch occur in the absence of stereotyped neurogenesis? Further discussion is warranted.
6. Role of Extrinsic Cues:
o A central claim is that non-cell-autonomous, niche-derived cues are necessary for faithful RGP lineage progression. This is plausible and aligns with prior findings (e.g., Nowakowski et al., Bhaduri et al.). However, the paper would benefit from a more detailed discussion of what these cues might be: cell-cell signaling, mechanical forces, metabolic environment? Specific hypotheses or proposed future directions could enrich the discussion.
7. Title Tone:
o The current title is arguably provocative. While the data do show limitations of self-organization, the phrase "self-organization is insufficient" implies a broader generalization that might not apply across all contexts. A more tempered title might reflect the nuance of the findings.
Minor Comments:
• Typo noted on page 12: "13 CTIP2" should likely read "2 CTIP2+ cells."
• Figure 4b is confusing and not consistent with 4a. The authors should clarify the figure legend and graphical elements.
• The statistical approach to clone size comparisons (pairwise testing) may obscure meaningful trends; trendline overlays or regression models might be more appropriate.
• Authors should cite Nowakowski (human RG lineage studies) and Bhaduri (organoid maturation defects) to situate their findings in broader literature.
This study addresses an important question in stem cell and developmental neurobiology. The experimental design is thoughtful and leverages the power of MADM for lineage tracing. The findings are intriguing, suggesting that organoids, while powerful, may not fully recapitulate in vivo lineage dynamics. However, interpretation of the data, particularly regarding clone type assignment and transcriptional trajectories, requires caution. Strengthening the discussion of technical limitations, validating key assumptions, and integrating relevant prior work would significantly enhance the manuscript.

Referee #2

(Remarks to the Author)
The study from Stouffer and colleagues presents an innovative application of the MADM (Mosaic Analysis with Double Markers) system in mouse embryonic stem cell-derived cortical organoids to analyze radial glial progenitor (RGP) lineage progression. The authors directly compare in vitro lineage progression in organoids to the in vivo developmental process, providing a valuable framework to understand the advantages and limitations of using organoids as models for neurodevelopmental studies.
The key findings identified by the authors indicate that, unlike the in vivo condition where RGPs exhibit strict temporal lineage progression, RGPs in cortical organoids display a high level of equipotency with increased lineage restriction. This results in a diminished diversity of cortical projection neuron types. Furthermore, despite a seemingly uniform transcriptional profile and unitary lineage trajectory, RGP behavior in organoids differs significantly from the in vivo condition, confirming that non-cell-autonomous cues absent in self-organizing systems play an essential role in normal RGP lineage progression and cortical cell-type diversity.

It is to be noted that this is the first direct comparative analysis of lineage progression in mouse organoids versus in vivo cortical development, which provides crucial insights into how organoid models may recapitulate or diverge from in vivo processes. The study builds upon prior work using MADM for lineage tracing but extends it into the domain of organoid research. This comparison highlights fundamental differences in developmental constraints and plasticity between these systems, contributing substantially to our understanding of the fidelity of organoid models as tools for studying cortical development and disease modeling.
The findings are undoubetly significant for the field, as organoids are extensively used to study neurodevelopment in other species too. Understanding the limitations and advantages of this system may be critical for accurate data interpretation.

The experimental design is well-structured, and the application of MADM in cortical organoids is powerful. An important strength is the use of early passage stocks (P8-15) for organoid differentiation, which is crucial given that prolonged passaging can affect differentiation potential. However, no quality control (QC) analysis of the generated ESC lines is included. This is a critical omission, as evaluating pluripotency and DNA integrity is essential to ensure that newly derived lines maintain their differentiation potential. Including such data would significantly strengthen the study. In addition, it is important to define how robust and reproducible is the used protocol to generate organoids.
The MADM lineage tracing approach is a well-established technique, and the authors - expert pioneers of this method - have applied it effectively. The methodology is well described, and the decision to use three independent ESC lines with three differentiations (batches) ensures reproducibility and reliability of results. However, a clearer description of batch variability and statistical approaches used to account for potential batch effects would be beneficial.
The single-cell RNA sequencing analysis is also well executed and described in the Methods section. However, visualization approaches such as UMAP split by differentiation day or colored by sample would enhance the interpretability of the data.
The statistical tests chosen throughout the study appear appropriate for the data presented. However, there are several areas where the reporting of statistical methodology, sample size evaluation, and data presentation require clarification. The assessment of sample size, particularly the number of independent batches, ESC lines, and organoids analyzed, remains unclear. The figure legends primarily state the number of clones but do not specify whether statistical analyses were performed at the level of clones, batch, or cell line. This is particularly relevant when comparing organoid data to in vivo findings, as batch effects and inter-organoid or line variability may influence the observed trends. A more explicit breakdown of the number of independent differentiations, along with statistical considerations for batch and cell line variations, would strengthen the robustness of the conclusions.

The conclusions drawn from the study are generally well-supported by the data presented. The study provides strong evidence that cortical organoid models recapitulate some aspects of in vivo cortical development but diverge in lineage progression and progenitor behavior. The observed increase in lineage restriction and equipotency in organoid-derived RGPs is an important finding, emphasizing the need for caution in interpreting organoid-based neurodevelopmental studies. However, additional clarification regarding variability and statistical treatment would further reinforce the reliability of the conclusions.

1. The validation of the MADM paradigm in the organoid at the population level is unclear (Extended Data Fig. 2). The data show tdT/GFP cells present in organoids and IF of markers. Does "validation at the population level" mean double staining of the reporter and cell population markers? Please clarify. In addition, quantitative analysis should be included, such as determining the percentage of tdT/GFP-positive cells that co-express specific markers at each time point. Statistical quantification should be provided to support this validation.
2. Since new original ESC lines are generated, all necessary controls should be provided. It would be useful to include G-banding karyotyping to assess the chromosomal integrity of the ESC lines. Additionally, conducting immunostaining or qPCR for self-renewal and pluripotency markers (e.g., OCT4, SOX2, and lineage markers in embryoid bodies) would strengthen the characterization of these ESC lines.
3. The experimental timeline and developmental alignment between organoids and in vivo models require clarification. Is this experimental timeline comparable to in vivo MADM analysis from Gao et al. 2014 (E10-13 injection, analysis at P7-30) and the in vivo data presented in this work (E10 -> P21, Extended Fig. 4)? Are D8-D9 truly consecutive time points at the same pace as E10-E11 in vivo? How was organoid differentiation aligned with in vivo cortical development? A clear temporal comparison between organoid differentiation and in vivo cortical development, leveraging single-cell profiling of both organoids and publicly available in vivo samples, is recommended. also, can the authors confidently say that the organoid generation is as precise and consistent across organoids/batches/lines as the one in vivo between embryos?
4. Two key differences were found between cortical organoids and in vivo tissues.
- The presence of small neurogenic clones is particularly interesting, as it represents a potential difference from in vivo conditions. Could this be due to artificial conditions or missing signaling cues in organoids?can the authors discuss these distinctions.
- Increased lineage restriction (clones containing mainly Ctip2+ cells or Satb2+ cells) was observed in cortical organoids (30%) compared to in vivo tissues (3%).

It would be valuable to investigate small neurogenic clones and the increased lineage restriction further by performing lineage tracing experiments using additional approaches, such as genetic barcoding, to allow analysis at the single-cell transcriptome level. This could help clarify whether these differences are due to intrinsic properties of the organoid system or whether they reflect a developmental delay or differences in culture conditions.
5. Batch, cell line, and organoid-specific variations should be considered in the analysis. Throughout the manuscript, it is unclear whether batch, cell line, and organoid-specific variations were accounted for. We strongly suggest performing an additional analysis to assess how these factors influence clone size and other phenotypic traits. This could be done by including information on the number of batches used and conducting batch or line comparisons during statistical analysis. The authors should also include this information in the plots to show variability across lines and batches and clarify how these factors influence outcomes.
6. The output of the analysis comparing MADM clone architecture at different time points (D15 and D20) should be presented clearly in the figure. Additional details, such as bar graphs or heatmaps, should be included to show the relative proportions of clone types at each induction time point. A summary table or statistical comparison across time points would also help clarify the results.
7. Variability and statistical analysis of CTIP2+ and SATB2+ clones should be provided. The authors should ensure that variability is shown in the analysis of CTIP2+ and SATB2+ clones by including error bars (e.g., standard deviation or SEM) in the figure. Additionally, the statistical tests used to compare the groups should be clearly stated, and appropriate statistical methods (e.g., ANOVA, t-tests) should be employed to test for significant differences. Providing raw data or supplemental information on the statistical analysis would improve transparency.
8. The low percentage of GFP+ cells in D8 single-cell experiments contradicts in vivo Emx1 expression. The authors report a low percentage of GFP+ cells in D8 samples from the single-cell experiment. This contradicts in vivo Emx1 expression. Could this be due to delayed recombination or misalignment between organoid differentiation and in vivo development? Additionally, why do the authors rely on GFP recombination to sort Emx1-positive cells instead of using Emx1 transcript expression directly? To address this, we suggest investigating recombination efficiency at earlier stages (e.g., day 6 or 7) to determine whether delayed recombination could explain the low GFP+ cell numbers. A temporal comparison between in vivo Emx1 expression and organoid differentiation should also be performed to check for alignment in timing.
9. Separation between SCPN and CPN clusters in scRNA-seq data is unclear. There is no clear separation between SCPN and CPN clusters. A marker table or annotation dot plot is missing. To improve clarity in the scRNA-seq analysis, we suggest including a marker table with key genes expressed in SCPN and CPN clusters. It may also be worthwhile to reassess the clustering parameters used in the scRNA-seq analysis. Alternative methods, such as adjusting clustering resolution or applying different dimensionality reduction techniques, may help achieve better separation between SCPN and CPN populations.
10. The inferred trajectory in scRNA-seq analysis appears over-branched. The trajectory inference appears overly complex, which may obscure meaningful developmental relationships. Adjusting trajectory inference parameters or using alternative methods may provide a better representation of the differentiation trajectory. Additionally, pseudotime scoring should be included to further assess differentiation processes.

Referee #3

(Remarks to the Author)
I co-reviewed this manuscript with one of the reviewers who provided the listed reports.

Referee #4

(Remarks to the Author)
In this study, Stouffer and colleagues explore the progression of radial glial progenitors (RGPs) in vitro using mouse self-organizing cerebral organoids. RGPs, known to drive cortical cell lineage progression, are typically studied in vivo in murine models, where this process is believe to be largely driven by cell-intrinsic factors. However, the surrounding stem cell niche also plays a crucial role in facilitating this lineage progression. Previous studies have shown that RGPs cultured in 2D systems exhibit limited lineage development, suggesting that non-cell-autonomous factors are essential but so far poorly understood. Using mosaic analysis with double-marker-based lineage tracing in organoids, they found that RGPs in these 3D cultures showed division mode defects, restricted lineage progression, and reduced cell-type diversity in projection neuron clones, as compared to in vivo conditions. While organoids maintained the overall genetic program of RGPs, the absence of environmental niche cues in the 3D culture system limited their ability to fully replicate natural lineage progression and neural diversity.
This study underscores the importance of considering the limitations of organoid models when interpreting neural development data. The experimental design is solid, and the manuscript is well-written for a broad audience, but additional analyses and expanded discussion would further strengthen the work. Overall, it serves as a valuable reminder to approach organoid-based findings with caution, especially when exploring the complexities of neural cell lineage progression.

Main comments

> Does neural cell lineage progression vary across presumptive cortical areas in the Emx1 MADM mouse model? If so, could inadequate establishment of arealization in cerebral organoids hinder proper lineage progression within these self-organizing structures?

> The final concentration of 4-OHT used to generate a single clone per organoid is reduced by almost half specifically at the point when a significant decrease in observed cell clone size is noted (see Materials and Methods and Figure 1G). What is the scientific rationale for this concentration adjustment, and does it pose a risk of introducing technical bias?

> Figure 1J. The observation of an unexpectedly high proportion of small neurogenic clones in organoids is noteworthy. By examining clones five days earlier, the authors suggest that these small neurogenic clones are not artifacts resulting from 'depleted' proliferative clones. They show that activated caspase immunoreactivity remains low and stable throughout the culture period. However, since apoptosis represents only one form of cell death, what about necrosis (and possibly ferroptosis), which is usually known to accumulate, particularly in the central core of organoids? Additionally, is there any correlation between organoid size/rosette enrichment and the ability to generate the three distinct clone architectures?

> Figure 2. Lineage-restricted clones are frequently relatively small, particularly for CTIP2+ neurons. To what extent could this be influenced by cell death or the relative proportion of radial glial cells (RGCs, or apical progenitors) versus intermediate progenitors (BPs), which are primarily responsible for generating callosal projection neurons (CPNs) in vivo in mice?

> Figures 3 and S8. The scRNA-seq analysis reveals that some Emx1-derived cell clusters, specifically clusters 8, 10, and to a lesser extent 6, include Dlx1+ interneurons—an observation not observed in similar in vivo analyses in mice. What could explain this discrepancy? Might this indicate an artifact, suggesting additional compromised lineage progression in vitro? This finding is significant and warrants further discussion. Additionally, it has important implications for the interpretation of Figure 3F, where data were extracted from the original dataset to focus on the 'neural developmental lineage.'

>Figure S8. It would be helpful to provide a clear correspondence between cluster numbers and cell types, perhaps in the form of a table listing key marker genes used for the classification of major cell types. Additionally, what is the in vivo equivalent of aNSCs in the mouse cortex, and how distinct are these cells from radial glial progenitors (RGPs)? There appears to be no reference to these cells in the main text. Regarding the unitary trajectory from progenitors to projection neurons, the developmental trajectory depicted by Monocle3 (Figure 3I) aligns with expectations based on in vivo or in situ analyses of the mouse cortex. However, since this observation relies on a 'cell context-extracted' dataset, is the 'projection neuron trajectory' still preserved when considering a broader context that includes additional cell types, as shown in Figures S8 (where two connected lineages may be guessed)?

> In the discussion: The authors note the importance of comparing the transcriptional profiles of RGPs in vivo and in cerebral organoids. Performing this comparison here could provide valuable insights into the molecular identity of RGPs in both contexts. Such an analysis might also help elucidate discrepancies related to division modes and lineage restrictions.

Specific comments

>General comment on figure: Many scale bars are missing from pictures (e.g. 1c-1f; 2f-l; Ext data Fig2, …).

>Line 23, page 2. The clonal size unit for projection neurons should be refereed as to the one measure in mouse.

>Line 31, page 2. Please consider removing “even”.

>Line 1, page 3. Please consider removing “to some extent”.

>Line 4, page 3. The concept of self-organization in the introduction is a bit vague, can you elaborate a little for the broad readership of the journal.

>Line 7, page 3. A brief summary/interpretation of the data presented at the end of the introduction would provide valuable context, helping readers appreciate the relevance and impact of this work.

>Line 15, page 3. I suggest to change “genetically-defined dividing progenitor cells” by “genetically-defined progenitor cells undergoing mitosis” (to exclude meiosis)

>Figures 2K and 2L : the SD bars are missing on histograms.

>Line 26, page 7. I suggest to remove “impressively”.

>Figure S8: replace B1 by B2 when appropriate on the histogram legend.

Version 1:

Reviewer comments:

Referee #1

(Remarks to the Author)
The authors have responded thoughtfully and substantially to my concerns. The addition of in vivo developmental scRNA-seq, MADM-CloneSeq, expanded clone quantification, glial analysis, and improved assessment of cell death significantly strengthens the manuscript. In particular, the MADM-CloneSeq data directly address a major concern about clone identity in the absence of cortical lamination. I do not think further major experiments are required, provided the revised figures support the claims described in the rebuttal.

I have a few remaining points that could be handled through textual clarification. First, the Emx1-CreER validation should be framed as supporting comparable Emx1-lineage labeling rather than proving strict RGP specificity unless direct early post-induction co-labeling data are shown. Second, clone architecture categories in organoids should be described as operational MADM-defined clone classes, since spatial and laminar landmarks remain incomplete. Third, the trajectory analysis should be interpreted more cautiously: the absence of a separate transcriptional RGP trajectory does not exclude subtle progenitor heterogeneity or niche-dependent competence states. Finally, claims that cell death is not involved and that gliogenesis proceeds “faithfully” should be softened to reflect the limits of the assays.

Overall, I find the revision strong and the remaining issues largely matters of interpretation and precision.

Referee #2

(Remarks to the Author)
The revised manuscript has been substantially strengthened and the major concerns raised in the initial review have been addressed with extensive additional experiments, analyses, and clarification throughout the text. In particular, the addition of matched in vivo scRNA-seq datasets, improved lineage-tracing analyses, expanded validation of ESC lines, additional MADM-CloneSeq experiments, and improved scRNA-seq analyses significantly enhance the rigor and interpretability of the study.
The manuscript now provides a compelling comparative framework between in vivo corticogenesis and cortical organoid development, and the central conclusion - namely that organoids can recapitulate major cell states and broad developmental ordering while diverging in lineage progression dynamics and lineage restriction behavior - is generally well supported by the presented data. It is also appreciated that several interpretations were refined in response to reviewer comments, particularly regarding neuronal subtype annotations and trajectory analyses.

Only a few remaining points should be addressed during final revision:
1. While the additional in vivo matched scRNA-seq dataset and label-transfer analyses substantially strengthen the developmental comparison between organoids and embryonic cortex, the proposed temporal alignment between organoid differentiation stages and in vivo development remains only partially supported. The conclusion that D8, D13, D20 and D25 correspond closely to E10, E13, E16 and P1 is currently based largely on cell-type abundance, integrated visualization, and marker overlap analyses, which demonstrate broad correspondence in major cell states and developmental ordering but do not establish transcriptome-wide equivalence across matched developmental stages.
2. This temporal alignment is central to interpretation of the clonal analyses. The in vivo MADM framework used for comparison relies on embryonic induction (E10-E13) followed by postnatal analysis (P7-P21/P30), whereas the organoid paradigm uses induction between D8-D15 with collection at D20. Although the revised analyses support approximate correspondence between organoid and embryonic developmental stages up to approximately P1-equivalent states, it remains unclear whether the overall developmental pace and maturation state of the in vitro paradigm are directly comparable to the substantially later postnatal in vivo clonal analyses used as a reference framework. A more quantitative

transcriptome-wide comparison or a reference mapping approach between organoid and in vivo time points (for example a pseudobulk correlation analyses across shared genes, ideally by developmental stage, or transferred labels) would substantially strengthen these conclusions. Otherwise, and it would be sufficient to this reviewer, that claims of “nearly congruent” developmental trajectories and close resemblance to in vivo corticogenesis should be moderated accordingly.
3. The clonal analyses clearly demonstrate that multiple progenitor division modes coexist across organoid differentiation stages and that lineage progression is less temporally constrained than in vivo. However, the data still show gradual temporal shifts in clone size and clone-type abundance across differentiation, suggesting that developmental progression is not fully equipotent or temporally invariant. Since pairwise comparisons between consecutive time points may underestimate gradual developmental trends, statistical approaches that directly test global temporal changes across differentiation stages (for example regression- or trend-based analyses) should be considered. The results of these analyses will confirm wheter terms such as “equipotent cell division capacity” or “loss of temporal stereotypy” are directly supported by the data Alternatively, the corresponding interpretations should be moderated accordingly.

Overall, the revised manuscript represents a significant contribution to the field of cortical organoid biology and developmental neurobiology, and publication is supported after these minor clarifications of the remaining points above.

Referee #3

(Remarks to the Author)
I co-reviewed this manuscript with one of the reviewers who provided the listed reports.

Referee #4

(Remarks to the Author)
The authors have undertaken an impressive and comprehensive body of work, combining both wet-lab and in silico approaches, which effectively addresses all of my major concerns. The revisions have substantially strengthened the manuscript, which now reads clearly and presents a scientifically compelling study. I have only a few remaining minor suggestions.

Title: Consider including the term “brain” or “cerebral” (e.g., “…self-organizing cerebral organoid…”).
Page 3, line 2: Replace “mayor” with “major.”
Page 6, line 8: It would be helpful to clarify—based on cellular and molecular evidence—that D10 corresponds approximately to E10 for comparative purposes.
Page 7: I suggest removing the word “powerful” from the last paragraph.
Figure 2: I recommend removing the “ns” annotations above histograms h–j, as none of the comparisons are non-significant; this can instead be clearly stated in the figure legend.

Open Access This Peer Review File is licensed under a Creative Commons Attribution 4.0 International License, which permits use, sharing, adaptation, distribution and reproduction in any medium or format, as long as you give appropriate credit to the original author(s) and the source, provide a link to the Creative Commons license, and indicate if changes were

made.
In cases where reviewers are anonymous, credit should be given to 'Anonymous Referee' and the source.
The images or other third party material in this Peer Review File are included in the article's Creative Commons license, unless indicated otherwise in a credit line to the material. If material is not included in the article's Creative Commons license and your intended use is not permitted by statutory regulation or exceeds the permitted use, you will need to obtain permission directly from the copyright holder.
To view a copy of this license, visit https://creativecommons.org/licenses/by/4.0/

Response to Reviewers – Stouffer et al., Nature

General statement:

We sincerely thank all reviewers for their constructive and encouraging critiques and thoughtful suggestions to improve our study. We are pleased that all reviewers in principle compliment the high technical quality of our work, agree in general with the conclusions, and the value of conceptual advance. For the revised manuscript, we have added a substantial amount of new original data, addressing all reviewers' points, that we believe significantly advances the study. We have accordingly revised the text (additions and major revisions were highlighted in blue color), made every effort to streamline the manuscript, and incorporate all the valuable suggestions by the reviewers. Below, we first summarize the major new experiments that we have added to the manuscript. We then provide a point-by-point response to the specific critiques by the reviewers.

Summary of new major experiments:

We pursued a series of new scRNA-seq experiments to generate a mouse embryo *in vivo* data set at E10, E13, E16 and P1. These new data enabled rigorous comparison of *in vivo* development and *in vitro* differentiation in organoid in various contexts that are relevant for the main findings and claims in the manuscript. We also extended bioinformatic analysis accordingly. Main data items and analysis related to the comparison of *in vivo* and organoid-based scRNAseq data is presented in new Fig. 1, new Ext. Data Fig. 2-7, 24, 25.

We have added new experiments with the goal to systematically validate the organoid system including pluripotency marker expression and chromosome analysis, including systematic karyotyping of newly generated mESC lines. Data is presented in new Ext. Data Fig. 1, 9, 12.

We carried out morphometric analysis of organoids containing MADM clones using a newly established protocol for organoid clearing and 3D reconstruction. Data is presented in new Ext. Data Fig. 18, 19.

We analyzed glial content in MADM clones in organoids. Data is presented in new Ext. Data Fig. 20.

We added more clones for more in-depth analysis and systematic quantification including statistical analysis of significance across all different lines and batches where appropriate. Data is presented in new Ext. Data Fig. 16, 17, 21. All raw data and statistical analysis is also comprehensively compiled in the Supplementary Tables 1-3.

We implemented a new MADM-CloneSeq protocol which enabled the tracing of RGP-derived lineages *in situ* in combination with single cell transcriptomic analysis based on SMART-Seq v3 technology at single cell level in clonally-related cells. Data is presented in new Fig. 4, new Ext. Data Fig. 22, 23.

Collectively, we believe that the above addition of new experimental data, in combination with more in-depth analysis and statistical assessment, substantially strengthens the manuscript, which should now provide much more depth to support the overall conclusions and conceptual advance. Below we address the more specific items of the reviewers and provide point-by-point responses and refer to respective new data, new and extended analysis, in the Figures:

Point-by-point response (the original reviews are copied in blue)**:**

Referee #1 (Remarks to the Author):

Summary: Stouffer et al. present an extensive clonal analysis of radial glial progenitor (RGP) cell behavior in mouse cortical organoids using Mosaic Analysis with Double Markers (MADM). The authors establish a MADM system in embryonic stem cells and generate cortical organoids to track RGP lineage progression over time. Their data suggest that, unlike the in vivo environment where RGPs undergo a stereotyped and temporally ordered progression from proliferative to neurogenic to gliogenic fates, RGPs in organoids show greater equipotency, reduced cell-type diversity, and increased lineage restriction. The authors conclude that self-organization alone is insufficient to recapitulate the in vivo lineage behavior of RGPs.

Major Comments:

1. Lineage Marker Specificity:
The use of the Emx1-CreER driver raises concerns. While it is intended to mark RGPs, Emx1 is also expressed in intermediate progenitors (IPs) and neurons, both newborn and mature. This could confound lineage tracing results and lead to erroneous interpretations of clone origin and architecture. The authors should explicitly validate the specificity of Emx1 expression in their ESC-derived system.
The reviewer indicates an important issue. To address this point we pursued several analyses. First, we projected reference data [1] in pseudotime onto our organoid scRNA-seq data. Such analysis allowed comparison of *Emx1* expression in reference and organoid during pseudotime. As indicated in the reviewer figure below, we binned pseudotime values into ten bins and calculated mean/standard deviation of normalized *Emx1* expression for all cells in each bin. The plot below shows the mean as points and connected lines with the standard deviation as shaded areas. Overall, the pattern of *Emx1* expression in pseudotime was comparable in our organoid system when compared to *in vivo* reference data.

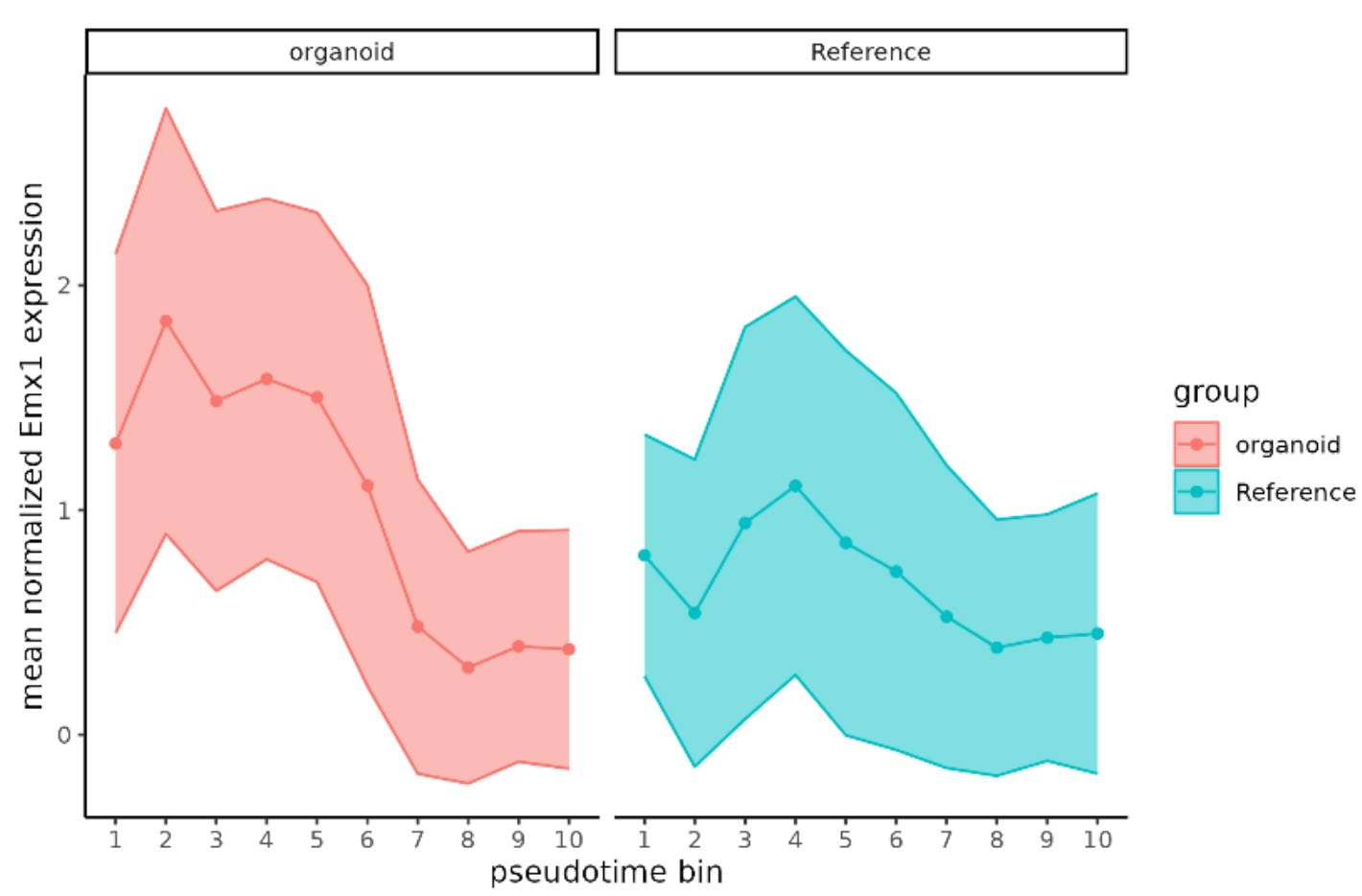

To corroborate the above analysis at higher resolution, we capitalized upon our newly prepared *in vivo* scRNA-seq data set across several developmental time points, allowing rigorous comparison of our organoid system to the actual *in vivo* condition (new Figure 1, new Ext. Data Fig. 2-7). We specifically compared *Emx1* expression in organoid to the newly generated *in vivo* data set. The analysis revealed that *Emx1* expression in all major cell types, including RGPs, intermediate

progenitors (IPs) and neurons in both *in vivo* and *in vitro* models showed highly similar expression patterns as indicated in new Extended Data Figure 7c. From the above analysis we conclude that *Emx1* expression and thus *Emx1*-CreER driver occurs in highly similar pattern in organoid when compared to the *in vivo* model, allowing direct comparison of lineage tracing in organoid to *in vivo* condition.

2. Clone Architecture Interpretation in Organoids:
The classification of clone types (proliferative, asymmetric neurogenic, small neurogenic) relies on spatial organization and ratios of red/green cells, criteria validated in vivo where spatial layering allows neuron identification. In organoids, such laminar organization is often absent. Without reliable positional cues, defining clone types based solely on cell number ratios could be misleading.
The reviewer raised a valid point. While it is true that *in vivo*, previous MADM-based analysis benefitted from the spatial layering to identify postmitotic cortical cell classes, the determination of clone architecture in terms of retrospective interpretation of RGP cell division mode (and thus clone architecture) does not necessarily require the knowledge of nascent postmitotic cell-type identity per-se. Thus, the classification of clones into distinct architectures (as defined by the numbers of cells within the two red/green subclones) based on cell numbers is generally accurate in both organoid and *in vivo* conditions.
We agree however with the reviewer that knowledge of cell-type identity within MADM clones provides much more in-depth information. To this end, we have indeed utilized specific marker expression to identify different classes of neurons (Fig. 3, new Ext. Data Fig. 21), and now in the revision also glia (new Ext. Data Fig. 20, see also below Point 5.). In our revision, we have also performed new MADM-CloneSeq experiments (new Fig. 4, Ext. Data Fig. 22-23) for asymmetric neurogenic and small neurogenic clones that unequivocally identify cell-type identity in MADM clones, even in the absence of specific landmarks such as cortical layers.

The authors make an assumption that red/green labelled cells in organoids represent neurons, unlike the cortical plate context in vivo. This assumption should be addressed explicitly and experiments performed to prove if this is actually valid.
The reviewer is correct, and to address this point we have now extended our analysis and performed several new experiments to obtain in-depth information about cell-type identity in red/green labelled MADM clones. By exploiting the scRNA-seq data set we show that in organoids *Emx1*-expressing RGPs indeed give rise to all expected cell types - excitatory neurons, astrocytes (Astro), oligodendrocytes (Oligo), and olfactory bulb neuroblasts (OBNBs) (new Fig. 1i). Since the reviewer stated also in the above point that tissue organization (and to some extent cell morphology) is not maintained strictly *in vitro*, we expected that a fraction of MADM-labeled cells would represent other cell types than excitatory neurons although the histological and new scRNA-seq transcriptional analysis showed that many of the cells at D20 were indeed neurons, in line with the *in vivo* data (Fig. 1i, Ext. Data Fig. 10l, Ext. Data Fig. 4e). However, in our MADM-CloneSeq analysis we assessed transcriptional signatures for all cell types (not just neurons) in the *Emx1* lineage (normalized marker score, NMS). We found that indeed ~22% (64 cells) of all patched cells (279 cells) showed a higher NMS score for non-excitatory neuron cell types. Out of these, 31 cells displayed a low NMS score, indicating low quality cells. Still, the remaining 33 cells with a good NMS score included aNSC (12 cells), IPs (9 cells), OBNB (6 cells), Oligos (4 cells) and Astro (2 cells). Thus, 87% of high-quality patched cells in the MADM-CloneSeq data set were excitatory neurons, and 13% of cells were cells other than excitatory neurons (as indicated above). We also specifically assessed the content of glial cells within MADM clones and found that glial content in MADM clones in organoids closely resembled the content found previously *in vivo* (Ext. Data Fig. 20, see also Point 5 below).

3. Lineage Restriction and Trajectory Analysis:
The observation of increased lineage restriction in organoids (~32% of clones biased toward either SCPN or CPN fates) contrasts with in vivo (~3%). However, the conclusion that this represents a fundamental divergence requires caution. The observed restriction may be due to technical artifacts such as leaky Cre expression, stochastic behavior in vitro, or even metabolic/culture-related stresses.
The reviewer is correct and we pursued additional experiments and analyses to address the issues. Based on the MADM principle (Ext. Data Fig. 8), all red/green labeled cells in MADM clones derived from a dividing progenitor cell and putative leaky expression in postmitotic cells would result in yellow cells (that were rarely observed and excluded since they were not derived from dividing RGP). Another source of leaky Cre expression could potentially derive from RGPs with an identity different than forebrain. To explicitly address this possibility, we compared *Gfp* expression and predicted tissue origin at each differentiation time point (new Ext. Data Fig 3 and new Ext. Data Fig. 6). These analyses clearly showed that *Gfp* expression and thus *Emx1/CreER* expression was tightly regulated and linked to forebrain tissue as reported *in vivo*.
Stochastic mechanisms in RGPs, generating certain fate biased postmitotic cell-types could indeed be a possibility. Data from our scRNAseq experiments suggest however that RGPs form a relatively uniform pool in organoid and based on our data we did not observe transcriptional signatures that would distinguish RGPs that generate clones with restricted cell-type output from RGPs with multipotent neuron output. Yet, the restricted fate acquisition could still involve stochastic mechanisms and to take this possibility into account we now added a statement in the discussion.
We agree that metabolic and/or culture-related stress is yet another possibility and direct comparison of transcriptomes in RGPs *in vivo* and *in vitro* provided some potential hints (new Ext. Data Fig. 24). We accordingly added a statement in the discussion.

The trajectory analysis shows a single, unitary transcriptional path connecting RGPs to both SCPN and CPN types. This appears inconsistent with the clonal restriction data and should be reconciled. Are the authors possibly over-interpreting a relatively smooth transcriptional trajectory as uniform potency? The trajectory analysis could benefit from clearer explanation and robustness checks. Are there multiple trajectories or forks within the iN cluster? How robust are these to clustering and dimensionality reduction parameters?
We agree with the reviewer and pursued additional experiments and analysis to address this point. In principle, the existence of clones with restricted neuronal output could emerge from a specific separate pool of RGPs, and there would be two putative possible scenarios how corresponding trajectories could manifest in the analysis. 1) An RGP population that shows up as a distinct, organoid specific cluster on UMAP/clustering analysis that then forms a distinct, organoid specific trajectory. To test such prediction, we integrated our new embryonic *in vivo* scRNA-seq data set with the organoid data set, and assessed the overlap of cell clusters indicating different cell types. New Fig. 1j depicts the complete overlap of embryo and organoid RGP populations, strongly arguing against an organoid specific RGP population of considerable size. Furthermore, the RGP population appeared rather homogenous, as reported before [1]. 2) Two or more trajectories would emerge from a relatively homogenous pool of RGPs and end in distinct neuron clusters, one of which would be lineage restricted (i.e. composed of only upper layer or only lower layer neurons). In the original submitted version of the manuscript we used Monocle 3 to identify developmental trajectories. Such analysis is prone to identify forks and sporadic trajectories with increasing cell numbers. To circumvent this caveat and to also test for robustness of the analysis, we now present a new analysis based on slingshot [2]. In the new Fig. 4j-4l we show that there is one single trajectory originating from RGPs and leading, via IPs, to neurons. Our single trajectory

findings were robust to at least three different clustering and dimensionality reduction (UMAP) parameter sets (new Fig. 4j-l and new Ext. Data Fig. 25).

4. Cell Death Considerations:
The manuscript addresses whether clone shrinkage might result from cell death. Caspase-3 staining indicates <6% cell death, comparable to in vivo. This is a valuable control, but the interpretation assumes this rate is uniformly distributed. A deeper analysis (e.g., spatial localization or lineage-stage dependence of apoptosis) would strengthen the conclusion.
The reviewer raises an important issue and we pursued new analysis to address this point. We extended the analysis and quantification of global, whole organoid cell death (using Caspase-3 stainings) over different batches of organoids. We could show that across all three batches cell death was comparably low (new Ext. Data Fig. 11g-11i). Next, we analyzed cell death in the core versus periphery and found that cell death was largely restricted to the core of the organoids. We further noticed that at both D13 and D16 the vast majority of MADM-labeled cells did not colocalize with Caspase-3 (new Ext. Data Fig. 11r-11s). We also extended previous analysis related to 'clone shrinkage' and could show that there is no clone shrinkage between D15 and D20 (new Ext. Data Fig. 17). New analysis using heatmaps demonstrates that clone type distribution was similar for clones with induction time points D8, D10, and D11 with analysis at D15 and D20, respectively (new Ext. Data Fig. 17k-17m). Taken together, our new analysis corroborates the finding that clone size and composition was not majorly influenced by clone shrinkage due to cell death.

5. Gliogenesis:
The authors mention that gliogenesis occurs after neurogenesis, mirroring in vivo behavior. However, this point is underdeveloped. How does this temporally ordered fate switch occur in the absence of stereotyped neurogenesis? Further discussion is warranted.
We agree with the reviewer and pursued additional experiments and analyses to address this point. In our extended analysis at D25 we observed glial cell types in MADM clones, which predominantly emerged after D20 (new Ext. Data Fig. 20). Glial cells could be identified by morphology and/or by using staining against GFAP. Furthermore, we used a transcriptomic approach to show that astrocytes and oligodendrocytes appeared and peaked after neurogenesis. This finding is qualitatively similar to embryonic development and highly reproducible (new Fig. 1i-1j). From our observations in both histological and transcriptomic experiments we conclude that despite the absence of stereotyped neurogenesis, RGPs can faithfully proceed with the neurogenic to gliogenic fate switch. We accordingly added the new data and a statement in the main text of the manuscript.

6. Role of Extrinsic Cues:
A central claim is that non-cell-autonomous, niche-derived cues are necessary for faithful RGP lineage progression. This is plausible and aligns with prior findings (e.g., Nowakowski et al., Bhaduri et al.). However, the paper would benefit from a more detailed discussion of what these cues might be: cell-cell signaling, mechanical forces, metabolic environment? Specific hypotheses or proposed future directions could enrich the discussion.
We followed the reviewer's suggestion and extended the discussion accordingly. In brief, we now elaborate in more detail on the putative nature of the non-cell-autonomous, niche-derived cues, such as extracellular matrix, e.g. [3], extracellular vesicles [4,5], mechanical support cells such as the radial glial fiber grid [6,7], immature nascent projection neurons, or the absence of interneurons,

blood vessels, microglia, choroid plexus/CSF or missing axon targets [[8] and references therein] or certain metabolic microenvironments [9].
We also include a brief discussion about our own new analysis where we compared RGPs from *in vivo* and in *vitro* paradigms at the transcriptional level (new Ext. Data Fig. 24). In this analysis we found that predictions of biological function of DEGs using GO indeed suggest metabolic changes, changes in cell-cell interaction (adhesion), and response to external signaling cues. We assume that these data also could inspire future hypotheses and directions.

7. Title Tone:
The current title is arguably provocative. While the data do show limitations of self-organization, the phrase "self-organization is insufficient" implies a broader generalization that might not apply across all contexts. A more tempered title might reflect the nuance of the findings.
We agree with the reviewer and have adapted the title accordingly. The new title *'Temporal Uncoupling of Radial Glial Progenitor Cell Lineage Progression in Self-organizing Organoid System'* should now better reflect the nuances of our findings.

Minor Comments:

• Typo noted on page 12: "13 CTIP2" should likely read "2 CTIP2+ cells."
We have verified the number of $CTIP2^+$ cells.

• Figure 4b is confusing and not consistent with 4a. The authors should clarify the figure legend and graphical elements.
We thank the reviewer for indicating this important issue. We have now revised the graphics and schematics in an effort to improve clarity and summary of our findings.

• The statistical approach to clone size comparisons (pairwise testing) may obscure meaningful trends; trendline overlays or regression models might be more appropriate.
We agree with the reviewer and have now included bar graph charts with (linear and polynomial) trendlines in new Ext. Data Fig. 16d-16f.

• Authors should cite Nowakowski (human RG lineage studies) and Bhaduri (organoid maturation defects) to situate their findings in broader literature.
We have now included the Nowakowski and Bhaduri works accordingly.

This study addresses an important question in stem cell and developmental neurobiology. The experimental design is thoughtful and leverages the power of MADM for lineage tracing. The findings are intriguing, suggesting that organoids, while powerful, may not fully recapitulate in vivo lineage dynamics. However, interpretation of the data, particularly regarding clone type assignment and transcriptional trajectories, requires caution. Strengthening the discussion of technical limitations, validating key assumptions, and integrating relevant prior work would significantly enhance the manuscript.

We thank the reviewer for the encouraging assessment and for raising the above critical issues that we have now addressed with new experiments and further in-depth analysis as elaborated in the above responses to the specific points raised.

Referee #2 (Remarks to the Author):

The study from Stouffer and colleagues presents an innovative application of the MADM (Mosaic Analysis with Double Markers) system in mouse embryonic stem cell-derived cortical organoids to analyze radial glial progenitor (RGP) lineage progression. The authors directly compare in vitro lineage progression in organoids to the in vivo developmental process, providing a valuable framework to understand the advantages and limitations of using organoids as models for neurodevelopmental studies. The key findings identified by the authors indicate that, unlike the in vivo condition where RGPs exhibit strict temporal lineage progression, RGPs in cortical organoids display a high level of equipotency with increased lineage restriction. This results in a diminished diversity of cortical projection neuron types. Furthermore, despite a seemingly uniform transcriptional profile and unitary lineage trajectory, RGP behavior in organoids differs significantly from the in vivo condition, confirming that non-cell-autonomous cues absent in self-organizing systems play an essential role in normal RGP lineage progression and cortical cell-type diversity.

It is to be noted that this is the first direct comparative analysis of lineage progression in mouse organoids versus in vivo cortical development, which provides crucial insights into how organoid models may recapitulate or diverge from in vivo processes. The study builds upon prior work using MADM for lineage tracing but extends it into the domain of organoid research. This comparison highlights fundamental differences in developmental constraints and plasticity between these systems, contributing substantially to our understanding of the fidelity of organoid models as tools for studying cortical development and disease modeling.
The findings are undoubetly significant for the field, as organoids are extensively used to study neurodevelopment in other species too. Understanding the limitations and advantages of this system may be critical for accurate data interpretation.

The experimental design is well-structured, and the application of MADM in cortical organoids is powerful. An important strength is the use of early passage stocks (P8-15) for organoid differentiation, which is crucial given that prolonged passaging can affect differentiation potential. However, no quality control (QC) analysis of the generated ESC lines is included. This is a critical omission, as evaluating pluripotency and DNA integrity is essential to ensure that newly derived lines maintain their differentiation potential. Including such data would significantly strengthen the study. In addition, it is important to define how robust and reproducible is the used protocol to generate organoids. The MADM lineage tracing approach is a well-established technique, and the authors - expert pioneers of this method - have applied it effectively. The methodology is well described, and the decision to use three independent ESC lines with three differentiations (batches) ensures reproducibility and reliability of results. However, a clearer description of batch variability and statistical approaches used to account for potential batch effects would be beneficial.

We thank the reviewer for the in-depth assessment of our study. We appreciate the encouraging and thoughtful suggestions. In our point-to-point responses below we will provide all details regarding new experiments and analyses, providing quality control such as evaluating pluripotency and DNA integrity in newly generated mESC lines. We also provide more in-depth analysis and clearer statistical assessment of potential batch effect and batch variability in general.

The single-cell RNA sequencing analysis is also well executed and described in the Methods section. However, visualization approaches such as UMAP split by differentiation day or colored by sample would enhance the interpretability of the data.

We thank the reviewer for raising this important issue. We now provide a much more in-depth analysis and improved visualization of the scRNA-seq data in our revised manuscript. Besides adding new extensive scRNA-seq data sets from embryonic time points *in vivo*, that we compare to the *in vitro* organoid data, we also present UMAPs split by developmental age / differentiation day (new Fig. 1 and Ext Data Figs 2-7). We will further elaborate in detail at respective specific points below.

The statistical tests chosen throughout the study appear appropriate for the data presented. However, there are several areas where the reporting of statistical methodology, sample size evaluation, and data presentation require clarification. The assessment of sample size, particularly the number of independent batches, ESC lines, and organoids analyzed, remains unclear. The figure legends primarily state the number of clones but do not specify whether statistical analyses were performed at the level of clones, batch, or cell line. This is particularly relevant when comparing organoid data to in vivo findings, as batch effects and inter-organoid or line variability may influence the observed trends. A more explicit breakdown of the number of independent differentiations, along with statistical considerations for batch and cell line variations, would strengthen the robustness of the conclusions.

We agree with the reviewer and now provide extensive information regarding the statistical methodology, sample sizes, numbers of independent batches and ESC lines. We have now also performed statistical analysis not only at the level of clones but also at the level of batches and independent cell lines. All information regarding raw data and statistical analysis have been comprehensively compiled in the Supplementary Tables 1-3 and/or respective Figure legends. We will also provide more detailed point-to-point responses below.

The conclusions drawn from the study are generally well-supported by the data presented. The study provides strong evidence that cortical organoid models recapitulate some aspects of in vivo cortical development but diverge in lineage progression and progenitor behavior. The observed increase in lineage restriction and equipotency in organoid-derived RGPs is an important finding, emphasizing the need for caution in interpreting organoid-based neurodevelopmental studies. However, additional clarification regarding variability and statistical treatment would further reinforce the reliability of the conclusions.

We thank the reviewer for the encouraging and constructive assessment of our work. Below we will address each of the raised specific points in detail.

1. The validation of the MADM paradigm in the organoid at the population level is unclear (Extended Data Fig. 2). The data show tdT/GFP cells present in organoids and IF of markers. Does "validation at the population level" mean double staining of the reporter and cell population markers? Please clarify. In addition, quantitative analysis should be included, such as determining the percentage of tdT/GFP-positive cells that co-express specific markers at each time point. Statistical quantification should be provided to support this validation.
We thank the reviewer for raising this important point. We agree that our formulation in the previous version of the manuscript was unclear. Therefore, we now explicitly state that we assessed co-labeling of MADM markers with specific markers for RGPs (PAX6), IPs (TBR2) and cortical projection neurons (CTIP2 and SATB2, respectively). The intention was primarily to characterize the cortical organoids at the population level in MADM;*Emx1*-Cre organoids by using specific markers for forebrain cell types. As indicated below, we provide in the revision now much more rigorous validation of the entire system, including immunohistochemistry for pluripotency markers,

DNA integrity and development/differentiation of the system as assessed by scRNA-seq in comparison to the *in vivo* paradigm.
In any case, for the specific marker expression analysis using antibodies against PAX6, TBR2, SATB2 and CTIP2 we now provide quantitative analysis of the cell type proportions across time points (new Ext. Data Fig. 10) with statistical assessment (Supplemental Table 2) according to the reviewer's specific suggestions.
In addition, we now also provide detailed transcriptomic analysis of cell types *in vitro* in comparison to our newly generated *in vivo* embryo data set (using mTmG reporter). We validated the transcriptomic identity of each cell type (new Fig. 1h) and show that cell types are generated in an appropriate relative order in the organoid system (new Fig. 1i and Ext. Data Fig. 4e).

2. Since new original ESC lines are generated, all necessary controls should be provided. It would be useful to include G-banding karyotyping to assess the chromosomal integrity of the ESC lines. Additionally, conducting immunostaining or qPCR for self-renewal and pluripotency markers (e.g., OCT4, SOX2, and lineage markers in embryoid bodies) would strengthen the characterization of these ESC lines.
We agree with the reviewer and pursued new experiments to address the specific points. To this end, we have performed immunohistochemistry for the pluripotency markers Oct3/4 and NANOG in all the newly generated cell lines (Ext. Data Fig 1, 9, and 12). We found that all cell lines expressed these markers faithfully. Next, we performed chromosome counts from metaphase spreads for the two (Line A7 and Line F27) MADM;*Emx1*-Cre lines (Ext. Data Fig. 9) and found them to be within normal range. We have performed extensive karyotyping (Ext. Data Fig. 1 and 12) for the cell lines that were used in scRNA-seq transcriptomic experiments (mTmG;*Emx1*-Cre; Line A and Line B) and for all the cell lines (MADM;*Emx1*-CreER; Line 2A, Line 3A and Line 5C) that were used to assess MADM-based RGP lineage progression at clonal level.

3. The experimental timeline and developmental alignment between organoids and in vivo models require clarification. Is this experimental timeline comparable to in vivo MADM analysis from Gao et al. 2014 (E10-13 injection, analysis at P7-30) and the in vivo data presented in this work (E10 -> P21, Extended Fig. 4)? Are D8-D9 truly consecutive time points at the same pace as E10-E11 in vivo? How was organoid differentiation aligned with in vivo cortical development? A clear temporal comparison between organoid differentiation and in vivo cortical development, leveraging single-cell profiling of both organoids and publicly available in vivo samples, is recommended. also, can the authors confidently say that the organoid generation is as precise and consistent across organoids/batches/lines as the one in vivo between embryos?
The reviewer raised a very important point and we have pursued new in-depth analysis and extensive new scRNAseq experiments to address the issue in detail. First, we have used publicly available data sets [1,10] and assessed quantitative analysis of cell type abundance during embryonic development in comparison to scRNA-seq data derived from our organoid system. We used four major cell types whose abundance changes identify key steps in cortical development (RGPs, IPs, Neurons and Glia). By using these cell type abundance plots we found that D8, D13, D20 and D25 of organoid *in vitro* development matched well with E10, E13, E16 and P1 *in vivo* time points (Ext. Data Fig. 4e).
To corroborate the above analysis we prepared a new scRNA-seq data set from mTmG;*Emx1*-Cre embryos (matching precisely the genotype of the mTmG;*Emx1*-Cre organoid scRNA-seq data set) at E10, E13, E16, and P1, using 10X FLEX technology. Note: we used exactly the same genotypes and sequencing technology for the new embryo data set in order to allow for direct comparison (without technical bias) to the previously generated organoid data set. By using now all three embryonic data sets [newly generated scRNA-seq data set from this study, and two

reference data sets [1,10]] we show that the abundance of the different cell types is both highly reproducible between organoid batches and very similar to *in vivo* data (new Fig 1i and new Ext. Data Fig. 4e). In conclusion, given the high similarity of cell type abundance trajectory between *in vivo* embryo and *in vitro* organoid we conclude that our experimental timeline and developmental alignment between organoid and *in vivo* is nearly congruent, and that the organoid generation is precise and consistent across organoids/batches and different mESC lines.

4. Two key differences were found between cortical organoids and in vivo tissues.

4.1 The presence of small neurogenic clones is particularly interesting, as it represents a potential difference from in vivo conditions. Could this be due to artificial conditions or missing signaling cues in organoids? Can the authors discuss these distinctions.

The reviewer raised an important aspect and we address the point to some extent experimentally and elaborate in more detail in our discussion. In our extension of the discussion, we mention that metabolic and/or culture-related aspects such as cellular stress could result in distinct lineage outcomes *in vitro* when compared to the *in vivo* paradigm. In a first pass series of experiments, we focused on the transcriptomes of RGPs and compared gene expression of RGPs *in vitro* to the ones of RGPs *in vivo* (new Ext. Data Fig. 24). Interestingly, we found that predictions of biological function of DEGs using GO indeed suggest metabolic changes, changes in cell-cell interaction (adhesion), and response to external signaling cues. In our discussion we further elaborate also more generally on the possible limitations and putative nature of non-cell-autonomous, niche-derived cues, such as extracellular matrix [3], extracellular vesicles [4,5], mechanical support cells such as the radial glial fiber grid [6,7], immature nascent projection neurons, or the absence of interneurons, blood vessels, microglia, choroid plexus/CSF or missing axon targets [[8] and references therein].

4.2 Increased lineage restriction (clones containing mainly Ctip2+ cells or Satb2+ cells) was observed in cortical organoids (30%) compared to in vivo tissues (3%). It would be valuable to investigate small neurogenic clones and the increased lineage restriction further by performing lineage tracing experiments using additional approaches, such as genetic barcoding, to allow analysis at the single-cell transcriptome level. This could help clarify whether these differences are due to intrinsic properties of the organoid system or whether they reflect a developmental delay or differences in culture conditions.

The reviewer indicates a critical point and we thank the reviewer for the suggestion to probe lineage tracing with single cell transcriptome resolution. We have carefully assessed the experimental possibilities using barcoding approaches but the current tools are limited, especially due to the low clone coverage, in combination with our MADM-based approach. Therefore, we capitalized on MADM-CloneSeq, a technology that we have previously implemented in the laboratory [11,12]. By using this approach, we have directly collected MADM-labeled clonally related cells and subjected them to SMART-seq v3 based scRNA-seq. In total, we collected 287 cells from 68 clones (45 organoids). After QC and assigning cells an upper/lower layer cell identity, we could analyze 21 asymmetric neurogenic and 34 small neurogenic clones (195 cells in total) with a coverage of 67% (asymmetric neurogenic clones) and 73% (small neurogenic clones) (new Ext. Data Fig. 23a). Cells associated with asymmetric neurogenic and small neurogenic clones showed no major transcriptional differences, based on the analysis of projecting MADM-CloneSeq cells onto the reference UMAP and nearest neighbor analysis (Fig. 4b-d, Ext. Data Fig. 23b). Next, we assigned each MADM-CloneSeq cell to deep (DL) and upper layer (UL) neuronal identities to unequivocally identify lineage-restricted clones (Extended Data Fig. 22a, Ext. Data Fig. 23c-e). We found lineage restricted clones among both asymmetric and small neurogenic clones with similar abundance as reported for histological analysis (Chi-Square, $p > 0.3$) (Ext. Data Fig.

23d). To test whether lineage restriction observed in organoids is associated with putative developmental delay, we performed pseudotime analysis of asymmetric and small neurogenic clones (Fig. 4e-h). However, we found no significant difference between lineage-restricted and non-restricted (translaminar) small neurogenic clones (Fig. 4h).

5. Batch, cell line, and organoid-specific variations should be considered in the analysis. Throughout the manuscript, it is unclear whether batch, cell line, and organoid-specific variations were accounted for. We strongly suggest performing an additional analysis to assess how these factors influence clone size and other phenotypic traits. This could be done by including information on the number of batches used and conducting batch or line comparisons during statistical analysis. The authors should also include this information in the plots to show variability across lines and batches and clarify how these factors influence outcomes.
We thank the reviewer for the suggestion and we have addressed this point throughout the revised manuscript in several ways and by performing additional analysis. We now include analysis of MADM clone size (i.e. cells/clone) by taking into consideration of individual lines and batches. These data are presented in new Ext. Data Fig. 16a-16c. We also increased the total clone number for the analysis of lineage restriction and taking into account individual batches (new Ext. Data Fig. 21). All data is also comprehensively compiled, including information on the number of batches and cell lines, together with statistical analysis in respective Figure legends and the Supplementary Tables 2-3.

6. The output of the analysis comparing MADM clone architecture at different time points (D15 and D20) should be presented clearly in the figure. Additional details, such as bar graphs or heatmaps, should be included to show the relative proportions of clone types at each induction time point. A summary table or statistical comparison across time points would also help clarify the results.
We followed the reviewer's suggestion and have now replotted the data to compare clone type abundance at the three induction timepoints (D8, D10 and D11) with analysis at D15 and D20, respectively. To this end we have also included a color-coded heatmap to indicate relative proportions and abundance of each clone type at the respective timepoint. We found that the data for D15 and D20 clones was overall remarkably similar when tested for statistical significance. The analysis is now presented in new Ext. Data Fig. 17k-m and statistical information has been listed in Supplementary Table 2.

7. Variability and statistical analysis of CTIP2+ and SATB2+ clones should be provided. The authors should ensure that variability is shown in the analysis of CTIP2+ and SATB2+ clones by including error bars (e.g., standard deviation or SEM) in the figure. Additionally, the statistical tests used to compare the groups should be clearly stated, and appropriate statistical methods (e.g., ANOVA, t-tests) should be employed to test for significant differences. Providing raw data or supplemental information on the statistical analysis would improve transparency.
We have now added error bars and the charts have been updated accordingly in Fig. 3k. We perfomed a 2-way ANOVA and identified a higher abundance of $CTIP2^+$ clones, compared to $SATB2^+$ clones, only at D8 induction time point (Ext. Data Fig. 21; Suppl. Table 2). We also performed a chi-squared test to show that we have a higher abundance of lineage restricted clones at both D8 and D13 induction timepoints in organoids compared to *in vivo* (Fig. 3l). We also provide all raw data and details concerning statistical analysis in Supplementary Table 2.

8. The low percentage of GFP+ cells in D8 single-cell experiments contradicts in vivo Emx1 expression. The authors report a low percentage of GFP+ cells in D8 samples from the single-cell experiment. This contradicts in vivo Emx1 expression. Could this be due to delayed recombination or misalignment between organoid differentiation and in vivo development? Additionally, why do the authors rely on GFP recombination to sort Emx1-positive cells instead of using Emx1 transcript expression directly? To address this, we suggest investigating recombination efficiency at earlier stages (e.g., day 6 or 7) to determine whether delayed recombination could explain the low GFP+ cell numbers. A temporal comparison between in vivo Emx1 expression and organoid differentiation should also be performed to check for alignment in timing.

The reviewer indicates an important point and we pursued additional analysis to address the issue. In the *in vivo* paradigm, *Emx1* expression is restricted to forebrain RGPs during early brain development. As such it might have been possible that the lower percentage of *Gfp*$^+$ cells in early organoids was due to slightly incomplete patterning to forebrain tissue at the earliest stages. To investigate such possibility, we assigned tissue identity to organoid cells by using a reference data set [10]. Such analysis showed that at D8, 1) a sizeable fraction of cells showed signatures of tissues other than forebrain (i.e. hindbrain, midbrain); and 2) only forebrain RGPs expressed *Gfp*. From D13 onward, the majority of cells expressed *Gfp*, indicating complete forebrain patterning in the organoids. Based on these results we conclude that slight incomplete patterning is the cause for a lower amount of *Gfp*$^+$ cells at D8.

To address the issue further we compared *Gfp* expression and predicted tissue origin at each differentiation / developmental time point in organoid and *in vivo*, to rigorously assess alignment in timing. These data are presented in new Ext. Data Fig. 2, 3, 5, and 6. Although *Emx1* is expressed to some extent in more differentiated cell types, expression levels tend to decrease over time (new Ext. Data Fig. 3 and 6). Thus, marking and detecting cell types of the *Emx1* lineage by using *Gfp* might be a more sensitive approach.

We also could not detect any misalignment of organoid differentiation and *in vivo* development (Ext. Data Fig. 7c). Lastly, we projected reference data [1] in pseudotime (new Fig. 4e-f) onto our organoid scRNA-seq data. Such analysis allowed comparison of *Emx1* expression in reference and organoid during pseudotime. As indicated in the reviewer figure below, we binned pseudotime values into ten bins and calculated mean/standard deviation of normalized *Emx1* expression for all cells in each bin. The plot below shows the mean as points and connected lines with the standard deviation as shaded areas. Overall, the pattern of *Emx1* expression in pseudotime was comparable in our organoid system when compared to *in vivo* reference data.

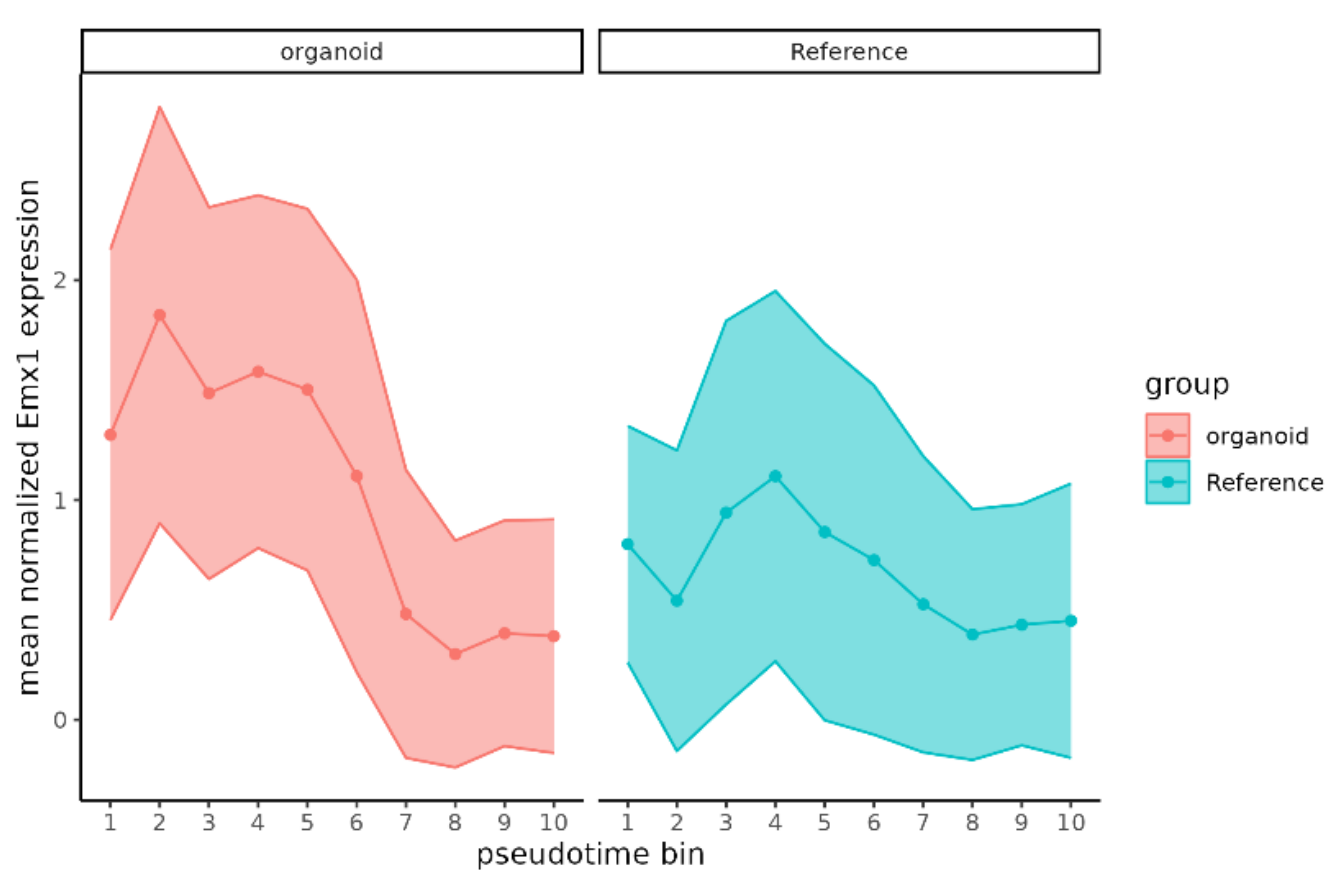

9. Separation between SCPN and CPN clusters in scRNA-seq data is unclear. There is no clear separation between SCPN and CPN clusters. A marker table or annotation dot plot is missing. To improve clarity in the scRNA-seq analysis, we suggest including a marker table with key genes expressed in SCPN and CPN clusters. It may also be worthwhile to reassess the clustering parameters used in the scRNA-seq analysis. Alternative methods, such as adjusting clustering resolution or applying different dimensionality reduction techniques, may help achieve better separation between SCPN and CPN populations.
We agree with the reviewer that in the previous Fig. 3 neurons appeared as a relatively homogenous group of cells. Our primary intention in the previous analysis was the mapping of trajectory originating from RGPs and to assess whether multiple pools of RGPs can be detected in the scRNA-seq data. However, we agree that the presence of upper layer and lower layer neurons in the organoid data is of general interest. We therefore now performed an analysis where we integrated RGPs, IPs and neuronal cells from *in vivo* reference [1], our organoid data as well as the newly generated MADM-Clone-Seq data. In this new analysis (see new Fig. 4b), we show clear separation of UL/DL neurons.

We also followed the reviewer's advice and pursued marker overlap analysis as shown in new Ext. Data Fig. 22b between UL/DL cells in organoid and embryo. Such analysis revealed a large number of genes expressed specifically in UL or DL, respectively; and that UL and DL cells in organoid were transcriptionally similar to UL and DL cell *in vivo* (new Ext. Data Fig. 22a-b). We provide the list of DEGs in UL vs DL in Supplementary Table 2.

10. The inferred trajectory in scRNA-seq analysis appears over-branched. The trajectory inference appears overly complex, which may obscure meaningful developmental relationships. Adjusting trajectory inference parameters or using alternative methods may provide a better representation of the differentiation trajectory. Additionally, pseudotime scoring should be included to further assess differentiation processes.
We agree with the reviewer that the previous trajectory analysis was overly detailed and did not make our point in a concise and intuitive way. We therefore followed the reviewer's advice and used an alternative method to also test for robustness of the analysis. We now present a new analysis based on slingshot [2]. In the new Fig. 4j-4l we show that there is one single trajectory originating from RGPs and leading, via IPs, to neurons. Our single trajectory findings were robust to at least three different clustering and dimensionality reduction (UMAP) parameter sets.

We also took the advice of the reviewer to perform pseudotime scoring. Due to the focus of our study and not to defer from the main message, we did not include this data in the main manuscript but provide the information here. In brief, we identified pseudotime scores for reference cells in reference data set [1]. We next transferred pseudotime scores to our organoid dataset and compared pseudotime scores in each cell type between *in vivo* reference and organoid. Such analysis showed no significant difference in developmental progression between *in vivo* and organoid paradigm. In summary, projecting reference pseudotime analysis (new Fig. 4e-f) on the organoid data set revealed similar developmental progression of major cell types in both *in vivo* and *in vitro* contexts. The reviewer figure below shows the mean and standard deviation of pseudotime of cells in the indicated cell type, separately for organoid and *in vivo* mouse reference [1].

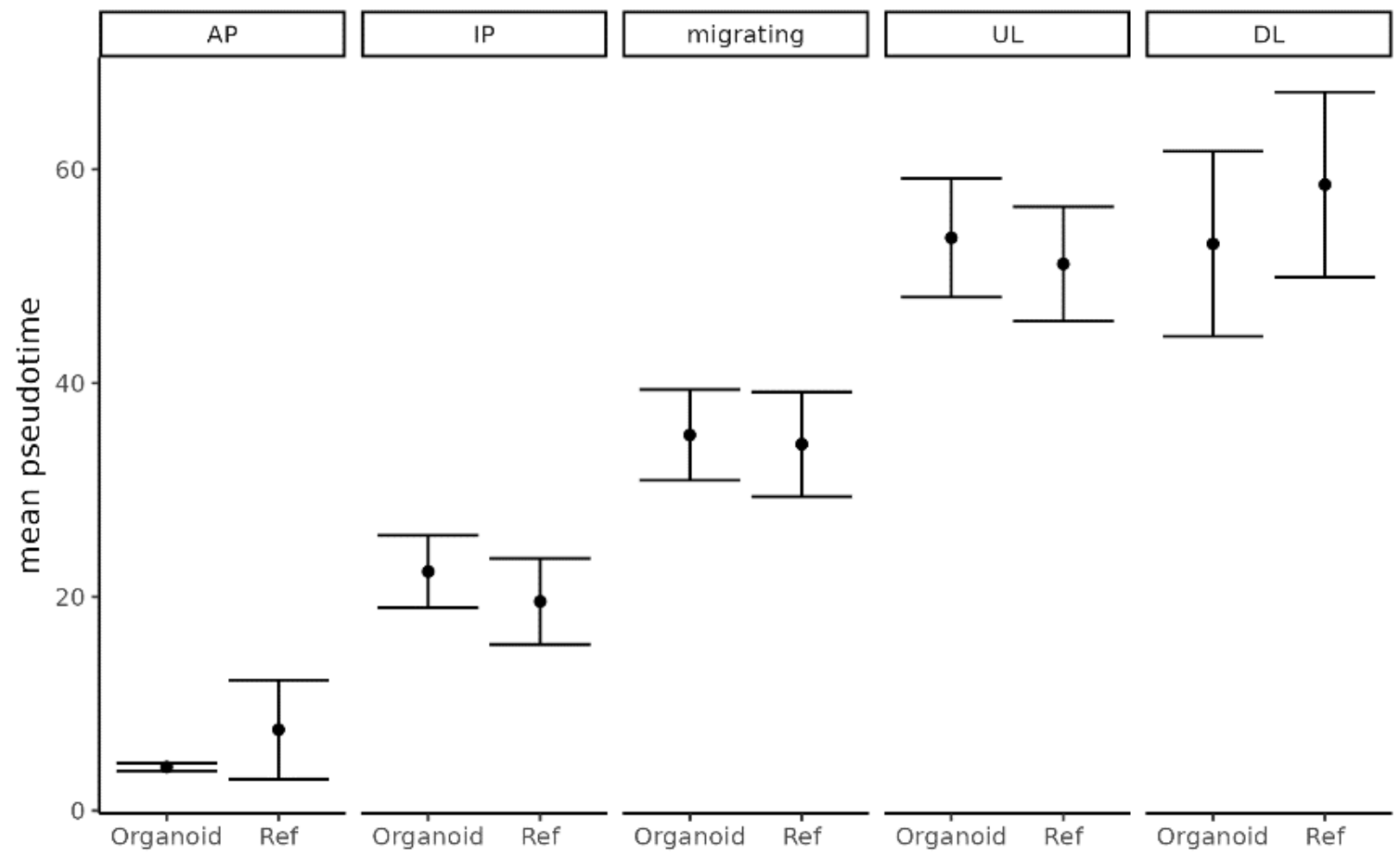
AP
IP
migrating
UL
DL
mean pseudotime
60
40
20
0
Organoid
Ref
Organoid
Ref
Organoid
Ref
Organoid
Ref
Organoid
Ref

Referee #3 (Remarks to the Author):

I co-reviewed this manuscript with one of the reviewers who provided the listed reports.

Referee #4 (Remarks to the Author):

In this study, Stouffer and colleagues explore the progression of radial glial progenitors (RGPs) in vitro using mouse self-organizing cerebral organoids. RGPs, known to drive cortical cell lineage progression, are typically studied in vivo in murine models, where this process is believe to be largely driven by cell-intrinsic factors. However, the surrounding stem cell niche also plays a crucial role in facilitating this lineage progression. Previous studies have shown that RGPs cultured in 2D systems exhibit limited lineage development, suggesting that non-cell-autonomous factors are essential but so far poorly understood. Using mosaic analysis with double-marker-based lineage tracing in organoids, they found that RGPs in these 3D cultures showed division mode defects, restricted lineage progression, and reduced cell-type diversity in projection neuron clones, as compared to in vivo conditions. While organoids maintained the overall genetic program of RGPs, the absence of environmental niche cues in the 3D culture system limited their ability to fully replicate natural lineage progression and neural diversity.
This study underscores the importance of considering the limitations of organoid models when interpreting neural development data. The experimental design is solid, and the manuscript is well-written for a broad audience, but additional analyses and expanded discussion would further strengthen the work. Overall, it serves as a valuable reminder to approach organoid-based findings with caution, especially when exploring the complexities of neural cell lineage progression.

We appreciate the reviewer's rigorous assessment of our work, and the encouraging and constructive suggestions to improve our study. Below we elaborate in detail in the point-to-point response.

Main comments

1. Does neural cell lineage progression vary across presumptive cortical areas in the Emx1 MADM mouse model? If so, could inadequate establishment of arealization in cerebral organoids hinder proper lineage progression within these self-organizing structures?
The reviewer raises an important issue. Previous efforts to study neural stem cell lineage progression *in vivo* in the MADM mouse model have not revealed any differences in distinct presumptive cortical areas. We however note that most analysis has been carried out in the somatosensory cortex but that the sparse data from other cortical areas has not indicated differences in RGP lineage progression. We therefore, with current evidence, cannot make solid predictions if arealization or its absence, *in vivo* and/or in the organoid may be relevant for specific aspects of RGP lineage progression, although we can also not completely exclude this possibility.

2. The final concentration of 4-OHT used to generate a single clone per organoid is reduced by almost half specifically at the point when a significant decrease in observed cell clone size is noted (see Materials and Methods and Figure 1G). What is the scientific rationale for this concentration adjustment, and does it pose a risk of introducing technical bias?
In our MADM clone induction paradigm we aimed at empirical determination of the optimal dose of OHT with the goal to induce ideally one or just very few MADM clones in a single organoid. Through a series of titration, we found that the optimal OHT dose for our MADM clone induction paradigm varied slightly, depending on the differentiation time point. However, the application of a certain dose of OHT does not correlate with the clone size (i.e. number of MADM-labeled cells) but rather with the interchromosomal recombination frequency (new Ext. Data Fig. 8), which is an all or nothing event. In other words, higher OHT concentration increases the rate of recombination events and thus the number of clones but not the number of labeled cells within a MADM clone.

We are therefore confident that adjusting the OHT dose did not introduce a technical bias, similar to our previous (and current) analyses *in vivo* that also required TM adjustment for tuning the recombination frequency and overall number of MADM clones present in a defined region of interest.

3. Figure 1J. The observation of an unexpectedly high proportion of small neurogenic clones in organoids is noteworthy. By examining clones five days earlier, the authors suggest that these small neurogenic clones are not artifacts resulting from "depleted" proliferative clones. They show that activated caspase immunoreactivity remains low and stable throughout the culture period. However, since apoptosis represents only one form of cell death, what about necrosis (and possibly ferroptosis), which is usually known to accumulate, particularly in the central core of organoids?
The reviewer is correct and there are a number of pathways that lead to cell death. However, the detection of dying cells is challenging, including the cells that die via apoptosis since specific markers are only present for a short time and once cells are gone there is no way that cells can be spotted retrospectively. In any case, we used Caspase-3 immunohistochemistry as a proxy to estimate the approximate loss of cells in organoid system. In our revision we have now extended the analysis (also in response to Reviewer 1, point 4) and determined 1) whole organoid cell death (using Caspase-3 staining) over different batches of organoids. We could show that across all three batches cell death was comparably low (new Ext. Data Fig. 11g-11i). Next, we analyzed cell death in the core versus periphery and found that cell death was largely restricted to the core of the organoids. We further noticed that at both D13 and D16 the vast majority of MADM-labeled cells did not colocalize with Caspase-3 (new Ext. Data Fig. 11r-11s).

Taking into account the notion of the reviewer that there are other pathways that lead to cell death/elimination (besides apoptosis) we also extended our previous analysis related to 'clone shrinkage'. The rationale was that cell depletion by any form of cell death should, if significant, lead to distinct proportions and abundance of specific clone types when compared at D15 versus D20. We however could show that there is no clone shrinkage between D15 and D20 (new Ext. Data Fig. 17). New analysis using heatmaps demonstrates that clone type distribution was similar for clones with induction time points D8, D10, and D11 with analysis at D15 and D20, respectively (new Ext. Data Fig. 17k-17m). Taken together, our new analysis corroborates the finding that clone size and composition was not majorly influenced by clone shrinkage/erosion due to cell loss by any form of cell death/loss.

Additionally, is there any correlation between organoid size/rosette enrichment and the ability to generate the three distinct clone architectures?
To address the above reviewers point we pursued additional analysis and experiments. First, we investigated whether rosette morphology (surface area) correlated with any of the three clone types, but we found no correlation (n=30 rosettes) (new Ext. Data Fig. 18). The only difference that we noticed was that small neurogenic clones had a smaller footprint throughout the rosette when compared to asymmetric neurogenic clones, which occupy more space in the rosette (new Ext. Data Fig. 18c) since asymmetric neurogenic clones also show larger clone size.
Next, we generated 3D reconstructions of 20 cleared organoids and tested for clone type enrichment based on organoid morphology. We found no link between overall organoid morphology and clone architecture content (new Ext. Data Fig. 19). In effect, organoids of similar volume tended to have 1-2 different clone types, with the two largest organoids having all three clone architectures (new Ext. Data Fig. 19d). Finally, we found that around 55% of organoids contained more than one clone type (new Ext. Data Fig. 19e).

4. Figure 2. Lineage-restricted clones are frequently relatively small, particularly for CTIP2+ neurons. To what extent could this be influenced by cell death or the relative proportion of radial glial cells (RGCs, or apical progenitors) versus intermediate progenitors (BPs), which are primarily responsible for generating callosal projection neurons (CPNs) in vivo in mice?

The reviewer raised an important point. Based on our analysis, approximately 50% of lineage restricted small clones were CTIP2$^+$ (only) at D8 induction. This finding is likely not majorly influenced by cell death because we found that overall cell death was rather limited (only approximately 6% of MADM$^+$ cells) (new Ext. Data Fig. 11). Furthermore, and as detailed above in Point 3, Caspase-3 staining was enriched in the core rather than the periphery of the organoid (new Ext. Data Fig. 11), and MADM clones were predominantly located in the periphery. As discussed in the point 3 above, we also found no difference in small neurogenic clone (that overall include a large proportion of small CTIP2$^+$ clones) abundance at D15 and D20 analysis timepoints, leading us to conclude that cell death is not a major contributing factor related to the abundance of small neurogenic clones.

For our revision we have generated an entire new scRNA-seq data set from *mTmG* mouse embryos in time course (E10, E13, E16, and P1). These new data enabled rigorous comparison of *in vivo* condition to organoid (where we previously generated scRNA-seq data set for D8, D13, D20 and D25) with regards to emergence and abundance of distinct cell types, including RGPs and IPs (new Fig 1, new Ext. Data Fig. 4e). Importantly, we found that relative abundance of progenitor populations in organoids reflected very closely the progenitor populations in *in vivo* paradigm (new Fig 1i), and we thus conclude that in the absence of significant differences there is no influence regarding the occurrence of small lineage-restricted clones.

5. Figures 3 and S8. The scRNA-seq analysis reveals that some Emx1-derived cell clusters, specifically clusters 8, 10, and to a lesser extent 6, include Dlx1+ interneurons—an observation not observed in similar in vivo analyses in mice. What could explain this discrepancy? Might this indicate an artifact, suggesting additional compromised lineage progression in vitro? This finding is significant and warrants further discussion. Additionally, it has important implications for the interpretation of Figure 3F, where data were extracted from the original dataset to focus on the "neural developmental lineage".

We agree with the reviewer that we did not explicitly introduce the complete lineage relationship of all cell types in the *Emx1* lineage. As such the presence of interneurons in our data set might seem unexpected. During cortical development *Emx1*$^+$ RGPs first produce excitatory projection neurons and, towards birth, switch their potential to give rise to astrocytes and oligodendrocytes (likely via committed intermediate progenitors) and establish the adult stem cell niche that mainly produces olfactory bulb neuroblasts (OBNBs) that migrate to the olfactory bulb to integrate as olfactory bulb interneurons [13]. OBNBs function as interneurons, and express canonical interneuron markers like *Dlx* and *Gad* genes [13-15]. We have now integrated scRNA-seq data from the *Emx1* lineage from organoid data and *in vivo* embryo data (new Fig. 1). In new Fig. 1j we show that OBNBs form a distinct and overlapping cluster of cells in both organoid (D25) and *in vivo* mouse (P1). These OBNBs appear late in development, are closely related to the aNSC cluster and are clearly distant to the excitatory neuron clusters. These findings further corroborate faithful modelling of lineage progression *in vitro*. For in-depth detailed neuronal lineage analysis (new Fig. 4), we did not include OBNBs for simplicity and more straight forward analysis.

6. Figure S8. It would be helpful to provide a clear correspondence between cluster numbers and cell types, perhaps in the form of a table listing key marker genes used for the classification of major cell types. Additionally, what is the in vivo equivalent of aNSCs in the mouse cortex, and

how distinct are these cells from radial glial progenitors (RGPs)? There appears to be no reference to these cells in the main text.
We agree with the reviewer to increase precision in nomenclature of the different cell types. The new Ext. Data Fig. 7b depicts a heatmap of marker genes for each identified cell type. The group of cells named aNSC in the initial version of the manuscript forms a broad cloud on the UMAP and shows marker genes for astrocytes, oligodendrocytes and OBNBs. All these cell types originate from *Emx1*⁺ RGPs *in vivo* [16-18]. Based on the finding that these cells also show markers for active cell cycle, we conclude that this group is a mix of committed intermediate progenitors for astrocytes, oligodendrocytes, as well as adult stem cells, producing OBNBs [16-18]. We have renamed this cell cluster in new Fig. 1g for clarity, but kept aNSC as a short name for other figures. The legend for the new Fig 1 indicates this accordingly.

Regarding the unitary trajectory from progenitors to projection neurons, the developmental trajectory depicted by Monocle3 (Figure 3I) aligns with expectations based on in vivo or in situ analyses of the mouse cortex. However, since this observation relies on a “cell context-extracted” dataset, is the “projection neuron trajectory” still preserved when considering a broader context that includes additional cell types, as shown in Figures S8 (where two connected lineages may be guessed)?
The reviewer raised an important point. Indeed, the developmental trajectory of the *Emx1* lineage is complex, since *Emx1*⁺ RGPs produce many different cell types during development. In the neurogenic phase, RGPs produce most (if not all) excitatory neurons of all cortical layers. As development progresses towards birth, RGPs switch their potential to produce astrocytes, oligodendrocytes (likely via committed intermediate progenitors) and some RGPs progress to adult neural stem cells that produce OBNBs that migrate to the olfactory bulb. Capturing all trajectories at once in a single analysis is technically very challenging at many levels. Our focus was the developmental trajectory from RGPs to neurons, which is well captured in our data. However in order to address the reviewer’s point, to test whether the unitary developmental trajectory found in a focused analysis (only containing RGPs, IPs and Neurons, new Fig. 4j-l) is also detectable in the context of all cell types, we prepared a 3D UMAP of the data shown in Fig. 1g and analyzed the data with slingshot (getLineages(data = [3D UMAP embeddings], clusterLabels = [Seurat_clusters], dist.method = "simple")). We provide this data as animated Supplementary Reviewer Figure (animated gif). Colors indicate the cell types as in Fig. 1g and the neuronal trajectory is shown in black, all other detected trajectories in grey. We focused on the Seurat custers (FindClusters (resolution = 0.8)) that are most prevalent in E13, E16 or P1 embryo data set, since the earliest data sets (D8/E10) formed a separate cluster in this analysis. Note that we used all 116115 cells (organoid and mouse *in vivo*) as shown in Fig. 1g for this analysis.

7. In the discussion: The authors note the importance of comparing the transcriptional profiles of RGPs in vivo and in cerebral organoids. Performing this comparison here could provide valuable insights into the molecular identity of RGPs in both contexts. Such an analysis might also help elucidate discrepancies related to division modes and lineage restrictions.
We followed the advice of the reviewer and prepared an entire new scRNA-seq data set from *in vivo* embryo that we then used for comparison to scRNA-seq data from organoids. The data is presented in new Ext. Data Fig. 24 with a direct and specific comparison of RGPs from D8/E10 and D13/E13. Differential gene expression (DEG) analysis was most pronounced at early, compared to late progenitors, with little overlap between the two time points. Gene ontology (GO) term analysis indicates that metabolism and signaling are amongst the more specific changes. Thus, the new data set indeed provides valuable insights into the molecular differences of RGPs in organoid when compared to *in vivo*. While the data does not provide explicit insights regarding

the discrepancies related to RGPs division mode and lineage restriction in organoid when compared to *in vivo*, the nature of the predicted DEGs and associated GO terms (e.g. metabolic changes, cell-cell interaction/adhesion, response to external signaling cues) seem to emphasize the importance of an intact stem cell niche *in vivo*. Hence, we utilize this new data in the context of our discussion related to the nature of non-cell-autonomous cues that might result in the differences in RGP lineage progression in organoid when compared to *in vivo* paradigm.

Specific comments

General comment on figure: Many scale bars are missing from pictures (e.g. 1c-1f; 2f-I; Ext data Fig2, …).
We have now inserted scale bars in all figures and defined them accordingly in the respective figure legends.

Line 23, page 2. The clonal size unit for projection neurons should be refereed as to the one measure in mouse.
We revised the text accordingly and now explicitly refer to mouse when we mention the clonal unit size.

Line 31, page 2. Please consider removing “even”.
We removed ‘even’ according to the reviewer’s suggestion.

Line 1, page 3. Please consider removing “to some extent”.
We followed the reviewer’s suggestion and removed ‘to some extent’

Line 4, page 3. The concept of self-organization in the introduction is a bit vague, can you elaborate a little for the broad readership of the journal.
We followed the reviewer’s suggestion and provide more context in the introduction related to the concept of self-organization in our revised version of the manuscript.

Line 7, page 3. A brief summary/interpretation of the data presented at the end of the introduction would provide valuable context, helping readers appreciate the relevance and impact of this work.
We thank the reviewer for their suggestion and accordingly added a brief summary/interpretation at the end of the introduction.

Line 15, page 3. I suggest to change “genetically-defined dividing progenitor cells” by “genetically-defined progenitor cells undergoing mitosis” (to exclude meiosis).
We have followed the reviewer’s suggestion and now state ‘genetically-defined progenitor cells undergoing mitosis’.

Figures 2K and 2L: the SD bars are missing on histograms.
We added the standard error of proportion in this figure (now new Fig. 3) and provide the raw data in the Supplementary Table 2, 3. In new Ext. Data Fig. 21 we now also provide all data separated by batch as requested by reviewer 2.

Line 26, page 7. I suggest to remove “impressively”.
We followed the reviewer’s suggestion and removed ‘impressively’.

Figure S8: replace B1 by B2 when appropriate on the histogram legend.

In the course of the revision, we have substantially extended the scRNA-seq analysis and some of the previous histograms has been replaced with new data figures (new Fig. 1, new Ext. Data Fig 2-4 and new Ext. Data Fig. 7)

References:

1 Di Bella, D. J. *et al.* Molecular logic of cellular diversification in the mouse cerebral cortex. *Nature* **595**, 554-559 (2021). https://doi.org:10.1038/s41586-021-03670-5

2 Street, K. *et al.* Slingshot: cell lineage and pseudotime inference for single-cell transcriptomics. *BMC Genomics* **19**, 477 (2018). https://doi.org:10.1186/s12864-018-4772-0

3 Long, K. R. & Huttner, W. B. The Role of the Extracellular Matrix in Neural Progenitor Cell Proliferation and Cortical Folding During Human Neocortex Development. *Frontiers in cellular neuroscience* **15**, 804649 (2021). https://doi.org:10.3389/fncel.2021.804649

4 Forero, A. *et al.* Extracellular vesicle-mediated trafficking of molecular cues during human brain development. *Cell reports* **43**, 114755 (2024). https://doi.org:10.1016/j.celrep.2024.114755

5 Pipicelli, F. *et al.* Non-cell-autonomous regulation of interneuron specification mediated by extracellular vesicles. *Science advances* **9**, eadd8164 (2023). https://doi.org:10.1126/sciadv.add8164

6 Nowakowski, T. J. *et al.* Spatiotemporal gene expression trajectories reveal developmental hierarchies of the human cortex. *Science* **358**, 1318-1323 (2017). https://doi.org:10.1126/science.aap8809

7 Nowakowski, T. J., Pollen, A. A., Sandoval-Espinosa, C. & Kriegstein, A. R. Transformation of the Radial Glia Scaffold Demarcates Two Stages of Human Cerebral Cortex Development. *Neuron* **91**, 1219-1227 (2016). https://doi.org:10.1016/j.neuron.2016.09.005

8 Villalba, A., Amberg, N. & Hippenmeyer, S. in *Neocortical Neurogenesis in Development and Evolution* 169-191 (2023).

9 Bhaduri, A. *et al.* Cell stress in cortical organoids impairs molecular subtype specification. *Nature* **578**, 142-148 (2020). https://doi.org:10.1038/s41586-020-1962-0

10 La Manno, G. *et al.* Molecular architecture of the developing mouse brain. *Nature* **596**, 92-96 (2021). https://doi.org:10.1038/s41586-021-03775-x

11 Cheung, G., Pauler, F. M., Koppensteiner, P. & Hippenmeyer, S. Protocol for mapping cell lineage and cell-type identity of clonally-related cells in situ using MADM-CloneSeq. *STAR protocols* **5**, 103168 (2024). https://doi.org:10.1016/j.xpro.2024.103168

12 Cheung, G. *et al.* Multipotent progenitors instruct ontogeny of the superior colliculus. *Neuron* (2023). https://doi.org:10.1016/j.neuron.2023.11.009

13 Lim, D. A. & Alvarez-Buylla, A. The Adult Ventricular-Subventricular Zone (V-SVZ) and Olfactory Bulb (OB) Neurogenesis. *Cold Spring Harb Perspect Biol* **8** (2016). https://doi.org:10.1101/cshperspect.a018820

14 Kohwi, M. *et al.* A subpopulation of olfactory bulb GABAergic interneurons is derived from Emx1- and Dlx5/6-expressing progenitors. *The Journal of neuroscience : the official journal of the Society for Neuroscience* **27**, 6878-6891 (2007). https://doi.org:10.1523/JNEUROSCI.0254-07.2007

15 Lledo, P. M., Merkle, F. T. & Alvarez-Buylla, A. Origin and function of olfactory bulb interneuron diversity. *Trends Neurosci* **31**, 392-400 (2008). https://doi.org:10.1016/j.tins.2008.05.006

16 Beattie, R. *et al.* Mosaic Analysis with Double Markers Reveals Distinct Sequential Functions of Lgl1 in Neural Stem Cells. *Neuron* **94**, 517-533 e513 (2017). https://doi.org:10.1016/j.neuron.2017.04.012

17 Gao, P. *et al.* Deterministic progenitor behavior and unitary production of neurons in the neocortex. *Cell* **159**, 775-788 (2014). https://doi.org:10.1016/j.cell.2014.10.027

18 Kriegstein, A. & Alvarez-Buylla, A. The glial nature of embryonic and adult neural stem cells. *Annual review of neuroscience* **32**, 149-184 (2009). https://doi.org:10.1146/annurev.neuro.051508.135600

Response to Reviewers – Stouffer et al. Nature

General statement:
We are very pleased by the positive feedback from all reviewers. We greatly appreciate their consideration, constructive comments and suggestions, which we believe will help strengthen our paper even more. We have therefore carefully revised the manuscript to address all the remaining reviewers' suggestions, made all the editorial adjustments requested, and ensured full compliance with the journal's guidelines.

Point-by-point response (the original reviews are copied in blue)**:**

Referee #1 (Remarks to the Author):
The authors have responded thoughtfully and substantially to my concerns. The addition of in vivo developmental scRNA-seq, MADM-CloneSeq, expanded clone quantification, glial analysis, and improved assessment of cell death significantly strengthens the manuscript. In particular, the MADM-CloneSeq data directly address a major concern about clone identity in the absence of cortical lamination. I do not think further major experiments are required, provided the revised figures support the claims described in the rebuttal. I have a few remaining points that could be handled through textual clarification.

First, the Emx1-CreER validation should be framed as supporting comparable Emx1-lineage labeling rather than proving strict RGP specificity unless direct early post-induction co-labeling data are shown.
We thank the reviewer for the comment related to *Emx1*-Cre validation. We addressed the point accordingly and changed the respective sentence to *"… Emx1^{+} RGPs and their entire prospectively-labeled lineage-related progeny …"* to indicate that the data support comparable *Emx1*-lineage labeling.

Second, clone architecture categories in organoids should be described as operational MADM-defined clone classes, since spatial and laminar landmarks remain incomplete.
The reviewer indicates a valid point. To accommodate the issue, we now refer to clone types as operational MADM-defined clone classes and clarified that spatial and laminar landmarks are incomplete. We adapted the text as follows: *"Therefore, we next analyzed MADM clones to infer progenitor division mode (Fig. 2h-j and Ext. Data Fig. 13) in cortical organoids by applying the same MADM-defined clone classes, which remain applicable despite incomplete layering, as in previous in vivo experiments."*

Third, the trajectory analysis should be interpreted more cautiously: the absence of a separate transcriptional RGP trajectory does not exclude subtle progenitor heterogeneity or niche-dependent competence states.
We agree with the reviewer. The text has thus been revised to adopt a more cautious interpretation of trajectory analysis. It now reads, *"We performed UMAP clustering and trajectory analysis using slingshot and identified a single unitary trajectory connecting RGPs via IPs to*

neurons, which was consistent using several different UMAP and clustering parameters (Fig 4j and Ext. Data Fig. 25). However, we do not exclude the possible presence of minor diverging sub-lineages or subtle progenitor heterogeneity."

Finally, claims that cell death is not involved and that gliogenesis proceeds "faithfully" should be softened to reflect the limits of the assays.
We thank the reviewer for pointing out the above items. We addressed them as follows:
Cell Death: We carefully went through the manuscript and only found one careful mentioning of cell death which we further softened. The respective sentence now reads, *"Thus, cell death seems not to be a major factor influencing the interpretation of our data, albeit we cannot exclude that sparse, sporadic cell death may eliminate some cells. Nevertheless, small neurogenic clones represent an organoid-specific clone type not observed in vivo".*
Gliogenesis: We replaced the word *faithfully* with *sequentially* to reflect the limits of the system.

Overall, I find the revision strong and the remaining issues largely matters of interpretation and precision.
We thank and greatly appreciate the reviewer for the positive evaluation of our revision and for the thoughtful and constructive feedback. We have now modified the text accordingly to fully accommodate the above remaining points.

<u>Referee #2 (Remarks to the Author):</u>
The revised manuscript has been substantially strengthened and the major concerns raised in the initial review have been addressed with extensive additional experiments, analyses, and clarification throughout the text. In particular, the addition of matched in vivo scRNA-seq datasets, improved lineage-tracing analyses, expanded validation of ESC lines, additional MADM-CloneSeq experiments, and improved scRNA-seq analyses significantly enhance the rigor and interpretability of the study.
The manuscript now provides a compelling comparative framework between in vivo corticogenesis and cortical organoid development, and the central conclusion - namely that organoids can recapitulate major cell states and broad developmental ordering while diverging in lineage progression dynamics and lineage restriction behavior - is generally well supported by the presented data. It is also appreciated that several interpretations were refined in response to reviewer comments, particularly regarding neuronal subtype annotations and trajectory analyses.
We thank the reviewer the positive and encouraging evaluation of our revision.

Only a few remaining points should be addressed during final revision:

1. While the additional in vivo matched scRNA-seq dataset and label-transfer analyses substantially strengthen the developmental comparison between organoids and embryonic cortex, the proposed temporal alignment between organoid differentiation stages and in vivo development remains only partially supported. The conclusion that D8, D13, D20 and D25 correspond closely to E10, E13, E16 and P1 is currently based largely on cell-type abundance, integrated visualization, and marker overlap analyses, which demonstrate broad correspondence

in major cell states and developmental ordering but do not establish transcriptome-wide equivalence across matched developmental stages.

We thank the reviewer for this careful assessment of our analysis and agree that transcriptome-wide analysis of cell-type similarity may give valuable insights in the alignment of developmental progression *in vivo* and *in vitro*. To this end, we performed the revision as indicated below in Point 2.

2. This temporal alignment is central to interpretation of the clonal analyses. The in vivo MADM framework used for comparison relies on embryonic induction (E10-E13) followed by postnatal analysis (P7-P21/P30), whereas the organoid paradigm uses induction between D8-D15 with collection at D20. Although the revised analyses support approximate correspondence between organoid and embryonic developmental stages up to approximately P1-equivalent states, it remains unclear whether the overall developmental pace and maturation state of the in vitro paradigm are directly comparable to the substantially later postnatal in vivo clonal analyses used as a reference framework. A more quantitative transcriptome-wide comparison or a reference mapping approach between organoid and in vivo time points (for example a pseudobulk correlation analyses across shared genes, ideally by developmental stage, or transferred labels) would substantially strengthen these conclusions. Otherwise, and it would be sufficient to this reviewer, that claims of "nearly congruent" developmental trajectories and close resemblance to in vivo corticogenesis should be moderated accordingly.

To address the above important point, we have now directly compared transcriptomes across developmental time points. We performed a transcriptome-wide correlation analysis between our organoid data and a range of developmental stages from the La Manno reference data (La Manno et al., 2021 Nature 596, 92-96) set, ranging from E9-E18. In order to directly compare correlation scores across multiple comparisons, we calculated a dissimilarity score, visualized as a heatmap in (Extended Data Figure 4E). Overall the data indicates that the D8 RGPs (organoids) show the highest similarities to E9/E10 RGPs (La Manno et al., 2021 Nature 596, 92-96); D20 and D25 neurons (organoid) show the highest similarity to E16/E17 and E18 Neurons (La Manno et al., 2021 Nature 596, 92-96) respectively, corroborating our previous developmental time matching analysis. Yet, we also rephrased the respective sentence and replace "nearly congruent" with "remarkably similar".

3. The clonal analyses clearly demonstrate that multiple progenitor division modes coexist across organoid differentiation stages and that lineage progression is less temporally constrained than in vivo. However, the data still show gradual temporal shifts in clone size and clone-type abundance across differentiation, suggesting that developmental progression is not fully equipotent or temporally invariant. Since pairwise comparisons between consecutive time points may underestimate gradual developmental trends, statistical approaches that directly test global temporal changes across differentiation stages (for example regression- or trend-based analyses) should be considered. The results of these analyses will confirm whether terms such as "equipotent cell division capacity" or "loss of temporal stereotypy" are directly supported by the data Alternatively, the corresponding interpretations should be moderated accordingly.

We thank the reviewer for this insightful suggestion. In the revised manuscript, we use a generalized linear mixed model in combination with a likelihood ratio test to show a significant

effect of age on clone abundance. Note that despite these significant changes within the organoid data set, most relevant for our message are the differences of clone type abundance to mouse *in vivo* cortex. We have revised the text in order to moderate the interpretation of the data: *"Despite a progressive decrease in asymmetric clone size and an absence of small neurogenic clones in vivo during the neurogenic phase [i.e. E12-E16], the reduction in size of asymmetric and small neurogenic clones in organoid were less pronounced over time (Fig. 2i-j; Ext. Data Fig. 16)."* Furthermore, we followed the reviewer's suggestion and moderated our text. We now refrain from using 'equipotent cell division capacity' and avoid the use of 'loss of temporal stereotypy'.

Overall, the revised manuscript represents a significant contribution to the field of cortical organoid biology and developmental neurobiology, and publication is supported after these minor clarifications of the remaining points above.
We thank the reviewer and highly appreciate the positive evaluation of our revision. We also appreciate the thoughtful remaining points that we have now fully addressed in the above responses and with according textual modifications in the revised manuscript.

Referee #3 (Remarks to the Author):
I co-reviewed this manuscript with one of the reviewers who provided the listed reports.

Referee #4 (Remarks to the Author):
The authors have undertaken an impressive and comprehensive body of work, combining both wet-lab and in silico approaches, which effectively addresses all of my major concerns. The revisions have substantially strengthened the manuscript, which now reads clearly and presents a scientifically compelling study. I have only a few remaining minor suggestions.
We greatly appreciate the positive evaluation our revision and encouraging feedback. Below we fully adress the remaining minor suggestions.

1. Title: Consider including the term "brain" or "cerebral" (e.g., "…self-organizing cerebral organoid…").
We appreciate the reviewer's suggestion. We have added "cortical" rather than "brain" or "cerebral," as our study focuses specifically on cortical organoids, and to comply with the guidelines (max. character count in title). The new title reads: *'Temporal Uncoupling of Radial Glia Lineage Progression in Cortical Organoid'*

2. Page 3, line 2: Replace "mayor" with "major."
We replaced "mayor" with "major" according to the reviewer's suggestion.

3. Page 6, line 8: It would be helpful to clarify—based on cellular and molecular evidence—that D10 corresponds approximately to E10 for comparative purposes.
We have followed the reviewer's suggestion and modified the text accordingly as follows: *"Proliferative clone size in organoids decreased gradually over time from 44.17 ± 4.5 at D8 to 14.46 ± 2.1 at D13 (Fig. 2h), and was almost half the size on average at D10 (37.07 ± 3.69*

cells/clone) when compared to proliferative clones in vivo at E10 [71.2 ± 8.3 cells/clone (Ext. Data Fig. 14);[1], note: E10 and D10 are comparable transcriptionally (Ext. Data Fig. 4e)].”

4. Page 7: I suggest removing the word “powerful” from the last paragraph.
We removed two instances of “powerful” in the main text

5. Figure 2: I recommend removing the “ns” annotations above histograms h–j, as none of the comparisons are non-significant; this can instead be clearly stated in the figure legend.
In the revised manuscript, we have performed generalized linear mixed model in combination with a likelihood ratio test to better describe the data.
